# Excessive MYC Orchestrates Macrophages induced Chromatin Remodeling to Sustain Micropapillary‐Patterned Malignancy in Lung Adenocarcinoma

**DOI:** 10.1002/advs.202403851

**Published:** 2025-02-03

**Authors:** Xuming Song, Zehao Pan, Yi Zhang, Wenmin Yang, Te Zhang, Hui Wang, Yuzhong Chen, Xinnian Yu, Hanlin Ding, Rutao Li, Pengfei Ge, Lin Xu, Gaochao Dong, Feng Jiang

**Affiliations:** ^1^ Department of Thoracic Surgery Nanjing Medical University Affiliated Cancer Hospital & Jiangsu Cancer Hospital & Jiangsu Institute of Cancer Research Nanjing 210009 P. R. China; ^2^ Jiangsu Key Laboratory of Molecular and Translational Cancer Research Cancer Institute of Jiangsu Province Nanjing 210000 P. R. China; ^3^ The Fourth Clinical College of Nanjing Medical University Nanjing 210000 P. R. China; ^4^ Department of Pathology Nanjing Medical University Affiliated Cancer Hospital & Jiangsu Cancer Hospital & Jiangsu Institute of Cancer Research Nanjing 210009 P. R. China; ^5^ Department of Pathology Nanjing Drum Tower hospital Nanjing 210008 P.R. China; ^6^ Department of Biochemistry and Molecular Genetics Feinberg School of Medicine Northwestern University Chicago Illinois 60201 USA; ^7^ Department of Thoracic Surgery The Fourth Affiliated Hospital of Soochow University Nanjing 215000 P. R. China; ^8^ Collaborative Innovation Center for Cancer Personalized Medicine Nanjing Medical University Nanjing 211116 P. R. China

**Keywords:** chromatin accessibility, M2‐like macrophages, micropapillary lung adenocarcinoma, MYC

## Abstract

Current understanding of micropapillary (MP)‐subtype lung adenocarcinoma (LUAD) remains confined to biological activities and genomic landscapes. Unraveling the major regulatory programs underlying MP patterned malignancy offers opportunities to identify more feasible therapeutic targets for patients with MP LUAD. This study shows that patients with MP subtype LUAD have aberrant activation of the MYC pathway compared to patients with other subtypes. In vitro and xenograft mouse model studies reveal that MP pattern in malignancy cannot be solely due to aberrant MYC expression but requires the involvement of M2‐like macrophages. Excessively expressed MYC leads to the accumulation of M2‐like macrophages from the bone marrow, which secretes TGFβ, to induce the expression of FOSL2 in tumor cells, thereby remodeling chromatin accessibility at promoter regions of MP‐pattern genes to promote the MYC‐mediated *de novo* transcriptional regulation of these genes. Additionally, the MP‐pattern in malignancy can be effectively alleviated by disrupting the TGFβ‐FOSL2 axis. These findings reveal new functions for the M2‐like macrophage‐TGFβ‐FOSL2 axis in MYC‐overexpressing MP‐subtype LUAD, identifying targetable vulnerabilities in this pathway.

## Graphical abstract

Song et al. integrates an evaluating system to characterize malignancy of micropapillary (MP)‐pattern lung adenocarcinoma in vivo or in vitro. They have discovered that intrinsic MYC redundancy expression combined with extrinsic M2‐like macrophage jointly mediate MP‐pattern malignancy. Mechanically, M2‐like macrophages open MP‐pattern gene chromatin structure and recruit MYC to cooperatively regulate transcription.

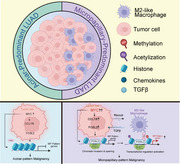

## 1 Introduction

Lung adenocarcinoma (LUAD) is a morphologically and genomically heterogeneous disease, whose morphological features are related to tumor biological behavior.^[^ ^1^ ^]^ According to growth patterns, LUAD are categorized as low‐pattern ‘lepdic’, mid‐pattern ‘papillary’, ‘acinar’ (AC) and high‐ pattern ‘micropapillary’ (MP),’solid.’^[^ ^2^ ^]^ The MP‐subtype carcinoma, which has morphologically distinct forms, grows in papillary tufts forming florets that lack fibrovascular scaffolds.^[^ ^3^ ^]^ Extensive research shows a strong correlation between MP‐subtype carcinoma and lymph node invasion,^[^ ^4^, ^5^ ^]^ as well as recurrence and metastasis in early stage carcinoma.^[^ ^6^ ^]^ Even a minimal presence of MP‐subtype carcinoma significantly reduces overall survival and progression‐free survival in postoperative patients.^[^ ^7^, ^8^ ^]^

The strong invasive and metastatic ability of the MP‐subtype carcinoma has been attributed in numerous recent pathological studies, to its unique structure‐driven anchorage‐independent growth, characterized by typical molecular features such as epithelial‐mesenchymal transition (EMT),^[^ ^9^, ^10^ ^]^ resistance to anoikis,^[^ ^11^ ^]^ and alterations in cellular polarity.^[^ ^12^, ^13^ ^]^ The etiology underlying those molecular features were investigated through, genomic studies of MP‐subtype carcinoma. Previous studies have demonstrated that MP‐subtype carcinoma has higher tumor mutation burden, chromosomal instability, and intra‐tumoral heterogeneity compared to low/mid‐risk subtypes.^[^ ^1^, ^14^ ^]^ Recently, Shipeng et al. reported a higher incidence of MET proto‐oncogene, receptor tyrosine kinase (MET) amplification and mechanistic target of rapamycin kinase (MTOR) mutations in MP‐subtype LUAD.^[^ ^15^ ^]^ Fanchen et al. also reported the enrichment of AID/APOBEC mutations and activation of RTK/Ras, Notch, and Wnt pathways in MP‐subtype LUAD.^[^ ^16^ ^]^ Microdissection of DNA and RNA sequencing (RNA‐seq) analysis on multiple sampling points of 10 LUAD samples revealed that alterations in transcription patterns, rather than genomic changes, are the major drivers of growth pattern change.^[^ ^17^ ^]^ However, the underlying core transcriptional programs regulating numerous molecular processes in MP‐subtype LUAD remain poorly understood.

The tumor microenvironment serves as a significant source of intratumoral and intertumoral heterogeneity, directly influencing tumor behavior, progression, and response to treatment.^[^ ^18^, ^19^, ^20^ ^]^ Recently, large‐scale single‐cell spatial proteomics studies have provided rich data on the tumor microenvironment across different pathological subtypes of LUAD. Within the MP‐subtype LUAD, there is an abundance of CD163+ macrophages interacting with tumor cells.^[^ ^21^ ^]^ A study by Yan et al. study also showed that MP‐subtype LUAD had higher CD68 expression compared to the low/mid‐risk subtypes.^[^ ^22^ ^]^ However, the distribution and functional roles of various immune cells in the vicinity of MP‐subtype LUAD remain unclear.

In this study, we integrated a cohort of 66 LUAD cases predominantly including MP‐subtype LUAD and utilized microdissected RNA‐seq data to find aberrantly activated MYC pathway. Inconsistent experimental results between in vivo and in vitro experiments led us to hypothesize that MYC requires the involvement of M2‐like macrophages‐associated FOSL2 to bind *de novo* H3K27ac‐occupied sites of remodeled chromatin, thereby regulating the transcription of MP‐pattern genes. Overall, we identified a regulatable mechanism involving both intracellular and extracellular regulation that orchestrates the sustain of MP‐pattern in LUAD.

## 2 Results

### 2.1 The MP‐Subtype LUAD is Associated with MYC Pathway Activation

**Figure 1:**
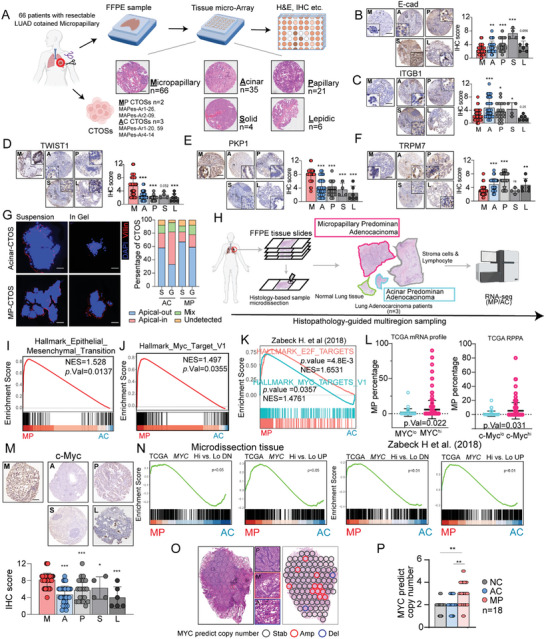
MP‐subtype LUAD exhibits distinct tumor behavioral changes and is potentially closely associated with MYC pathway. A) A schematic illustration showing sample composition of the MAPes cohort. Scale bars: 100 µm. B–F) Representative images of immunohistochemistry of epithelial cell marker E‐cad (B), mesenchymal cell marker TWIST1 (D), anti‐anoikis marker ITGB1 (C), anti‐shearing force marker PKP1 (E) and shear stress sensor marker TRPM7 (F) in tissues micro‐array from MAPes cohort. n = 66. Scale bars: 100 µm. G) Representative images of immunofluorescence staining of CTOSs derived from freshly dissociated MP‐subtype and AC‐subtype tumor tissue in suspended conditions (suspended) and embedded in Matrigel ECM and cultured for 72 h (in gel). Red, villin; blue, DAPI. Scale bars: 20 µm. Right: Stacked bar graph depicting number of CTOSs with different apical status. n = 10. H) A schematic illustration showing strategies of bulk RNA‐seq after microdissection of different pathological subtypes from the same patient. I,J) Gene Set Enrichment Analysis (GSEA) of different expression genes between MP‐subtype and AC‐subtype tissue from RNA‐seq after microdissection in (H,K)) GSEA of different expression genes between MP‐subtype and AC‐subtype tissue bulk RNA‐seq from Zebeck H et al. study. L) Representative images of immunohistochemistry of c‐Myc in tissues micro‐array from MAPes cohort. n = 66. M,N) The difference of percentage of MP‐subtype invasion area between MYC‐high expression and MYC‐low expression samples from TCGA‐LUAD mRNA dataset (M) Scale bars: 100 µm, moreover, between c‐Myc high expression and c‐Myc‐low expression samples from TCGA‐LUAD RPPA dataset (N). O) GSEA of different expression genes between of MYC‐high expression (Top 15%) and MYC‐low expression (bottom 15%) samples from TCGA‐LUAD mRNA dataset. Gene sets were constructed by different expression of MP‐subtype and AC‐subtype tissue bulk RNA‐seq from RNA‐seq after microdissection in (H) (upper) and Zebeck H et al. study (bottom). P,Q) The copy number detected by TaqMan FAM‐labeling probe from mutli‐subtypes samples in‐site sampling (P) and respective microdissection (Q). n = 18. The p values were determined by Student's *t*‐test (unpaired two‐tailed), n.s., not significant; *p < 0.05, **p < 0.01, ***p < 0.001. Data are represented as mean ± SEM.

The assessment of MP adenocarcinoma has long relied on morphology and has been difficult to mimic in animal models.^[^ ^13^ ^]^ However, extensive pathological studies focused on its malignant behavior have elucidated partial aspects of its molecular mechanisms.^[^ ^10^, ^13^, ^23^, ^24^ ^]^ A Micropapillary Adenocarcinoma Pathology Retrospective cohort (hereinafter referred to as MAPes cohort, Table S1, Supporting Information) consisting of 66 MP‐subtype LUAD tissue samples and corresponding non‐MP subtype tissues (35 cases of AC subtype tissues, 21 cases of papillary subtype tissues, 4 cases of solid subtype tissues, and 6 cases of lepidic tissues) from the same patients, which were embedded in a tissue microarray (TMA) to validate the previously described molecular characteristics of micropapillary carcinoma (**Figures** **1**A, and S1A–C, Supporting Information). Immunohistochemical (IHC) analysis was used to detect the expression of EMT markers, including E‐cadherin (E‐cad), TWIST1, and the anti‐anoikis marker integrin subunit beta 1 (ITGB1)^[^ ^25^ ^]^ in the TMA. Consisting with previous studies,^[^ ^10^, ^11^, ^13^, ^23^, ^24^ ^]^ we found that the expression levels of E‐cad and ITGB1 were lower in MP‐subtype cores, whereas TWIST1 expression was higher in MP‐subtype cores compared to other subtypes (Figure 1B–D). Recent studies have revealed a higher detection rate of circulating tumor cells (CTCs) in peripheral blood of patients with MP‐subtype LUAD compared to that in patients with solid‐subtype LUAD.^[^ ^26^, ^27^ ^]^ This finding suggests the need to investigate key factors associated with CTCs in MP subtype samples, specifically focusing on shear stress resistance biomarkers. The anti‐shear force markers Plakophilin 1 (PKP1)^[^ ^28^ ^]^ and shear force sensor TRPM7^[^ ^29^ ^]^ were also found to be upregulated and downregulated, respectively, in the MP‐ subtype LUAD (Figure 1E,F). Intriguingly, the AC‐subtype, which is the most common histological subtype of LUAD,^[^ ^30^ ^]^ appears to have characteristics that are contrary to the aforementioned MP‐patterned malignancy. In particular, we observed distinct cell polarity features during short‐term culturing of cancer tissue‐originated spheroids (CTOSs) derived from the MP‐ and AC‐subtypes. Instead, the AC‐derived CTOSs exhibited apical membrane polarity switching, a feature commonly observed in other adenocarcinomas.^[^ ^31^ ^]^ Such phenomenon was not triggered in MP‐derived CTOSs, which is consistent with the unique features of MP‐like tumors^[^ ^13^ ^]^ (Figure 1G).

To elucidate the potential regulatory mechanism underlying molecular characteristic of MP‐patterned malignancy, we performed microdissection‐assisted RNA‐seq analysis on MP‐subtype tissue samples and corresponding AC subtype tissue samples from the same patients (Figure 1H, Table S2, Supporting Information), and found distinct transcriptional features (Figure S1D, Supporting Information). As expected, MP‐subtype tissue samples showed a more prominent tendency toward EMT, anti‐anoikis, and resistance to shear forces (Figure 1I, Figure S1E, Supporting Information). These findings are consistent with the results reported by Zabeck et al.^[^ ^32^ ^]^ (Figure S1F,G, Supporting Information). Noteworthy, both our microdissection‐assisted RNA‐seq, in conjunction with the study conducted by Zabeck et al. and The Cancer Genome Atlas (TCGA)‐LUAD mRNA data, revealed a significant activation of the MYC pathway in the MP‐subtype LUAD compared to that in the AC subtype (Figure 1J,K, Figure S1H,I, Supporting Information). The protein levels of c‐Myc and its molecular partner Max were found to be significantly increased in MP‐subtype tissue samples, as evidenced by the MAPes cohort TMA data (Figure 1L Figure S1J, Supporting Information). Furthermore, analysis of TCGA‐LUAD mRNA and Reverse phase protein array (RPPA) datasets revealed that tissue samples with high MYC expression level had increased distribution of MP‐subtype (Figure 1M, Table S3, Supporting Information). Tissue samples with high MYC expression level in TCGA‐LUAD mRNA dataset showed a greater tendency toward the characteristics of MP‐subtype LUAD (Figure 1N). Therefore, we hypothesize that there is a strong association between MP‐ subtype LUAD and activation of the MYC pathway.

MYC dysregulation is frequently observed in human cancers, including LUAD.^[^ ^33^ ^]^ thereby initiating or sustaining tumor growth.^[^ ^34^ ^]^ MYC dysregulation or MYC amplification is commonly observed in LUAD and can occur at any stage of tumor development.^[^ ^35^, ^36^ ^]^ We analyzed the whole‐exome sequencing data from TCGA‐LUAD dataset and found increased copy number amplification of the MYC gene locus (8q24.21) in patients with MP‐subtype (Figure S1K, Supporting Information). In situ MYC copy number probe analysis performed on a LUAD paraffin section containing multiple histological subtypes revealed a focal amplification of MYC copies in the infiltrating regions of the MP‐subtype (Figure 1O). In addition, microdissection‐assisted MYC copy number probe analysis of 33 samples from the MAPes cohort that included both MP‐ and AC‐subtypes, revealed a significant increase in MYC copy numbers in the MP‐subtype (Figure 1P). Taken together, these data revealed that the MP‐pattern in LUAD patient may be associated with the copy number variation induced‐MYC pathway activation.

### 2.2 MYC Potentially Collaborates With Extracellular Factors to Collectively Mediate the Development of MP‐Patterned Malignancy

Given the positive association between the MYC pathway and MP‐subtype in LUAD, we further investigated whether ocerexpression of MYC directly contributes to the phenotype of MP‐pattern in LUAD. However, using the published dataset (GSE210029), we hardly observe a transcriptional profile that favored the MP‐subtype in LUAD cells transfected with MYC (Figure S2A, Supporting Information). We also successfully over‐expressed or knocked out MYC expression in multiple LUAD cell lines (Figure S2B, Supporting Information), and measured the expression of marker genes of MP‐patterned LUAD. We found uncovered that the presence or absence of MYC in LUAD cell lines did not significantly alter both mRNA and protein expression levels of markers associated with EMT,^[^ ^37^ ^]^ cell adhesion,^[^ ^25^ ^]^ and resistance to circulatory system shear stress^[^ ^28^, ^29^ ^]^ (Figure S2C,D, Supporting Information). Moreover, manipulating MYC expression in LUAD cell lines failed to alter the pre‐existing anti‐apoptotic features and shear stress response (Figure S2E–J, Supporting Information). The LUAD cell lines similarly exhibited an “apical‐out” phenotype in suspension culture and an “apical‐in” phenotype in Matrigel culture, and overexpression of MYC did not alter this polarity switching phenomenon (Figure S2K, Supporting Information).

**Figure 2:**
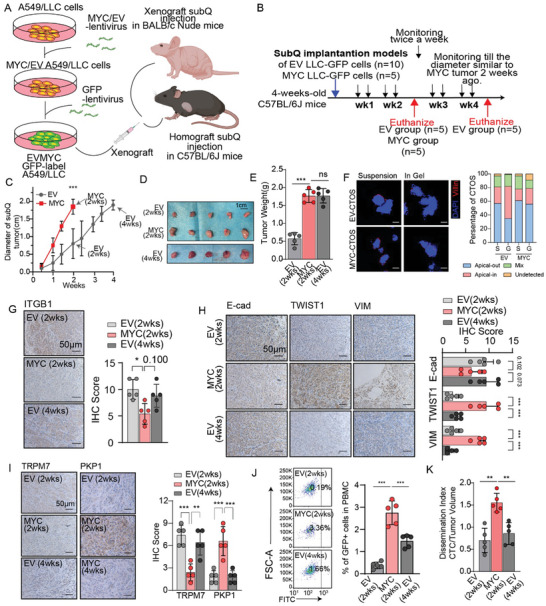
MP‐pattern malignancy was induced by redundant MYC expression tumor cells in syngeneic transplant mice. A,B) An experimental illustration showing phenotypic characterization of MYC redundant expression tumors in athymic BALB/c Nude mice and immunocompetent C57BL/6J mice (A). Experimental strategies for the C57BL/6J mouse homotransplantation model (B). C–E) Growth kinetics (C), tumor images (D) and weight of tumor (E) of subcutaneously implanted tumors from indicated C57BL/6J mice. n = 5 per groups. F) Representative images of immunofluorescence staining of CTOSs derived from freshly dissociated from 2‐week‐growth MYC‐overexpression tumor and 4‐week‐growth MYC‐basal expression tumor in suspended conditions (suspended) and embedded in Matrigel ECM and cultured for 72 h (in gel). Red, villin; blue, DAPI. Scale bars: 20 µm. Right: Stacked bar graph depicting number of CTOSs with different apical status. G–I) Representative images of immunohistochemistry of TRPM7, PKP1 (G), ITGB1 (H), E‐cad, TWIST1 and VIM (I) in subcutaneously implanted tumors from 2‐week‐after‐implanted MYC‐basal expression, 2‐week‐after‐implanted MYC‐overexpression and 4‐week‐after‐implanted MYC‐basal expression C57BL/6J mice. n = 5 per groups. Scale bars: 50 µm. J) Circulating tumor cells (CTCs) detected from venous blood in 2‐week‐after‐implanted MYC‐basal expression, 2‐week‐after‐implanted MYC‐overexpression and 4‐week‐after‐implanted MYC‐basal expression C57BL/6J mice. Left: Representative images, right: quantitative statistics. n = 5 per groups. K) Dissemination index, which represents the ratio of circulating tumor cells in venous blood to tumor volume for assessing primary tumor dissemination, in 2‐week‐after‐implanted MYC‐basal expression, 2‐week‐after‐implanted MYC‐overexpression and 4‐week‐after‐implanted MYC‐basal expression C57BL/6J mice. n = 5 per groups. The p values were determined by Student's *t‐*test (unpaired two‐tailed), n.s., not significant; *p < 0.05, **p < 0.01, ***p < 0.001. Data are represented as mean ± SEM.

These in vitro results were inconsistent with the in vivo experimental results (**Figure** **2**A). In immunocompetent C57BL/6J mice, Lewis lung carcinoma (LLC) cells mouse lung cancer xenograft model, the overexpression of MYC significantly accelerated tumor proliferation. By monitoring tumor size, we found that the tumor size in the MYC overexpression group at 2 weeks was similar to that in the control groups at 4 weeks (Figure 2B–E, Supporting Information). In subcutaneous tumor‐derived CTOSs, at basal MYC expression level, CTOSs showed polarity switching similar to the AP subtype and LUAD‐cell lines under different culture conditions, while MYC‐overexpressing CTOSs exhibited resistance to polarity switching like MP‐subtype CTOSs (Figure 2F). IHC analysis of a subcutaneous tumor also revealed that a MYC‐overexpressing tumor highly exhibited MP markers, including markers of EMT, cell adhesion, and resistance to shear stress, but this which was not solely attributed to tumor volume (Figure 2G–I, Figure S3A, Supporting Information). Recent research has shown that the detection rate and quantity of circulating tumor cells (CTCs) are significantly increased in the presence of the MP‐subtype LUAD.^[^ ^38^ ^]^ Overexpression of MYC in tumor cells significantly increased the presence of CTCs in the peripheral blood of mice (Figure 2J,K, Figure S3B, Supporting Information). Notable, in a human lung cancer A549 cell line xenograft model in immunodeficient BALB/c nude mice, overexpression of MYC promoted tumor proliferation, but did not significantly increase the dissemination of tumor cells (Figure S3C–H, Supporting Information). In addition, both MYC‐overexpressing CTOSs and MYC basal‐expressing CTOSs did not exhibit resistance to MP‐pattern polarity switching (Figure S3I, Supporting Information). Similar results were also observed in a perivitelline xenograft assay using 2‐day post fertilization (dpf) zebrafish larvae, which did not show any alteration in tumor cell dissemination ability upon MYC overexpression (Figure S3J,K, Supporting Information). These findings prompted speculation that MP‐patterned malignancy switching mechanisms could involve MYC overexpression as well as extracellular factors in the tumor microenvironment, such as intraspecific immune system responses.

### 2.3 Synergistic Induction of MP‐Patterned Malignancy by MYC and M2‐Like Macrophages

**Figure 3:**
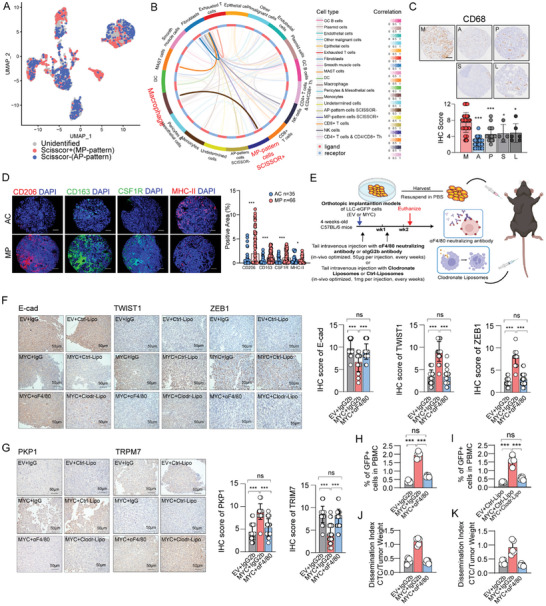
The induction of MP‐pattern malignancy relies on macrophages in syngeneic transplant mice with bearing redundant MYC expression tumor. A) Single‐cell RNA sequencing (scRNA‐seq) data of lung adenocarcinoma from Michael Bartoschek et al. study was utilized. Malignant epithelial cells were selected, and mapping was performed based on bulk RNA‐seq data following microdissection, as shown in Figure 1H. Two distinct cell populations were identified: SCISSOR+ cells (MP‐subtype) and SCISSOR‐ cells (AC‐subtype). B) Circos plot showing the cell‐cell interactions in cell clusters from(A) scRNA‐seq after mapping by SCISSOR algorithm. The thickness of each string indicates the number of different interaction pairs colored by cell clusters. C) Representative images of immunohistochemistry of macrophage marker CD68 in tissues micro‐array from MAPes cohort. D) Representative images of immunohistochemistry of adenocarcinoma marker TTF1, and multiple immunofluorescences of CD206 (red), CD68 (green) and DAPI (blue) from FFPE slide with mutli‐subtypes samples. E) A schematic illustration showing two distinct strategies depleting macrophages in mice with MYC redundant expression tumors. F,G) Representative images of immunohistochemistry of E‐cad, TWIST1 and ZEB1 (F), PKP1 and TRPM7 (G). right: quantitative statistics. Five random individual fields were selected from each pathological slide for the assessment of the 12‐point IHC score. Scale bars: 50 µm. H,I) CTCs detected from venous blood in indicated mice. J,K) Dissemination index in EV+IgG2b, MYC+IgG2b, MYC+αF4/80 group mice (J) and in EV+Ctrl‐Lipo, MYC+Ctrl‐Lipo, MYC+Clodr‐Lipo group mice (K). For each group, n = 5. The p values were determined by Student's *t*‐test (unpaired two‐tailed), n.s., not significant; *p < 0.05, **p < 0.01, ***p < 0.001. Data are represented as mean ± SEM.

In order to identify immune cells that participate with MYC in the maintenance of MP‐patterned malignancy in LUAD, we used the SCISSOR algorithm^[^ ^39^ ^]^ to map the results of microdissected bulk RNA‐seq analysis to single‐cell RNA‐seq data^[^ ^40^ ^]^ of Michael Bartoschek et al., 10 samples of LUAD tissue samples. A total of 747 SCISSOR+ (MP‐subtype biased) tumor cells and 739 SCISSOR‐ (AP‐subtype biased) tumor cells were mapped (**Figures** **3**A, and S4A, Supporting Information). SCISSOR+ tumor cells showed higher expression of MYC and EMT characteristics, consisting with the features we used to describe MP pattern (Figure S4B–D, Supporting Information). Previous studies have revealed that MP‐subtype LUAD has a higher infiltration of macrophages; however, the functional implications of these infiltrating macrophages remain unclear.^[^ ^21^, ^22^ ^]^ We also investigated the crosstalk between tumor cells and various defined immune cells using integrated receptor‐ligand interactions and a method measuring the activity of transcription factors.^[^ ^41^ ^]^ We observed that although all tumor cells suggested close associations with cancer‐associated fibroblasts (CAFs), indicating a widespread immunosuppressive state during LUAD proliferation,^[^ ^42^ ^]^ but only SCISSOR+ tumor cells had strong communication with macrophages compared to SCISSOR‐ tumor cells and unmapped tumor cells (Figure 3B). Consistent findings were also observed in the MAPes cohort TMAs, indicating abundant CD68+ cell infiltration around MP‐tissues compared to other pathological subtypes, while rare non‐lymphocytic and myeloid lineage cells were observed in the same area (Figure 3C, Figure S4E,F, Supporting Information). In addition, we analyzed by immunofluorescence (IF) staining the expression of M1 macrophage marker MHC II and M2 macrophage markers CD206, CD163 and CSF1R to determine the macrophage phenotype, and found high infiltration of CD206, CD163, and CSF1R‐positive cells and fewer MHC II–expressing cells around MP‐subtype tissues (Figure 3D). IF staining of CD68 and CD206 in LUAD tissues including multiple histological subtypes revealed an abundant infiltration of CD206+CD68+ cells in the MP‐subtype region, whereas other regions showed either no macrophage infiltration or minimal infiltration of CD206+CD68+ cells (Figure S4G, Supporting Information). Furthermore, we used deconvolution analysis to predict immune cells infiltration levels in TCGA‐LUAD mRNA dataset, and found that highly M2‐like macrophage infiltrated tumor tissues did not show a preference or statistically significant difference with the MP subtype tissues. However, in tumor tissues with high‐ levels of MYC expression and M2‐like macrophages, the proportion of MP‐subtype was centralized and significantly higher than other subgroups (Figure S5A, Supporting Information). As we observed in Figure 2A, MYC‐overexpressing tumor tissues also had higher infiltration of Arg1+ cell, but this was not observed in the xenograft tumors transplanted into BALB/c nude mice where MP‐patterned malignancy switching did not occur (Figure S5B, Supporting Information). These results may be attributed to blocking of recognition cytokines following signaling activation between different species, like human and mouse.^[^ ^43^ ^]^ Therefore, we hypothesize that MP‐patterned malignancy switching may be jointly mediated by MYC and M2‐like macrophages.

To elucidate the fundamental role of macrophages in MP‐patterned malignancy switching in MYC‐overexpressing LUAD cells, we employed anti‐F4/80 neutralizing antibodies or clodronate liposomes^[^ ^44^ ^]^ to systemically deplete macrophages in C57BL/6J mice (Figure 3E). Neither approach altered MYC‐overexpression induced subcutaneous tumor rapid proliferation (Figure S5C–F, Supporting Information). However, IHC analysis of subcutaneous tumors indicated that the depletion of macrophages in mice reduced the expression of markers, including markers of EMT, anti‐anoikis and anti‐shear forces (Figure 3F,G, Figure S5G–I, Supporting Information). Moreover, systemic depletion of macrophages significantly attenuated the detection of CTCs in peripheral blood, with a reduced dissemination index comparable to that of tumors with basal MYC expression (Figure 3H–K, Figure S5J,K, Supporting Information). The above results indicate that MYC overexpression alone is insufficient to induce MP‐patterned malignancy and requires the concurrent involvement of M2‐like macrophages.

**Figure 4:**
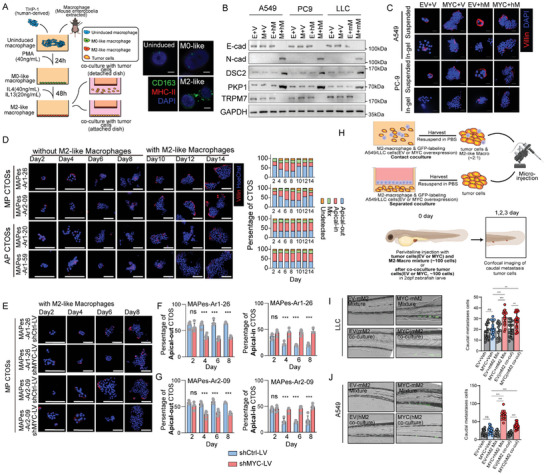
Redundant MYC expression along with M2‐like macrophages, synergistically induces MP‐pattern malignancy. A) Left: A schematic illustration showing an in vitro system was constructed by inducing M2‐like macrophages from THP‐1 monocytes or extracting macrophages from the peritoneal cavity of mice. Right: Representative images of multiple immunofluorescences of CD163 (green), MHC‐II (red) and DAPI (blue) in macrophage at each stage. B) Western Blots of EMT, cell adhesion, and shear force sensing marker in human and mouse LUAD cell lines. n = 3. C) Representative images of immunofluorescence staining of CTOS‐like cultured cell lines derived from indicated cells in suspended conditions (suspended) and embedded in Matrigel ECM and cultured for 72 h (in gel). Red, villin; blue, DAPI. Scale bars: 20 µm. D) Left: Representative immunofluorescence staining images of MP/AC‐subtype tissue from LUAD patients in MAPes cohort, embedded in Matrigel ECM and cultured for 14 days. MAPes‐Ar1‐26, MAPes‐Ar2‐09 are patients suffering from LUAD with over 70% MP‐subtype. MAPes‐Ar1‐20, MAPes‐Ar1‐59 are patients suffering from LUAD with over 70% AC‐subtype. Red, villin; blue, DAPI. Scale bars: 20 µm. Right: Stacked bar graph depicting number of CTOSs with different apical status. N = 5 per sample each observation day. E) Representative immunofluorescence staining images of MP‐subtype tissue from LUAD patients infected with lentivirus (LV) packaged plasmids systems (plasmid backbone and shMYC plasmid). Red, villin; blue, DAPI. Scale bars: 20 µm. F,G) Bar graph depicting number of apical‐out CTOSs (Left) and apical‐in CTOSs (Right) from Sample MAPes‐Ar1‐26 (F) and MAPes‐Ar2‐09 (G). N = 5 per sample. H) A schematic illustration showing the zebrafish larva perivitelline injection xenograft system for assessing tumor cell migration. I,J) Left: Representative images of larva tail tumor migration. Right: quantitative statistics of indicated cells. N = 16 per groups. The p values were determined by Student's *t*‐test (unpaired two‐tailed), n.s., not significant; *p < 0.05, **p < 0.01, ***p < 0.001. Data are represented as mean ± SEM.

To better understanding the underlying association between MYC and M2‐like macrophages, an in vitro co‐culture system was established as we have previously reported.^[^ ^45^ ^]^ In this study, M2‐like macrophages were differentiated from the human monocyte cell line THP‐1 or macrophages extracted from the peritoneal cavity of mice. These M2‐like macrophages were then co‐cultured in a non‐contact system with either human or mouse LUAD cells (**Figure** **4**A). In vitro experiments showed that the high‐MYC expression tumor cells co‐cultured with M2‐like macrophages (hereinafter referred to as M‐M2) significantly increased anoikis in LUAD cell and shear stress resistance compared to MYC overexpression alone (hereinafter referred to as M‐V) or other subgroups (Figure 4B, Figure S6A–E, Supporting Information). Furthermore, the M‐M2 groups significantly increasing the resistance of LUAD cell lines to apical membrane polarity switching in Matrigel culture (Figure 4C, Figure S6F, Supporting Information). To further elucidate the dual impacts of MP‐patterned malignancy involving MYC and surrounding M2 macrophages, we separately cultured LUAD patient‐derived MP‐CTOSs and AC‐CTOSs in Matrigel and dynamically observed the apical membrane polarity state. The results showed that when cultured alone, the apical membrane of MP‐CTOSs would switch to an “apical ‐in” state after 6 days growing in Matrigel. Upon co‐culture with M2 macrophages, the apical membrane of MP‐CTOSs quickly returned to the “apical‐out” state (Figure 4D). This apical membrane polarity switching was also regulated by MYC expression. In co‐culture with M2 macrophages, two lines of MP‐CTOSs derived from different patients all lost the “apical‐out” state of the apical membrane in Matrigel culture after knocking down MYC expression by lentiviral infection (Figure 4E–G). Additionally, two lines of AC‐CTOSs derived from different patients both exhibited an “apical‐out” state of the apical membrane in Matrigel upon co‐culture with M2 macrophages and infection with lentiviruses overexpressing MYC (Figure S6G–I, Supporting Information). LUAD cells were also co‐cultured with M2‐like macrophages either in contact or not in contact, and subsequently transplanted into the zebrafish xenograft model (Figure 4H). After 48 h, a notable occurrence of caudal fin metastasis was observed in the co‐cultured groups of tumor cells overexpressing MYC (Figure 4I,J), which suggested that intraspecific M2‐like macrophages can synergistically assist in tumor cells with excessive expression of MYC, thereby facilitating development of MP‐patterned malignancy. The above results collectively demonstrated that the MP‐patterned malignancy switching induced by MYC overexpression in LUAD cells was M2‐like macrophage dependent.

To investigate the origin of macrophages associated with MP‐patterned malignancy, CXCR4 and CCR2 were used to discriminate the difference between tissue‐resident macrophages and hematopoietic stem cell (HSC)‐derived macrophages.^[^ ^46^ ^]^ The results revealed that the infiltrating macrophages (F4/80+CD45+Ly6C‐) in murine lung orthotopic implanted tumors were predominantly of HSC‐derived origin (CXCR4‐CCR2+) (Figure S7A, Supporting Information), indicating that M2‐like macrophages participating in the formation of MP‐patterned malignancy were recruited from the circulatory system. Previous studies have determined that MYC can induce the transcription of CCL7 and CCL8, thereby mediating the local recruitment of macrophages within the tumor microenvironment,^[^ ^47^ ^]^ which were also observed in our quantitative real‐time polymerase chain reaction (qRT‐PCR) and enzyme‐linked immunosorbent assay (ELISA) results (Figure S7B,C, Supporting Information). Additionally, blocking CCL7 and CCR2 with the chemokine inhibitor Bindarit and the CCR2 antagonist INCB3344, respectively, effectively reversed the recruitment of M2‐like macrophages by MYC‐overexpressing tumor cells, suggesting that the recruitment was CCL7‐CCR2 dependent (Figure S7D, Supporting Information). Mechanistically, promoter regions of CCL7 and CCL8 were occupied by MYC, thereby inducing their expression (Figure S8A–D, Supporting Information). Interestingly, the MYC‐associated canonical signaling pathway was strongly activated in MYC‐KO cell line transfected with even a low dose of exogenous MYC plasmid, while the expression of non‐canonical target genes CCL7 and CCL8 indicated only moderate activation. Significant transcriptional activation of CCL7 and CCL8 was observed upon transfection with an adequate MYC plasmids (Figure S8E,F, Supporting Information). Similar changes were also observed with anti‐cMyc chromatin immunoprecipitation (ChIP)‐qPCR (Figure S8G, Supporting Information). Our results show that significant transcriptional activation of CCL7 and CCL8 occurs in the presence of MYC overexpression, possibly through the motif surrounding sequence.^[^ ^48^ ^]^

In summary, the transition and maintenance of MP‐patterned malignancy depend on the concurrent involvement of excessive MYC overexpression and M2‐like macrophages.

### 2.4 MYC in Collaboration with M2‐Like Macrophages Gains Access to Unique Binding Sites that Show Alternative Transcriptional Regulatory Activity

**Figure 5:**
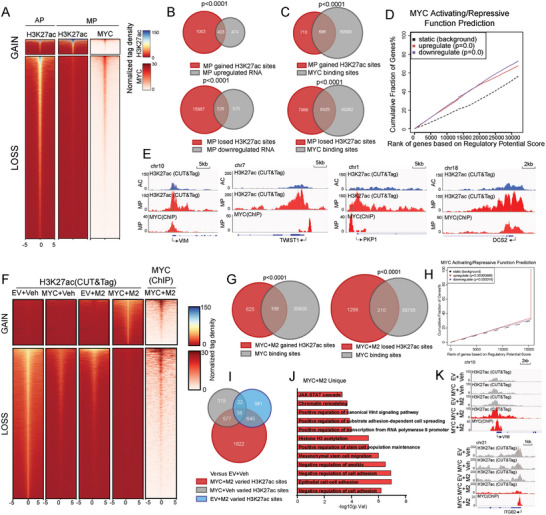
MYC in collaboration with M2‐like macrophages gains unique binding sites that exhibit alternative transcriptional regulatory activity. A) Heatmaps of H3K27ac CUT&Tag‐seq signals in MP‐subtype tissue and AC‐subtype tissue, c‐Myc ChIP‐seq signals in MP subtype tissue. H3K27ac modified sites were characterized as the those gained and lost based on the comparison of H3K27ac CUT&Tag‐seq results from AC‐subtype tissue and MP‐subtype tissue. Sites were ranked from the strongest to weakest H3K27ac binding for each category, shown in ±5‐kb windows centered at H3K27ac modified sites. B) Venn diagram shows substantial overlap of altered H3K27ac modified sites and significantly altered mRNA (*p*.value < 0.05, FoldChange≥2) in MP‐subtype and AC‐subtype tissues. C) Venn diagram shows substantial overlap of altered H3K27ac modified sites, which between MP‐subtype and AC‐subtype tissues, and MYC‐binding sites in MP‐subtype tissue. D) BETA of activating and repressive function of the MYC binding sites. The red, purple and black lines represent cumulative fractions of genes that are activated, unaffected or repressed by MYC (based on RNA‐seq results), respectively. The genes are ranked based on their regulatory potential scores (based on MYC ChIP–seq results). P values were calculated by two‐sided Kolmogorov–Smirnov tests. E) Representative H3K27ac CUT&Tag‐seq and MYC ChIP‐seq tracks, showing significantly altered H3K27ac occupied sites activity and MYC‐binding located periphery of MP‐pattern genes in MP‐subtype tissue versus AC‐subtype tissue. F) Heatmaps of H3K27ac CUT&Tag‐seq signals in E‐V cells, M‐V cells, E‐M2 cells and M‐M2 cells, moreover c‐Myc ChIP‐seq signals in M‐M2 cells. H3K27ac modified sites were characterized as the those gained and lost based on the comparison of H3K27ac CUT&Tag‐seq results from E‐V cells and M‐M2 cells. Sites were ranked from the strongest to weakest H3K27ac binding for each category, shown in ±5‐kb windows centered at H3K27ac modified sites. G) Venn diagram shows substantial overlap of altered H3K27ac modified sites, which between from E‐V cells and M‐M2 cells, and MYC‐binding sites in M‐M2 cells. H) BETA of activating and repressive function of the MYC binding sites in M‐M2 cells. P values were calculated by two‐sided Kolmogorov–Smirnov tests. I) Venn diagram shows substantial unique varied H3K27ac occupied sites in M‐M2 cells. Their changes did not overlap with those of MYC overexpression alone cells or co‐culture with M2‐like macrophages alone cells. J) Pathway enrichment of M‐M2 cells unique varied H3K27ac occupied sites associated genes. K) Representative H3K27ac CUT&Tag‐seq and MYC ChIP‐seq tracks, showing significantly altered H3K27ac occupied sites activity and MYC‐binding located periphery of MP‐pattern genes in E‐V cells, M‐V cells, E‐M2 cells and M‐M2 cells.

The involvement of MYC in transcriptional regulation was investigated by assessing the occupation of the histone modification marker H3K27ac by MYC in AC and MP subtype LUAD tissues. Substantially different epigenetic landscapes were identified in AC and MP subtype tissues (**Figure** **5**A). Through combined analysis of RNA‐seq data, loss of H3K27ac modification led to distinct transcriptional profiles observed between the AC‐subtype LUAD and the MP‐subtype LUAD (Figure 5B). In addition, MYC binding sites were frequently detected around these H3K27ac occupied sites with altered activity (Figure 5C). We also compared microdissection AP‐subtype LUAD and MP‐subtype LUAD RNA‐seq data from Figure 1H for further clarify the MYC effects at the transcriptional level. Notable, Binding and Expression Target Analysis(BETA)^[^ ^49^ ^]^ by Integrating RNA‐seq and MYC ChIP‐seq data revealed that MYC plays a double‐edged sword role in transcriptional regulation (Kolmogorov–Smirnov test, *p* < 1 × 10^−5^, Figure 5D). We further assessed MYC occupancy on promoter regions of MP‐pattern genes, including VIM and TWIST1, etc., suggesting that transcription of these genes was simultaneously highly activated with H3K27ac signal in the MP tissues, MYC significantly co‐occupying with H3K27ac promoter sites at MP‐pattern genes in MP tissues (Figure 5E). These findings indicated the extensive involvement of MYC in the epigenetic transcriptional regulation of MP‐pattern genes.

As shown in aforementioned results, overexpressing MYC and co‐culturing with M2‐like macrophages induced MP‐patterned malignancy switching in LUAD. We further investigated the roles of MYC and M2‐like macrophages in transcriptional activation changes of MP‐pattern genes by co‐culturing A549 cells or MYC‐overexpressing A549 cells with or without M2‐like macrophages, followed by RNA‐seq, H3K27ac Cleavage Under Targets and Tagmentation (CUT&Tag)‐seq, and MYC ChIP‐seq analyses (Figure 5F). Gene set enrichment analysis of MP‐pattern genes revealed that M‐M2 trained LUAD cells had favorable MP features, which is consistent with the phenotypic results (Figure S9A, Supporting Information). Similarly, M‐M2 trained LUAD cells also showed extensive differences in transcriptional regulation (Figure 5F,G). On the other hand, different from co‐cultured (E‐M2) or MYC overexpression alone (M‐V) cells, only M‐M2 trained cancer cells exhibited MP transcriptional landscape alterations in markers associated with MP characteristics, such as EMT and cell adhesion (Figure 5I,J). Moreover, MYC and H3K27ac preferred occupancy were different in MP‐pattern genes, such as VIM and ITGB2 (Figure 5K, Figure S9B, Supporting Information). Based on these findings, we proposed that MYC in MP tissue functions as a ‘chameleon’ to control the expression MP‐pattern genes expression by sensing surrounding infiltrating M2‐like macrophages, rather than perform a one‐way function.

### 2.5 The Transcriptional Regulatory Alterations of MP‐Pattern Genes Involve in Excessive MYC and M2‐Like Macrophage‐Derived Tgfβ‐Mediated Pioneer Transcription Factor FOSL2 Chromatin Remodeling

**Figure 6:**
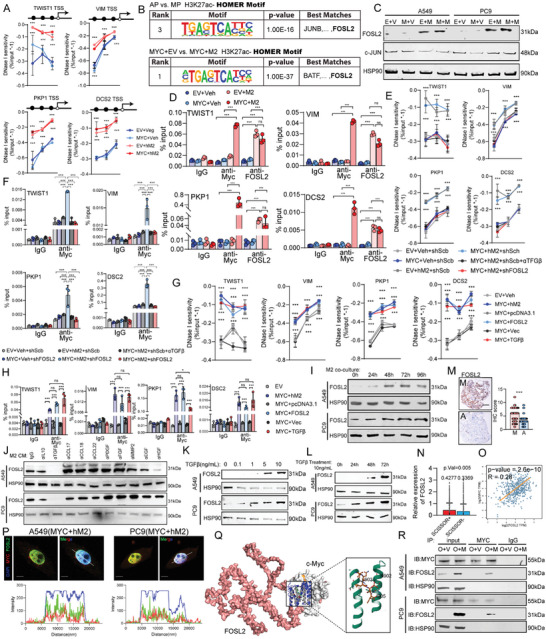
MYC in collaboration with M2‐like macrophages gains unique binding sites that exhibit alternative transcriptional regulatory activity. A) The DNase I hypersensitivity site identified via DNase I treated‐PCR in promoters of MP‐pattern genes in E‐V cells, M‐V cells, E‐M2 cells and M‐M2 cells (A549 cell based). B) HOMER de novo motif analysis on altered H3K27ac occupied sites in AC‐subtype tissue versus MP‐subtype tissue, and M‐V cells versus M‐M2 cells. C) Western Blots of FOSL2 and JUN in cells with different treatment. Three biological replicates were performed for each cell. D) ChIP‐PCR of IgG, MYC and FOSL2 for binding promoters of MP‐pattern genes in A549 cells with different treatment. E) The DNase I hypersensitivity site identified via DNase I treated‐PCR in promoters of MP‐pattern genes in A549 cells with different treatment. The dependence experiment revealed that the high DNase I sensitivity of MP‐pattern gene promoters induced by M2‐like macrophages depends on TGFβ‐FOSL2 axis. F) ChIP‐PCR of IgG, MYC and FOSL2 for binding promoters of MP‐pattern genes in A549 cells with different treatment. The dependence experiment revealed that MYC binding MP‐pattern gene promoters in M‐M2 A549 cells depends on TGFβ‐FOSL2 axis. G) The DNase I hypersensitivity site identified via DNase I treated‐PCR in promoters of MP‐pattern genes in A549 cells with different treatment. The rescue experiment revealed that the high DNase I sensitivity of MP‐pattern gene promoters in solely MYC‐overexpression A549 cells were rescued by FOSL2 overexpression or exogenous addition of TGFβ. H) ChIP‐PCR of IgG, MYC and FOSL2 for binding promoters of MP‐pattern genes in A549 cells with different treatment. The rescue experiment revealed that MYC binding MP‐pattern gene promoters in M‐V A549 cells were rescued by FOSL2 overexpression or exogenous addition of TGFβ. I) Western Blots of FOSL2 in cancer cells with different time‐point of coculture with M2‐like macrophages. Three biological replicates were performed for each cell. J) Western Blots of FOSL2 in cancer cells coculture with M2‐like macrophages and several antibodies were used to neutralize cytokines. Three biological replicates were performed for each cell. K,L) Western Blots of FOSL2 in cancer cells exogenous addition of TGFβ with different concentration (K) and treatment time (L). M) Representative images of immunohistochemistry of FOSL2 in tissues micro‐array from MAPes cohort. Right: quantitative statistics. Scale bars: 100 µm. N) Relative expression of FOSL2 in SCISSOR+ cells (MP‐subtype proneness, mean expression: 0.4277) and SCISSOR‐ cells (AC‐subtype proneness, mean expression: 0.3369) from single‐cell RNA‐seq data in Figure 3A. O) The correlation analysis in expression of MYC and expression of FOSL2 from TCGA‐LUAD mRNA dataset. P) Representative images of immunofluorescence of FOSL2 (green), MYC (red) and DAPI (blue) in M‐M2 cells, Scale bars: 1µm (Upper). The detected fluorescence intensity at the white line (Bottom). Q) Virtual docking was performed based on the protein structure of MYC and the tertiary structure of FOSL2 protein predicted by Alphafold2. R) Co‐immunoprecipitation of c‐Myc antibody and IgG antibody indicated that MYC binding FOSL2 after redundant expression when cancer cells were co‐cultured with M2‐like macrophages. For each group, n = 5. The p values were determined by Student's *t*‐test (unpaired two‐tailed), n.s., not significant; *p < 0.05, **p < 0.01, ***p < 0.001. Data are represented as mean ± SEM.

Increased chromatin accessibility can help recruit transcription factors to active regulatory elements thereby mediating downstream transcriptional regulation.^[^ ^50^ ^]^ DNase I‐treatment PCR was used to detect chromatin alterations in order to illustrate whether M2‐like macrophages facilitate MYC induced chromatin accessibility of MP‐pattern genes. The results showed that M2‐like macrophages significantly increased chromatin accessibility of MP‐pattern genes rather than canonical target genes of MYC, such as CDK6 and VEGFA etc. (**Figures** **6**A, and S9C,D, Supporting Information). Subsequently, we performed motif analysis on H3K27ac occupied sites from AC‐ and MP‐subtype tissues for seeking key transcriptional factor specifically increasing chromatin accessibility in MP‐tissue. Among the top ranked motifs, we found FOSL2, components of the AP‐1 transcription factor complex, which has also been reported to serve as pioneer transcription factors,^[^ ^51^ ^]^ was predicted to bind in differentially H3K27ac occupied sites(Figure 6B). Furthermore, compared to M‐V cells, M‐M2 cells exhibited a consistent enrichment of FOSL2 transcription factor in the adjacency of differentially H3K27ac occupied sites (Figure 6B). In addition, the TCGA‐LUAD ATAC‐seq data showed that patients with high FOSL2 expression had higher chromatin accessibility around loci of MP‐pattern genes (Figure S9E, Supporting Information). Previous studies have reported that FOSL2 can independently act as a pioneer transcription factor, enhancing local chromatin accessibility, regardless of the AP‐1 complex.^[^ ^51^ ^]^ Comparing to JUN, the key component of AP‐1 transcriptional complex, FOSL2 were elevated in LUAD cells co‐cultured with M2‐like macrophages regardless of MYC overexpression (Figure 6C).

Interestingly, FOSL2 preferentially bound to the promoters of target genes prior to MYC, even when tumor cells were co‐cultured solely with M2‐like macrophages (Figure 6D), which was not observed with the canonical target genes of MYC (Figure S6F,G, Supporting Information). Therefore, we hypothesized that cytokine secreted by M2‐like macrophages may contribute to the regulation of MP‐pattern genes by MYC and FOSL2. Dependence experiments using shRNA against FOSL2 demonstrated that the local accessibility of promoters of MP‐pattern genes and MYC binding chromatin in M‐M2 cells depended on the pioneering binding of FOSL2 to expose nucleosomes (Figures 6E,F, and S9H, Figure S10, Supporting Information). Rescue experiments using FOSL2 overexpression plasmids demonstrated that local opening of MP‐pattern gene promoters and MYC binding chromatin in M‐V cells was recovered upon excessive expression of FOSL2 (Figures 6G,H, and S9H, Figure S11, Supporting Information). Those phenomena were not observed in the canonical target genes of MYC. Taken together, these results suggest that M2‐like macrophage‐associated FOSL2 may facilitate MYC binding to the promoters of MP‐pattern genes rather than the canonical target genes of MYC by functioning as a pioneering transcription factor.

To elucidate the mechanism by which M2‐like macrophages promote elevated expression of FOSL2 in tumor cells (Figures 6I, S12A, Supporting Information), we employed neutralizing antibodies against various M2‐like macrophage‐associated cytokines and growth factors in our co‐culture system. The results demonstrated a significant inhibition of FOSL2 expression in tumor cells upon addition of a neutralizing antibody against TGFβ (Figures 6J, and S12B, Supporting Information). The exogenous addition of recombinant human TGFβ effectively promoted the expression of FOSL2 in tumor cells (Figure 6K,L). Additionally, elevated FOSL2 expression in the subcutaneous tumor samples was consistently accompanied by increased TGFβR1 expression (Figure S12C,D, Supporting Information). Analysis of the TCGA‐LUAD mRNA database showed a significant positive correlation between FOSL2 and TGFB1 expression levels (Figure S12E, Supporting Information). This is consistent with previous reports where FOSL2 has been identified as a downstream mediator of TGFβ signaling in various tumors, including non‐small cell lung cancer.^[^ ^52^, ^53^ ^]^ To determine whether activation of the canonical TGFβ pathway is required for TGFβ‐associated MP‐pattern genes transcription, shRNA‐mediated knockdown of SMAD2, SMAD3 and SMAD4, which reported in our previous study,^[^ ^45^ ^]^ was performed in M‐M2 tumor cell. The results revealed that knockdown of any of the three proteins individually failed to reverse the transcription of MP‐pattern genes in M‐M2 tumor cells (Figure S12F, Supporting Information). Functionally, neutralizing TGFβ antibody inhibited the chromatin accessibility and MYC binding at promoters of MP‐pattern genes in M‐M2 cells (Figures 6E,F, and S10, Supporting Information). While exogenous addition of TGFβ effectively increased the chromatin accessibility and MYC binding at promoters of MP‐pattern genes in M‐V cells (Figures 6G,H, and S11, Supporting Information). Those phenomena were not observed in the canonical target genes of MYC. These results indicated that M2‐like macrophages promoted FOSL2 expression in tumor cells through secreting TGFβ.

We further investigated whether FOSL2 accumulates in MP‐subtype LUAD tissue. The MAPes cohort TMAs revealed a significant upregulation of FOSL2 expression in the MP‐subtype cores compared to the AC‐subtype cores (Figure 6M). The single‐cell RNA‐sequencing data in Figure 3A similarly demonstrated that SCISSOR+ cells (MP‐pattern) exhibited more pronounced FOSL2 expression (Figure 6N). Furthermore, TCGA‐LUAD mRNA data revealed a significant positive correlation between MYC and FOSL2 expression (Figure 6O). However, this significant correlation was not evident in LUAD cell lines from CCLE dataset (Figure S12F, Supporting Information). Reinforcing analysis of Figure 2A and Figure S3E, Supporting Information in subcutaneous tumor tissues of C57BL/6J mice exhibiting MP‐pattern malignancy switching due to MYC overexpression revealed a significant increase in FOSL2 expression. Conversely, in tumors derived from BALB/c Nude mice without MP‐pattern malignancy switching, FOSL2 exhibited no significant changes (Figure S12G, Supporting Information). The consistent findings of FOSL2 expression were also observed in the subcutaneous tumor tissues as depicted in Figure 4A (Figure S12H, Supporting Information). Those consistent with previous reports of redundant FOSL2 expression being associated with highly metastatic tumors.^[^ ^54^ ^]^

Spatially, we observed co‐localization of MYC and FOSL2 in the nucleus of M‐M2 tumor cells (Figure 6P, Figure S12I, Supporting Information). Computational docking analysis also indicated the potential binding between MYC and FOSL2 (Figure 6Q). Co‐immunoprecipitation (co‐IP) experiments demonstrated physical bonding between MYC and FOSL2 in M‐M2 tumor cells (Figure 6R, Figure S12J, Supporting Information). Based on the computational docking predictions, we designed full‐length MYC plasmids as well as truncated MYC plasmids and transfected them into MYC knockout cell lines, respectively. The results demonstrated that under TGFβ‐treated conditions, the full‐length MYC protein effectively bound to FOSL2, while the truncated MYC protein failed to bind (Figure S12K,L, Supporting Information). These results suggest that FOSL2 can bind near the C‐terminus of the MYC protein, assisting transcriptional regulatory functions of MYC.

Transcription initiation and activation requires precise coordination and mutual promotion between chromatin accessibility and histone acetylation signaling. Therefore, we further investigated the regulatory roles of histone acetyltransferases (HATs) and deacetylases (HDACs) on MP‐pattern genes.^[^ ^55^ ^]^ First, in the MAPes patient TMA and cell samples, we did not observe any significant changes in the protein expression levels of key histone acetyltransferases (ac‐p300/CBP) and deacetylases (including Class I HDACs, HDAC1, HDAC2 and HDAC3) (Figure S13A,B, Supporting Information). To investigate the functional roles of HATs and HDACs in the transcriptional regulation of MP‐pattern genes, ChIP‐PCR assays showed that the promoters of MP‐pattern genes in M‐M2 group were all enriched for ac‐p300/CBP binding. In addition, reduced binding of HDACs to the promoters of MP‐pattern genes was also observed in M‐M2 group, among which the change of HDAC1 binding was the most significant (Figure S13C, Supporting Information). We performed ac‐p300/CBP and HDAC1 ChIP‐seq in MP and AC‐subtypes tissue separately to gain a comprehensive view of their regulatory roles on MP‐pattern genes. The results showed that genes undergoing transcriptional activation in MP‐subtypes had more loss of ac‐p300/CBP and HDAC1 binding around them, while genes subjected to transcriptional repression in MP‐subtypes showed the opposite pattern (Figure S13D,E, Supporting Information). These results indicate that HATs and HDACs are widely involved in the transcriptional regulation of MP‐pattern genes. We further investigated whether HATs facilitated chromatin accessibility mediated by FOSL2. At the expression level, using the p300 inhibitor C646 significantly reversed the transcription increase of MP‐pattern genes mediated by M‐M2 (Figure S13F, Supporting Information). Concurrently, C646 also broadly suppressed chromatin accessibility at the promoters of MP‐pattern genes mediated by M‐M2 (Figure S13G, Supporting Information). The above results indicate that chromatin accessibility at the promoters of MP‐pattern genes mediated by FOSL2 involves and is partially dependent on the assistance of HATs.

### 2.6 Inhibition of the TGFβ‐FOSL2 Axis Effectively Diminishes the Malignancy Associated with the MP‐Pattern

**Figure 7:**
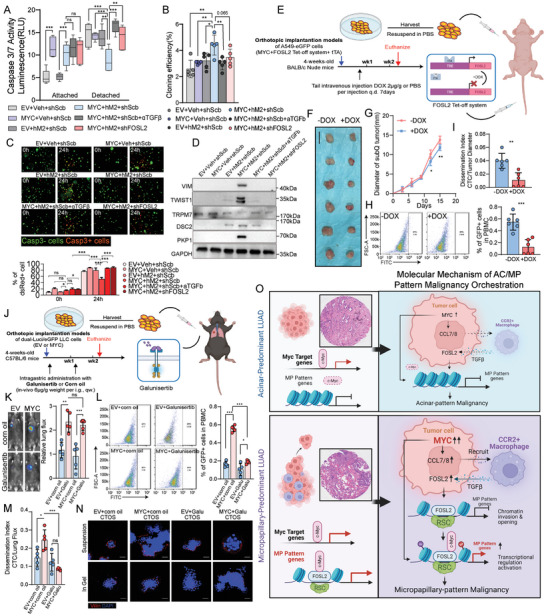
Inhibition of the TGFβ‐FOSL2 axis effectively diminishes the malignancy associated with the MP‐pattern. A–D) The dependence experiment revealed that detachment‐induced cell death resistance (A), non‐anchored clonality ability (B), anti‐shearing force (C) and MP‐pattern genes expression in protein level (D) induced by M2‐like macrophages depends on TGFβ‐FOSL2 axis in cancer cell lines in A549 cells. For detachment‐induced cell death assay, Lung cancer cell lines were cultured attached or detached on TC‐treated plates or covalently bound hydrogel layer‐treaded plates. For non‐anchored clonality ability, indicated cells were allowed to grow in soft agar for 2 weeks and colonies were counted. E) An experimental illustration showing the rescue experiment in BALB/c Nude athymic mice with A549 cells xenograft, which transfected with FOSL2 tet‐off system. F,G) Tumor images (F) and growth kinetics (G) of subcutaneously implanted tumors in 2‐week‐after‐implanted FOSL2 tet‐off system transfected A549 cells and treated/not treated with doxycycline. n = 6. Scale bars: 1 cm. H) CTCs detected from venous blood in BALB/c Nude athymic mice 2‐week‐after‐implanted FOSL2 tet‐off system transfected A549 cells, which treated/not treated with doxycycline. Left: Representative images, right: quantitative statistics. n = 6. I) Dissemination index detected from venous blood in BALB/c Nude athymic mice bearing 2‐week‐after‐implanted FOSL2 tet‐off system transfected A549 cells, which treated/not treated with doxycycline. n = 6. J) An experimental illustration showing efficacy of Galunisertib against the malignancy of MP‐pattern with redundant expression of MYC via orthotopic lung injection. n = 5. K) Lung tumor growth was detected using in vivo imaging. Left: Representative images, right: quantitative statistics. n = 5. L,M) CTCs (L) and Dissemination index (M) detected from venous blood in BALB/c Nude athymic mice bearing MYC basal/redundant expression tumor, treated or not treated with Galunisertib. n = 5. N) Representative images of immunofluorescence staining of CTOS derived from indicated tumor tissue. Red, villin; blue, DAPI. Scale bars: 20 µm. O) Working model for MP/AC‐pattern malignancy orchestration. The p values were determined by Student's *t*‐test (unpaired two‐tailed), n.s., not significant; *p < 0.05, **p < 0.01, ***p < 0.001. Data are represented as mean ± SEM.

In order to further validate the association of the TGFβ‐FOSL2 axis in MP‐patterned malignancy, we first knocked down FOSL2 or inhibited TGFβ in lung cancer cells co‐cultured with M2‐like macrophages. The results demonstrated that blocking both FOSL2 and/or TGFβ effectively reversed processes associated with MP‐patterned malignancy such as resistance to anoikis and shear stress, and meanwhile promoting the expression of markers associated with EMT and resistant to circumferential shear forces (**Figure** **7**A–D, Figure S14A–C, Supporting Information). In an in vivo model, knockdown of FOSL2 in MYC‐overexpressing mouse LLC cells prior to establishing the C57BL/6J xenograft model did not alter subcutaneous tumor growth rate but greatly reduced CTC numbers and dissemination index (Figure S14D–I, Supporting Information). To further illustrate impacts of FOSL2 on MP malignancy in vivo, a Tet‐Off system was employed to block FOSL2 of MYC‐overexpressing A549 cells, followed by the establishment of a BALB/c nude mice xenograft model (Figure 7E, Figure S14J,K, Supporting Information). We observed that tumor growth rate was not attenuated by FOSL2 absence, while the quantity of CTCs and the dissemination index in peripheral blood were significantly decreased (Figure 7F–I, Figure S14L, Supporting Information).

We also undertook to determine whether the association of the TGFβ/FOSL2 axis with MP‐patterned malignancy could be severed by pharmacologically inhibitor. Galunisertib, an inhibitor of the TGFβ receptor I kinase activity, was selected since which had advanced to phase II clinical trials in solid tumors including colorectal, pancreatic, liver and non‐small cell lung cancers for its safety and efficacy (NCT03470350, NCT02734160, NCT02423343, NCT01373164, NCT03206177). A lung cancer orthotopic model was established in C57BL/6J mice and treated with Galunisertib by gavage (Figure 7J). The findings indicated that Galunisertib exhibited no significant inhibitory effect on the growth of primary tumors, regardless of the presence or absence of MYC overexpression (Figure 7K). Nonetheless, the treatment model harboring MYC‐overexpressing tumor revealed that not only was there a significant decrease in local M2‐like macrophages infiltration, but there was also a notable reduction in the detection rate of CTCs and the dissemination index in the peripheral blood of mice (Figure 7L,M, Figure S14M,N, Supporting Information). In addition, the CTOSs extracted from the aforementioned lung orthotopic tumors exhibited apical membrane polarity switching characteristics in the MYC overexpression derived CTOSs, which were reversed by Galunisertib (Figure 7N, Figure S14O, Supporting Information). These results suggest that interference of the M2‐like macrophage‐TGFβ‐FOSL2 axis can reverse MP‐pattern malignancy in harboring redundant MYC expression LUAD.

## 3 Discussion

Our study identified molecular features of epigenetic and transcriptional programs underlying MP‐subtype LUAD, demonstrating that the excessive expression of MYC in cooperation with the pioneer transcription factor FOSL2, induced by TGFβ secreted by M2‐like macrophage, leads to increased accession to novel loci at promoters of MP‐pattern genes, thereby facilitating MYC binding. The consociation between MYC and M2‐like macrophages contributes to the development of MP‐patterned malignancy. These results are consistent with a previous study demonstrating that widespread chromatin accessibility to regulatory elements at the TGFβ promoter.^[^ ^56^ ^]^ Although we focused on FOSL2, additional studies are needed to gain a deeper understanding of interactions between FOSL2 and MYC, such as genome‐wide chromatin accessibility analysis, considering the data indicating that FOSL2 functions as a pioneer factor at de novo regulatory elements in human embryonic stem cells.^[^ ^51^ ^]^

Although developing the typical morphology of MP carcinoma in in vivo studies is rare, it has been demonstrated that 3D culture in vitro can indeed induce unique cellular polarity consistent with the features of MP carcinoma.^[^ ^13^ ^]^ In addition, the molecular features of MP carcinoma have been extensively discovered in recent years through numerous pathological studies, including but not limited to EMT and anti‐apoptotic mechanisms.^[^ ^10^, ^23^, ^24^ ^]^ Consistent alterations of these molecular features were observed in both previous analysis of RNA‐seq data of LUAD pathological subtypes^[^ ^32^ ^]^ and our micro‐dissected RNA‐seq data. These findings suggest the existence of a core transcriptional program driving these processes under multiple molecular features of the MP‐subtype LUAD. Our data found a significantly activated oncogenic MYC pathway in MP‐subtype LUAD. Moreover, this activation resulted from both the excessive expression due to MYC copy number amplification, and the acquisition of novel MYC binding sites facilitated by FOSL2. These MP‐pattern genes transcriptionally regulated by MYC encompassed key regulators of EMT, cell adhesion and anti‐shear force, which were under distinct transcriptional regulation programs compared to canonical MYC‐targeted genes and competed for MYC binding. Our study also revealed interesting findings. For example, when c‐Myc was expressed at low levels, it preferentially bound to canonical MYC‐target genes, including cell cycle regulatory factors. In contrast, for non‐classical MYC‐target genes such as CCL7 and CCL8, c‐Myc binding to their promoter regions was observed only under conditions of c‐Myc redundancy. This heterogeneity in chromatin binding may be attributed to differences in the binding motif flanking sequences or variations in chromatin topology.^[^ ^57^, ^58^, ^59^ ^]^ This part explains the reason why the CCL7/8‐M2‐like macrophage‐FOSL2 axis was not triggered in low/mid‐risk pattern LUAD. Recent genomic studies on LUAD histological subtypes also indicated that the high‐risk patterns, including MP‐subtype LUAD, had the highest number of copy number amplifications and MYC pathway activations.^[^ ^14^ ^]^ Our results provide a compelling explanation for the association between redundant MYC expression and MP‐subtype LUAD.

Our data support that in MP‐subtype LUAD, recruited M2‐like macrophages stimulate tumor cell FOSL2 expression through secreting TGFβ, thereby increasing accessibility to novel MYC chromatin binding sites. Macrophages, as highly plastic cells derived from the myeloid lineage,^[^ ^60^ ^]^ can assist tumor cells in evading immune surveillance and promoting progression through various mechanisms, including the secretion of cytokines such as TGFβ, complement clearance, and extracellular matrix remodeling.^[^ ^61^, ^62^ ^]^ Maddipati et al. found in their study of pancreatic ductal carcinoma that MYC can promote tumor cell metastasis by recruiting macrophages.^[^ ^47^ ^]^ Additionally, previous data have also shown that TGFβ can exert transcriptional regulatory functions by increasing chromatin accessibility of a vast number of regulatory elements, and these regions are enriched for footprints of AP‐1 transcription factor complexes.^[^ ^56^ ^]^ Our data support the involvement of the pioneering transcription factor FOSL2, as a core component of AP‐1 complexes, but we did not observe upregulation of JUN family proteins.^[^ ^63^ ^]^ There is a mutual promotional relationship between chromatin accessibility and histone acetylation.^[^ ^55^ ^]^ which cooperate to remodel chromatin and regulate downstream transcription. On the one hand, accessible chromatin provides a structural foundation for more transcription factors with acetylase recruitment functions to bind. For example, p53 manipulated by chromatin accessibility^[^ ^64^, ^65^ ^]^ can first bind to the promoters of cell cycle‐related genes and recruit acetylases p300/CBP to mediate transcriptional regulation.^[^ ^66^ ^]^ Our study found that FOSL2 the involvement of invading and opening local chromatin prior to c‐Myc, which allowed redundantly expressed c‐Myc to exert greater transcriptional regulatory functions. In the above molecular mechanisms, HATs may be involved in chromatin accessibility enhancement mediated by FOSL2. Our results led us to an interesting speculation: the opened chromatin by FOSL2 recruited HATs to mediate histone acetylation in a cascading reaction, significantly enhancing the local chromatin accessibility at the promoters of MP‐pattern genes. The stable binding of FOSL2 and c‐Myc also suggests that this kind of “cooperative” relationship may widely exist in chromatin accessibility regulation. Genome‐wide co‐occupied binding sites need to be identified to discover more drug targets for MP carcinoma. Furthermore, the roles of chromatin remodeling factors such as the SWI/SNF complex in altering MP‐pattern genes loci chromatin accessibility remain uncovered, which could be another potential therapeutic target.^[^ ^67^ ^]^

We observed excessively expressed MYC in MP‐subtype LUAD, but unfortunately, due to its crucial roles in normal physiological functions, MYC itself is difficult to use as a drug target.^[^ ^68^ ^]^ Given that the core function of MYC is transcription, which involves complex MYC protein‐protein interactions,^[^ ^34^ ^]^ hijacking the normal MYC pathway may promote tumor progression. Interfering with molecular partners of MYC to prevent its ability to regulate transcriptional programs is an attractive therapeutic strategy.^[^ ^69^ ^]^ Current inhibitory strategies targeting M2‐like macrophages mainly including reducing or eliminating tumor‐associated macrophages (TAMs), re‐inducing them into M1‐like macrophages, and promoting the phagocytosis and antigen presentation of TAMs by blocking “don't eat me” signals.^[^ ^70^ ^]^ In our study, we found that Bindarit, a pharmacological inhibitor of CCL7/CCL8, could effectively inhibit tumor cell recruitment of M2‐like macrophages in vitro. Bindarit has also been used previously in clinical trials for the prevention of in‐stent restenosis of coronary arteries and the amelioration of acute proliferative lupus nephritis with proteinuria.^[^ ^71^, ^72^ ^]^ However, in the above studies, patients showed good tolerance to Bindarit but there were no statistically significant differences in primary clinical endpoints compared to controls. This may be because CCL2/7/8‐mediated chemotaxis of monocytes and other myeloid lineage cells does not rely on a single pathway alone, and also involves multiple pathways such as the CCL5‐CCR1 axis. Further studies are needed to evaluate the distribution of CCL7/8 in MP‐subtype LUAD patients, which could be critical for determining whether Bindarit can effectively inhibit the recruitment of M2‐like macrophages in MP‐subtype LUAD. In recent years, studies inhibiting immune checkpoint molecules on the surface of M2‐like macrophages to reactivate phagocytic signals have achieved good effects in multiple preclinical studies of solid tumors. In studies of ovarian cancer and breast cancer, blocking the CD24‐Siglec10 immune checkpoint axis can effectively enhance the phagocytic function of macrophages against tumors.^[^ ^73^, ^74^ ^]^ In addition, CD47 is a widely expressed inhibitory signal on cancer cells that binds to SIRPα on the surface of macrophages to suppress phagocytic function through this interaction.^[^ ^74^, ^75^ ^]^ Therefore, blocking the CD47‐SIRPα axis with anti‐CD47 monoclonal antibodies or extracellular vesicles has been shown to effectively inhibit the malignant progression of various cancers including T‐cell lymphoma,^[^ ^76^ ^]^ acute myeloid leukemia,^[^ ^75^ ^]^ bladder cancer,^[^ ^77^ ^]^ B‐cell non‐Hodgkin's lymphoma,^[^ ^78^ ^]^ and pediatric malignant brain tumors.^[^ ^79^ ^]^ Thus, inhibiting the function of M2‐like macrophages through immune checkpoint inhibitors represents a promising therapeutic strategy. The TGFβ receptor I inhibitor Galunisertib selected in our study can effectively suppress the MP‐pattern malignancy triggered by the cascading M2‐like macrophage‐TGFβ‐FOSL2 axis induced by the redundant expression of MYC.^[^ ^80^ ^]^ Galunisertib has undergone Phase II clinical trials in numerous solid tumors (NCT03470350, NCT02734160, NCT02423343, NCT01373164, NCT03206177), and has been shown to be a reliable, safe and promising new targeted drug with broad application prospects.

Based on our findings, it remains a challenge to uncover whether TGFβ secreted by M2‐like macrophages is the sole mediator of MP‐pattern malignant characteristics, and whether other factors in the microenvironment also interfere with the formation of MP‐pattern malignant phenotypes. A comprehensive investigation based on *Adgre1‐creER; Mrc1‐dreER* lineage tracing genetically engineered mouse models, which can specifically label TGFβ from M2‐like macrophage origins through the loxP‐Stop‐loxP ‐fluorescent protein system, would be appropriate for future studies to fully understand the underlying intricacies.

## 4 Experimental Section

### Human Samples

The retrospective cohort study MAPes (**M** icropapillary **A** denocarcinoma **P** athology R **e** tro **s** pective) included 66 patients who underwent curative resection for pulmonary adenocarcinoma harboring a micropapillary component at the Department of Thoracic Surgery, Jiangsu Province Hospital between October 2019 and June 2020. Histopathological examination conducted by experienced pulmonary pathologists confirmed the diagnosis of pulmonary adenocarcinoma with a predominant micropapillary phenotype according to established criteria. Furthermore, this cohort of 66 patients were followed up for 2 years post‐resection through medical record reviews and radiological investigations to longitudinally assess clinical outcomes such as locoregional recurrence rates and distant metastatic progression over the defined follow‐up period. Clinical, histopathological and prognostic characteristics of the patients are summarized in Table S1, Supporting Information. Patient demographics, tumor‐node‐metastasis (TNM) staging classification according to the American Joint Committee on Cancer (AJCC) criteria, tumor differentiation grades as determined by experienced pulmonary pathologists, as well as postoperative outcomes including recurrence patterns and survival data were retrospectively collated from medical records and radiological investigations for statistical analysis and comparison between subgroups stratified according to predefined clinicopathological variables.

Tissue microarrays were constructed in 66 patients from MAPs cohort, based on histopathological examination of H&E‐stained slides by experienced pulmonary pathologists. Triplicate cores were extracted from formalin‐fixed paraffin‐embedded blocks of normal lung parenchyma, non‐micropapillary pulmonary adenocarcinoma subtypes (including 6 lepidic, 21 papillary, 4 solid and 35 acinar predominant cases), as well as the micropapillary pulmonary adenocarcinoma regions from each patient. In order to perform Taqman probe‐based detection of MYC gene copy number in acinar and MP tissues, we selected 35 tissue samples from the MAPs cohort that contained AP tissue. These tissues were subjected to paired microdissection and subsequent extraction of DNA from both AP and MP tissues. Furthermore, three pairs of tissues were randomly selected from the aforementioned samples and subjected to next‐generation high‐throughput sequencing. Finally, we randomly selected three additional tissue specimens from the aforementioned tissues, and extracted DNA using a 1mm tissue microdissection punch handle. In situ MYC copy number detection was performed using Taqman probes. The study protocol was approved by the ethics committee of Nanjing Medical University (NJMU‐2022‐265). All patients had signed informed consent for donating their samples.

### Mice

C57BL/6JGpt (Strain No.: N000013) female mice at 4 weeks old were purchased from Gempharmatech Co., Ltd and maintained at Animal Core Facility of Nanjing Medical University according to protocols approved by the Institutional Animal Care and Use Committee of Nanjing Medical University (IACUC‐2209055). All experiments were conducted in mice following 1 week of acclimatization. For subcutaneous tumor transplantation model, single‐cell suspensions of 5 × 10^5^ eGFP labeled‐tumor cells in 50µL of diluted Matrigel (1:1, Corning) were injected subcutaneously into the dorsal flanks of 5‐week‐old mice under anesthesia by 1.25% Tribromoethanol. Tumor growth was monitored every 3 days using calipers. For lung orthotopic tumor transplantation model, single‐cell suspense ions of 1 × 10^5^ eGFP/Luciferase dual‐labeled‐tumor cells in 10µL of diluted Matrigel (1:1, Corning) injected directly into the left lung of mice using a 32G needle under anesthesia by 1.25% Tribromoethanol. Tumor growth was monitored every 5 days using IVIS Spectrum In Vivo Imaging System. For all mice that reached the experimental endpoint, mice were subjected to deep anesthesia, followed by blood collection from the orbital sinus and subsequent euthanasia. Peripheral blood obtained from the orbital sinus of mice was processed using ACK Lysis Buffer (KeyGEN BioTech) to extract peripheral blood mononuclear cells (PBMCs) for subsequent experiments. Subcutaneous tumor tissues or lung tissues were dissected and weighed. Subsequently, the tumor tissues were fixed in formalin and embedded in paraffin for further experimental analysis. For tumor samples cultured as CTOSs, fresh samples were dissected into small pieces using sterile scissors and added to 1 mL of tissue dissociation solution (absin, # abs9482) for incubation at 37 °C with rotation for 45 min. The resulting suspension was passed through a 70 µm cell strainer, and the flow‐through was collected for CTOSs culture.

For certain experiments, the following commercial reagents were used to treat the mice. To deplete macrophages in vivo, anti‐F4/80 neutralizing antibody (Bio X Cell, RR_ID: AB_10949019) or IgG2b control antibody (Bio X Cell, RR_ID: AB_2687733) was dissolved in pH 7.0 Dilution Buffer (Bio X Cell, #IP0070). Each mouse was intravenously injected with 100 µg of the respective antibody on days −3, +1, +5, +10, and +14, following the protocol described in reference.^[^ ^81^ ^]^ In addition, following the method described in reference,^[^ ^82^ ^]^ we used clodronate liposome (YEASEN, #40337ES08) or control liposome (YEASEN, #40338ES) to eliminate macrophages. Each mouse was intravenously injected with 50 µg g^−1^ body weight of clodronate liposome or control liposome on days −3, +1, +5, +10, and +14. To activate the FOSL2 Tet‐off system in mice, we administered doxycycline (Sigma‐Aldrich) via tail vein injection at a dosage of 2 µg g^−1^ body weight, once daily from day +1 to +7. To assess the inhibitory effect of Galunisertib on the malignancy of MP‐pattern LUAD, we referenced the dosing regimen from the Galunisertib clinical trial (ClinicalTrials.gov_ID: NCT03470350). Galunisertib (MedChemExpress, #HY‐13226) was dissolved in corn oil (Aladdin). Each mouse received an oral gavage of 6µg g^−1^ body weight of Galunisertib, three times a week for 1 week after transplantation.

### Zebrafish

AB line zebrafish adults at 3 months old were purchased from China Zebrafish Resource Center and maintained at Jiangsu Cancer Hospital. Zebrafish embryos were cultured in E3 medium and maintained at a constant temperature of 28 °C with a light‐dark cycle of 14:10. Single‐cell suspensions of ≈100 eGFP‐labeled tumor cells in PBS were perivitelline injected into 2‐day post‐fertilization (dpf) zebrafish larvae. Following tumor cell transplantation, zebrafish larvae were cultured in E3 medium at a constant temperature of 37 °C with a light‐dark cycle of 14:10. After 48 h post‐transplantation, the caudal region of zebrafish larvae was imaged using confocal microscopy, and the number of migrated cells was quantified. Zebrafish were housed and handled in accordance with approved protocols of Institutional Animal Care and Use Committee of Nanjing Medical University.

### Cell Culture

A549 human lung adenocarcinoma cell (CSTR:19375.09.3101HUMSCSP503), PC‐9 human lung adenocarcinoma cell (CSTR:19375.09.3101HUMSCSP5085), THP‐1 human monocyte (CSTR:19375.09.3101HUMSCSP567) and Lewis mice lung cancer cell (CSTR:19375.09.3101MOUTCM7) were purchased from Shanghai Cell Bank of Chinese Academy of Sciences. Those commercial cell lines and derivative cell lines (A549‐GFP, PC9‐GFP, LLC‐GFP, LLC‐dual‐Luci/GFP, M2‐like THP‐1 and M0‐like THP‐1) were maintained in DMEM (KeyGEN BioTech) supplemented with 10% Fetal Bovine Serum (Corning) and 1% penicillin‐streptomycin at 37 °C and 5% CO2. The majority of maintenance and experimental procedures were performed using suspension culture on polystyrene‐coated ultralow attachment surface plates or flasks (Corning). However, a few experiments that required adherent cell culture were conducted using TC‐coated plates or flasks.

The mouse‐derived macrophages were obtained from the peritoneal lavage fluid of C57BL/6J mice. After lysis of red blood cells using ACK Lysis Buffer (KeyGEN BioTech), the cells were seeded in culture bottles and maintained in DMEM (KeyGEN BioTech) supplemented with 10% Fetal Bovine Serum (Corning), 1% penicillin‐streptomycin, 500 ng mL^−1^ dexamethasone (Beyotime), 40 µg mL^−1^ insulin (Beyotime), and 20 ng mL^−1^ EGF (Novoprotein) at 37 °C with 5% CO2.

For the induction of M2‐like macrophages, we followed our previously established polarization method.^[^ ^45^ ^]^ Briefly, THP‐1 cells were treated with 40 ng mL^−1^ PMA (ThermoFisher) for 24 h to induce differentiation. Subsequently, the medium was replaced with culture medium supplemented with 40 ng mL^−1^ IL4 (Novoprotein) and 20 ng mL^−1^ IL13 (Novoprotein) for an additional 48 h of induction. A similar method was employed for the M2‐like polarization of mouse macrophages, with minor differences in the use of mouse‐derived IL4 and IL13 (Novoprotein) at the same doses.

For the co‐culture of M2‐like THP‐1 cells and human lung adenocarcinoma cells, as well as mouse M2‐like macrophages and Lewis lung cancer cells, a non‐contact co‐culture was performed using 3 µm Insert Systems (Corning) for 72 h. Prior to the xenograft experiments in zebrafish, direct contact co‐culture of the aforementioned cells was also carried out. For the macrophage chemotaxis assay, an 8 µm Insert System (Corning) was used for co‐culture to assess the migratory capacity of tumor cells toward M2‐like macrophages in the chamber.

For certain experiments, cells were treated with the following commercial reagents. To activate FOSL2 overexpression in tumor cells using the Tet‐on system and induce FOSL2 knockdown using the Tet‐off system, tumor cells were treated with 1 µg mL^−1^ doxycycline (Sigma‐Aldrich) under the aforementioned culture conditions. In some experiments, tumor cells were treated with recombinant TGFβ1 (2 ng mL^−1^, Novoprotein, #GMP‐CA59), p300 inhibitor C646 (400 nM, MedChemExpress, #HY‐13823) or CCR2 inhibitor INCB3344 (1 µM, MedChemExpress, #HY‐50674). Induced M2‐like THP‐1 cells were treated with anti‐TGFβ blocking antibody (6µg mL^−1^, R&D Systems, RR_ID: AB_357931), anti‐IL10 blocking antibody (6µg mL^−1^, R&D Systems, RR_ID: AB_354401), anti‐CCL17 blocking antibody (6µg mL^−1^, R&D Systems, RR_ID: AB_355325), anti‐CCL18 blocking antibody (6µg mL^−1^, R&D Systems, RR_ID: AB_355344), anti‐CCL22 blocking antibody (6µg mL^−1^, R&D Systems, RR_ID: AB_2072232), anti‐PDGF blocking antibody (6µg mL^−1^, R&D Systems, RR_ID: AB_354273), anti‐FGF blocking antibody (6µg mL^−1^, R&D Systems, RR_ID: AB_354413), anti‐MMP2 blocking antibody (6µg mL^−1^, R&D Systems, RR_ID: AB_355701), anti‐EGF blocking antibody (6µg mL^−1^, R&D Systems, RR_ID: AB_355232), anti‐HGF blocking antibody (6µg mL^−1^, R&D Systems, RR_ID: AB_2279754), IgG1 isotype control (6µg mL^−1^, R&D Systems, RR_ID:AB_357344) or CCL7/8 inhibitor Bindarit (5µM, MedChemExpress, #HY‐B0498).

### Cell Transfection and Establishment of Stably Transduced Cells

To track the transplanted tumor cells in mice, tumor cells (including A549, PC‐9, and LLC cells) were stably transfected with eGFP‐expression lentiviral vector (pLV8ltr‐UbC‐EGFP‐BSD‐CMV, Corues Biotechnology Co., Ltd) and/or luciferase‐expression lentiviral vector (pCAG‐luciferase, Addgene, #55764, RR_ID: Addgene_55764). The stable transfection method involved adding an appropriate amount of lentivirus (5 MIO for A549 and PC‐9 cells, 6 MIO for LLC cells) and Hexadimethrine bromide (8 µg mL^−1^, MedChemExpress, #HY112735) to the culture plate seeded with the cells, followed by 24 h of infection and subsequent replacement with regular growth medium for 48 h. Antibiotics (BSD, MedChemExpress, #HY‐103401, 10 µg mL^−1^; Geneticin, Boyotime, #ST081, 250 µg mL^−1^) were then added in an appropriate amount until no viable cells remained in the blank wells. Transfection efficiency was validated using fluorescence microscopy (Zeiss) and a visible light detector (Promega).

To generate MYC knockout tumor cells, the CRISPR‐Cas9 technology was employed to disrupt MYC expression. The backbone of sgMYC used pSpCas9 BB‐2A‐Puro (GenScript). Transient transfection of sgRNA‐Cas9 plasmid was performed via Lipofectamine 3000 (ThermoFisher). The infected cells were subject to Puromycin Dihydrochloride (1µg mL^−1^, Beyotime, #ST551) selection for 5 days after transfection. Individual puromycin resistant colonies were picked up manually via 36G needle and then expanded in 12‐well plates. Colonies carrying a deletion allele were checked by Western Blots.

For certain experiments, cells were transfected with a full‐length c‐Myc expression vector (pcDNA3‐cmyc, Addgene, #16011, RR_ID: Addgene_16011). pcDNA3 empty vector (Corues Biotechnology Co.), targeting FOSL2 short hairpin RNA and control scribble (Sangon Biotech), FOSL2‐expression vector (Addgene, #187907, RR_ID: Addgene_187907), pcDNA3.1 empty vector (Corues Biotechnology Co.), FOSL2 tet‐off system vector (pLV3rsv‐hPGK‐FOSL2‐Tetoff‐IRES‐Puro, Corues Biotechnology Co.), dual‐luciferase reporter system plasmid (Corues Biotechnology Co.), CASP3‐promoter‐dsRed tracing system plasmid (Corues Biotechnology Co.) and MYC‐truncated plasmid (Corues Biotechnology Co.). The aforementioned plasmids were transfected using the manufacturer's standard plasmid transfection method with

Lipofectamine 3000 (ThermoFisher).

To efficiently transduce CTOSs derived from LUAD patients, we referenced the hairpin targets of pRetrosuper Myc shRNA (Addgene, #15662) (GATGAGGAAGAAATCGATG) and cloned them into the pLKO.1 backbone for lentiviral packaging. The lentiviral packaging was commissioned to Corues Biotechnology Co. CTOSs were transduced with shMYC and vector control lentiviruses at an MOI of 5 for 24 h in the presence of 5µg mL^−1^ Polybrene (MCE, #HY‐112735) to achieve highly efficient transduction of CTOSs.

### Frozen Section Microdissection

In order to accurately determine the transcriptional differences between MP subtype and Acinar subtype, in our study, experienced pathologists in the field utilized a Laser Capture Microdissection system to perform microdissection on frozen sections from three cases in the MAPes cohort that included both MP and Acinar subtypes. The dissected samples were then preserved in Trizol (ThermoFisher, #15596026) to obtain sufficient tissue for RNA‐seq analysis. Ten 8 µm frozen sections were used for each patient.

### RNA‐seq and RNA‐seq Analysis

Total RNA was isolated using standard Trizol‐base Methods. RNA‐seq libraries were prepared and sequenced by Nanjing Jiangbei New Area biopharmaceutical public service Platform Co., Ltd. RNA‐seq reads were aligned using STAR (version 2.4.0) to the reference human genome (GRCh38.p13) with Ensembl annotation (gencode. v33). DESeq2 (v1.30.1) R package was used to analyze the differential expression. Other downstream analysis was conducted by ClusterProfiler (v4.8.1) R package.

### CUT&Tag, CUT&Tag‐seq, ChIP, ChIP‐PCR, ChIP‐seq and Relevant Analysis

CUT&Tag for capturing histone binding DNA fragments and DNA libraries preparing were using Hyperactive Universal CUT&Tag Assay Kit (Vazyme, #TD903‐01) and TruePrep Index Kit (Vazyme, #TD202) via manufacturer protocol. H3K27ac antibody (CST, #8173) and c‐Myc antibody (CST, #18583) were used in CUT&Tag or ChIP.^[^ ^23^ ^]^ For CUT&Tag‐seq, DNA prepared libraries were sequenced by Romics (Shanghai). ChIP using a proven protocol^[^ ^83^ ^]^ both in tissue and cell‐lines. H3K27ac antibody (CST, #8173), c‐Myc antibody (CST, #18583), FOSL2 antibody (Santa Cruz, #sc‐166102), acetyl‐CBP/p300 antibody (CST, #4771) and HDAC1 antibody (CST, #34589) were used in ChIP. For ChIP‐PCR, the quantitative detection method was the same as in our previous study.^[^ ^84^ ^]^ The PCR primers are shown in Table S4, Supporting Information. For ChIP‐seq, DNA libraries preparing, sequencing and upstream analysis were conducted by Romics (Shanghai). Both CUT&Tag‐seq and ChIP‐seq data were processed using MACS2 for peak calling, and differential peak analysis was performed using MAnorm. Other downstream analyses and heatmap generation were conducted using Deeptools. The extraction of genes within 1000 bp around peak centers was performed using ChIPpeakAnno. Pathway enrichment analysis was carried out using the ClusterProfiler (v4.8.1) R package. Intervene was utilized to identify the overlap of peaks. For transcription factors motif enrichment analysis, Homer^[^ ^85^ ^]^ were used. To investigate the transcriptional impact of MYC in MP‐subtype tissue, Binding and Expression Target Analysis (BETA)^[^ ^49^ ^]^ was used.

### Public High‐Throughput Data and Analysis

Bulk RNA‐seq from GEO dataset were downloaded from GSE210029 and GSE58772, subsequent analysis was similar to aforementioned RNA‐seq analysis. For public high‐throughput data from TCGA‐LUAD dataset, mRNA‐associated analyses were similar to aforementioned RNA‐seq analysis. Moreover, maftools (v2.16.0) R package was used for detecting of copy number variant. For H&E staining slide from TCGA‐LUAD dataset digital sectioning, the method and results of H&E staining interpretation were reported in our previous study.

For single cell RNA‐seq (scRNA‐seq), public data were downloaded from GSE111229, Seurat (v4) R package was used for scRNA‐seq data processing. Scissor R package^[^ ^39^ ^]^ were used for mapping the results of microdissected bulk RNA‐seq. CellCall R packages^[^ ^41^ ^]^ were used for exploring the cell‐cell communication between tumor cells and various defined immune cells. Other analyses, such as different expression, were similar to aforementioned RNA‐seq analysis.

### Copy Number Detection

All MYC copy number detection by laboratory techniques were using MYC copy number TaqMan probe (Thermofiser, # Hs02758348_cn) and TaqMan Copy Number Assay (Thermofisher, #4400291) via manufacturer protocol. CopyCaller (v2.1) were used for data processing. Tissue sampling methods are described above.

### Dual‐Luciferase Reporter System

The Dual‐luciferase reporter system was used as previously described in our previous study.^[^ ^84^ ^]^ In brief, cells transfected with the firefly luciferase reporter gene plasmid were co‐transfected with Renilla luciferase plasmid (pGMLR‐TK, Corues Biotechnology Co.). The transcriptional activity of the promoter in the plasmid was detected using the Duo‐Lite Luciferase Assay System (Vazyme, #DD1205) according to the manufacturer's protocol.

### Co‐Immunoprecipitation

Co‐immunoprecipitations were conducted by Pierce Classic Magnetic IP/Co‐IP Kit (ThermoFisher, #88804). The procedures were performed according to the manufacturer's protocol.

### Anoikis Assay

Anoikis assays were conducted following proven methods^[^ ^25^, ^86^ ^]^with slight optimizing. Cell apoptosis was determined by using Caspase‐Glo 3/7 Assay (Promega, #G8981) in cell cultured within TC‐tread plate (Attached) or ultra‐low attachment plate (Detached), and Annexin V‐FITC/PI Apoptosis Detection Kit (KeyGen, #KGA108) in cell cultured within TC‐tread plate. For soft agar clone formation, ≈1000 cells were resuspended in DMEM containing 10% FBS with 0.7% agarose and layered on top of 1.2% agarose in DMEM on 12‐well plate. Cell culture conditions are the same as above. Colonies were counted using light microscopy.

### Anti‐Shearing Force Assay

Anti‐shearing force assay were conducted by CASP3‐promoter‐dsRed system and 10 cm s^−1^ circulatory culture system which modifications to the previous model.^[^ ^87^, ^88^ ^]^ Briefly, after transfected CASP3‐promoter‐dsRed system, GFP+ cancer cells suspension was flow into the chamber, which remolded by ultra‐low attachment flask, at a speed of 10 cm s^−1^, which closely mimics the real blood flow in human lung veins and maintained for 24 h. Due to the difficulty of maintaining tumor cells in a single‐cell format for an extended period within a flow chamber, the response of cells to shearing force was assessed by monitoring the dsRed fluorescence. The green/red‐fluorescent images of the attached cells were taken by fluorescence microscopy (Carl Zeiss), and at least five images were used for quantification.

### Immunostaining

We employed the standard immunohistochemistry (IHC) protocol of the Department of Pathology, Jiangsu Cancer Hospital. The following primary antibodies were used: E‐cad antibody (Proteintech, #20874‐1‐AP), TWIST1 antibody (Proteintech, #25465‐1‐AP), ITGB1 antibody (Proteintech, #12594‐1‐AP), Plakophilin 1 antibody (Proteintech, 22632‐1‐AP), TRPM7 antibody (Proteintech, #55251‐1‐AP), c‐Myc antibody (CST, #18583), VIM antibody (Proteintech, #10366‐1‐AP), TTF1 antibody (Proteintech, #66034‐1‐Ig), CD68 antibody (Proteintech, #28058‐1‐AP), ZEB1 antibody (CST, #70512), FOSL2 (Santa Cruz, #sc‐166102), Ki‐67 antibody (Proteintech, #27309‐1‐AP), CD3 antibody (Proteintech, #17617‐1‐AP), CD19 antibody (Proteintech, #66298‐1‐Ig), CD56 antibody (Proteintech, #14255‐1‐AP), CD1a antibody (Proteintech, #17325‐1‐AP), FcepsilonRI α antibody (Milliporesigma, #05‐491), CD123 antibody (Proteintech, #13655‐1‐AP), CD66b antibody (Bio‐Rad, #MCA216), Arg1 antibody (Proteintech, #66129‐1‐Ig), TGFβR1 antibody(Affinity, #AF5347), acetyl‐CBP/p300 antibody (CST, #4771), HDAC1 antibody (CST, #34589), HDAC2 antibody (CST, #57156) and HDAC3 antibody (CST, #85057). Experienced pathologists assessed the immunohistochemistry (IHC) results and assigned an IHC score (on a 12‐point scale) based on the evaluation of at least five fields of view.

For immunofluorescence, we utilized our established experimental protocol. The following primary antibodies were used: Max antibody (CST, #4739), Villin‐1 antibody (CST, #55883), CD68 antibody (Proteintech, #28058‐1‐AP), CD206 antibody (Proteintech, #60143‐1‐Ig), MHC‐II antibody (Santa Cruz, #sc‐13556), CD163 antibody (Proteintech, #16646‐1‐AP), CSF1R antibody (Proteintech, #25949‐1‐AP), c‐Myc antibody (CST, #18583) and FOSL2 antibody (Santa Cruz, #sc‐166102). The following secondary antibodies were used: anti‐Rabbit Alexa Fluor 488 (ThermoFisher, #A‐11034) and anti‐Mouse Alexa Fluor 555 (ThermoFisher, #A‐21422). For CTOSs polarity status staining, the preparation of the immunofluorescence (IF) samples followed the methods described in the study by Hiroaki et al.^[^ ^89^ ^]^

### CTOS Construction, Culturing and Observation of Polarity Status

Collecting tumor cells derived from LUAD samples according to Kondo et al. protocol^[^ ^31^ ^]^ with slight modification. The serum‐containing medium was DMEM supplemented with 10% B27. For cell lines, CTOS‐like culturing was also employed. Based on the polarity status of CTOSs, we classified CTOSs into “apical‐out” (apical proteins located exclusively at the outermost membrane of a CTOS) and “apical‐in” (including apical proteins located exclusively inside the CTOS and those located at both the outermost and inside of CTOS).

### DNase I Chromatin Accessibility Analysis

The method referred to the protocol by Brook S. et al. with slight modifications.^[^ ^90^ ^]^ Briefly, lysed chromatin samples were treated with 3 µl of DNase I (Vazyme, #EN401) for 5 min to achieve acceptable partial chromatin digestion. After terminating the digestion, chromatin fragmentation was performed using Covaris M220. Finally, qPCR detection was carried out using FAM‐labeled probes. The sequences of the probes and primers can be found in Table S4, Supporting Information.

### RNA Extraction, Reverse Transcription and qRT‐PCR

RNA extraction was performed using the classical Trizol method, followed by reverse transcription using the PrimeScript RT Master Mix (TAKARA, #RR036A). For qRT‐PCR, ChamQ SYBR qPCR Master Mix (Vazyme, #Q331) was utilized. The primer sequences for PCR can be found in Table S4, Supporting Information.

### Protein Extraction and Western Blotting

Pelleted cells were lysed by RIPA buffer supplemented with proteinase/phosphatase inhibitor cocktail (CST, #5872S). Protein concentration was quantified by BCA Protein Assay (Beyotime, #P0009). Then 15µg protein was loaded to Tris‐Gly gel. The following primary antibodies were used: ITGB1 antibody (Proteintech, #12594‐1‐AP), E‐cad antibody (Proteintech, #20874‐1‐AP), N‐cad antibody (Proteintech, #66219‐1‐Ig), TWIST1 antibody (Proteintech, #25465‐1‐AP), Plakophilin 1 antibody (Proteintech, 22632‐1‐AP), DSC2 antibody (Proteintech, #13876‐1‐AP), TRPM7 antibody (Proteintech, #55251‐1‐AP), c‐Myc antibody (CST, #18583), FOSL2 (CST, #19967), JUN antibody (Proteintech, #66313‐1‐Ig), HSP90 antibody (Proteintech, #13171‐1‐AP), GAPDH antibody (Proteintech, #60004‐1‐Ig). The following secondary antibody were used: Rabbit IgG Antibody Dylight 800 conjugated (Rockland, #611‐145‐002), Mouse IgG Antibody Dylight 800 conjugated (Rockland, #610‐145‐002), Rabbit IgG Antibody Dylight 680 conjugated (Rockland, #611‐144‐002), Mouse IgG Antibody Dylight 680 conjugated (Rockland, #610‐144‐002), HRP‐conjugated Anti‐Mouse IgG (Proteintech, # SA00001‐1) and HRP‐conjugated Anti‐Rabbit IgG (Proteintech, # SA00001‐2).

### Statistical Analysis

Sample size for each experimental group/condition is reported in the appropriate figure legends. All data points are presented for quantitative data, with an overlay of the mean with ± SEM. Statistically significant differences between control and experimental groups were determined using Multiple Student s t‐tests (two‐tailed, unpaired), one‐way ANOVA with Tukey multiple comparison difference test, Wilcoxon test, and log‐rank (Mantel‐Cox) test as indicated in the appropriate figure legend and text. Data distributions were assumed to be normal, but this was not formally tested. All statistical analyses were performed using GraphPad Prism (version 10) or R‐Studio (version 4.0). All experiments had at least three biological replicates.

### Ethics Approval Statement and Patient Consent Statement

All tissues were obtained from Department of Pathology, Jiangsu Cancer Hospital (Jiangsu Institute of Cancer Research & The Affiliated Cancer Hospital of Nanjing Medical University). All patients had signed informed consent for donating their samples.

## Conflict of Interest

The authors declare no conflict of interest.

## Author Contributions

X.S., Z.P., Y.Z., and W.Y. contributed equally to this work. Conceptualization: F.J., G.C.D. and X.M.S. Funding acquisition: X.L. and F.J. Methodology: Y.Z., Z.H.P., T.Z. and Y.Z.C. Investigation: X.M.S., W.M.Y., H.W., X.N.Y. and P.F.G. Visualization: X.M.S., R.T.L. and H.L.D. Supervision: F.J. and G.C.D. Writing‐ original draft: X.M.S., Writing‐review and editing: T.Z.

## Supporting information



Supporting Information

## Data Availability

All data used in this work can be acquired from the TCGA database (http://cancergenome.nih.gov/), ENCODE database (http://encodeproject.org/), CCLE database (http://sites.broadinstitute.org/ccle) and GEO database. The high‐throughput sequencing data in our study have been deposited in the Gene Expression Omnibus (GEO) under accession number GSE250097 and GSE250253. The processed data are available from the corresponding author upon reasonable request
