# Supplementary material for: Excessive MYC Orchestrates Macrophages induced Chromatin Remodeling to Sustain Micropapillary‐Patterned Malignancy in Lung Adenocarcinoma
Source: Adv Sci (Weinh). 2025 Feb 3;12(12):2403851. doi: 10.1002/advs.202403851 (PMC11948069; doi:10.1002/advs.202403851)
Supplement: Supplementary file 1 — Supporting Information [file ADVS-12-2403851-s001.docx]

**Supplementary Materials**

**Excessive MYC Orchestrates Macrophages induced Chromatin Remodeling to Sustain Micropapillary-Patterned Malignancy in Lung Adenocarcinoma**

Xuming Song^1,2,4*^, Zehao Pan^1,2,4*^, Yi Zhang^2,3*^, Wenmin Yang^1,2,5*^, Te Zhang^1,2,6^, Hui Wang^1,2,4^, Yuzhong Chen^1,2,4^, Xinnian Yu^1,2,4^, Hanlin Ding^1,2,4^, Rutao Li^1,2,7^, Pengfei Ge^1,2,4^, Lin Xu^1,2,8^, Gaochao Dong^1,2^, Feng Jiang^1,2^

1. Department of Thoracic Surgery, Nanjing Medical University Affiliated Cancer Hospital & Jiangsu Cancer Hospital & Jiangsu Institute of Cancer Research, 210009, Nanjing, P. R. China.
2. Jiangsu Key Laboratory of Molecular and Translational Cancer Research, Cancer Institute of Jiangsu Province, Nanjing, P. R. China.
3. Department of Pathology, Nanjing Medical University Affiliated Cancer Hospital & Jiangsu Cancer Hospital & Jiangsu Institute of Cancer Research, 210009, Nanjing, P. R. China.
4. The Fourth Clinical College of Nanjing Medical University, Nanjing, P. R. China.
5. Department of Pathology, Nanjing Drum Tower hospital, 210008, Nanjing, P.R. China.
6. Department of Biochemistry and Molecular Genetics, Feinberg School of Medicine, Northwestern University, Chicago, Illinois, USA.
7. Department of Thoracic Surgery, The First Affiliated Hospital of Soochow University, 215006, P. R. China.
8. Collaborative Innovation Center for Cancer Personalized Medicine, Nanjing Medical University, 211116, Nanjing, P. R. China.

* These authors contributed equally: Xuming Song, Zehao Pan, Yi Zhang and Wenmin Yang

Corresponding author:

Prof. Lin Xu e-mail: [xulin_83@hotmail.com](mailto:xulin_83@hotmail.com)

Prof. Feng Jiang e-mail: fengjiang_nj@njmu.edu.cn

Dr. Gaochao Dong: [gaochao_dong@njmu.edu.cn](mailto:gaochao_dong@njmu.edu.cn)

**List of Supplementary Materials**

**Figure S1** MP-subtype LUAD exhibits distinct tumor behavioral changes and is potentially closely associated with MYC pathway.

**Figure S2** Manipulating MYC alone in LUAD cell lines is insufficient to induce MP-pattern malignancy and its associated characteristics *in vitro*.

**Figure S3** Overexpression of MYC in xenograft tumor animal models fails to induce MP-pattern malignancy and its associated characteristics.

**Figure S4** The MP-subtype LUAD and MP-pattern cells are closely associated with M2-like macrophages.

**Figure S5** The induction of MP-pattern malignancy relies on macrophages in syngeneic transplant mice with bearing redundant MYC expression tumor.

**Figure S6** Redundant MYC expression along with M2-like macrophages, synergistically induces MP-pattern malignancy.

**Figure S7** Tumor cells with redundant MYC expression recruit M2-like macrophages from peripheral blood to the local microenvironment through CCL7/8-CCR2 chemotaxis.

**Figure S8** Redundant MYC expression enhances the transcriptional activity of CCL7 and CCL8.

**Figure S9** M2-like macrophages may promote MYC binding by increasing local chromatin accessibility through FOSL2.

**Figure S10** The transcriptional regulation of MP-pattern genes by MYC is dependent on the M2-like macrophage-TGFβ-FOSL2 axis.

**Figure S11** Activation of the M2-like macrophage-TGFβ-FOSL2 axis can rescue the transcriptional regulation of MP-pattern genes by MYC.

**Figure S12** FOSL2 is induced by TGFβ secreted by M2-like macrophages and serves as a protein-protein interaction partner of MYC.

**Figure S13** Histone acetylation involves transcriptional regulation and chromatin accessibility of MP-pattern genes co-mediated by MYC and M2-like macrophages

**Figure S14** Interfering with the M2-like macrophage-TGFβ-FOSL2 axis effectively suppresses MP-pattern malignancy.

**Table S1** Clinical information of 66 patients from MAPes corhort.

**Table S2** MP-pattern and AC-pattern genes from postmicrodissection bulk RNA-seq.

**Table S3** Morphological subtypes distribution in H&E staining slides from TCGA-LUAD dataset.

**Table S4** Oligonucleotide sequence in this study.


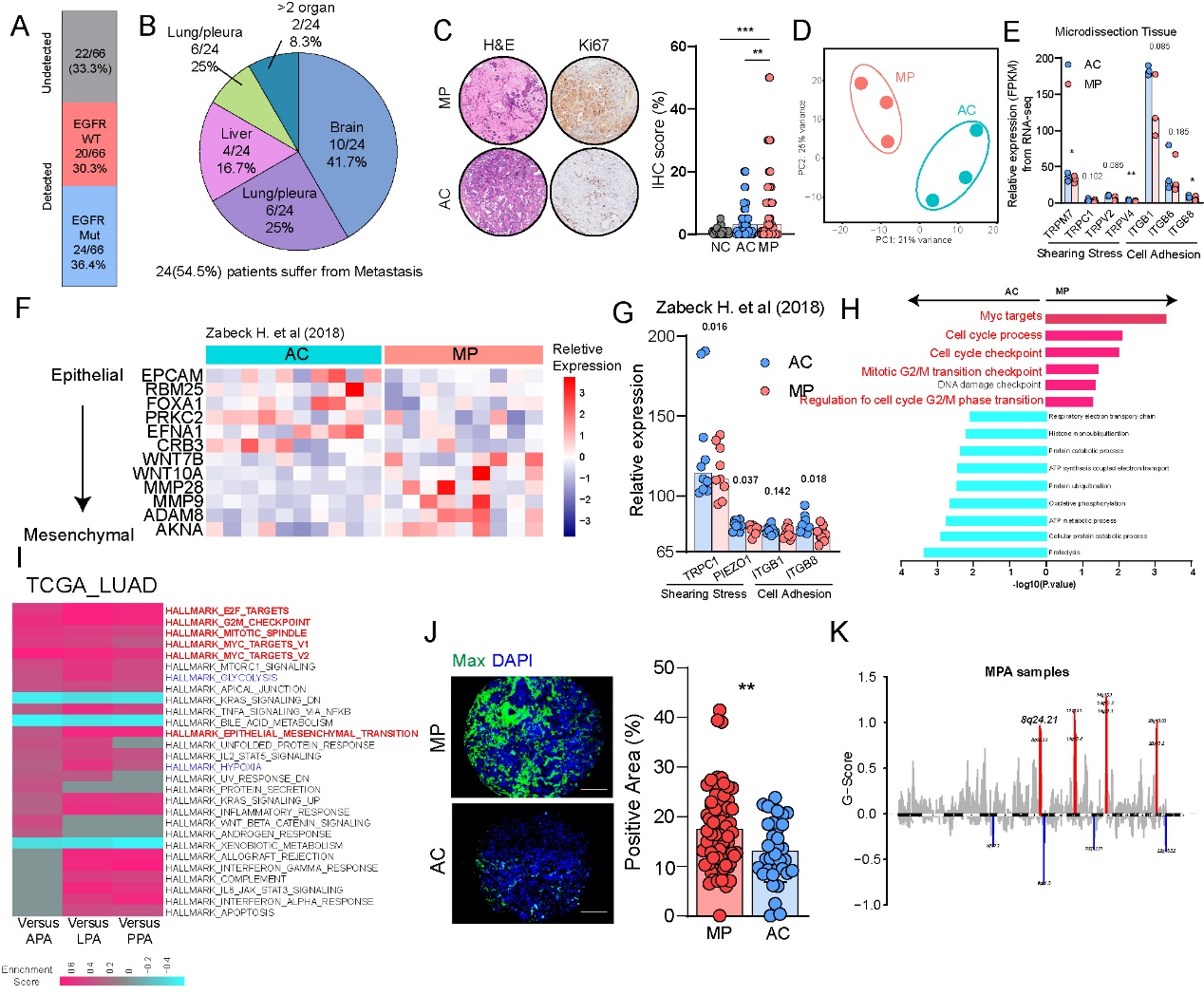


**Figure S1** **MP-subtype LUAD exhibits distinct tumor behavioral changes and is potentially closely associated with MYC pathway.**

(A-B) EGFR mutation status (A) and postoperative recurrence and metastasis (B) in the MAPes cohort of patients.

(C) Representative images of H&E staining and immunohistochemistry of Ki-67 in TMAs from MAPes cohort.

(D) Principal component analysis (PCA) of microdissected RNA-seq data from MP-subtype and AC-subtype tissues.

(E) FPKM of shearing force markers and cell adhesion markers from microdissected RNA-seq data.

(F-G) Relative expression of EMT markers (F), shearing force markers and cell adhesion markers (G) from Zabeck H et al. study.

(H) Pathway enrichment analysis of different expression genes between MP-subtype and AC-subtype tissue from microdissected RNA-seq data.

(I) The enrichment score from GSEA analysis between MP-subtype LUAD and low/mid-risk pattern.

(J) Representative images of immunofluorescence staining of Max in TAMs from MAPes cohorts. Left: Representative images, right: quantitative statistics.

(K) The G-score for predicted copy numbers from MP-subtype predominant LUAD tissue (MP-subtype>15%) from TCGA-LUAD dataset.


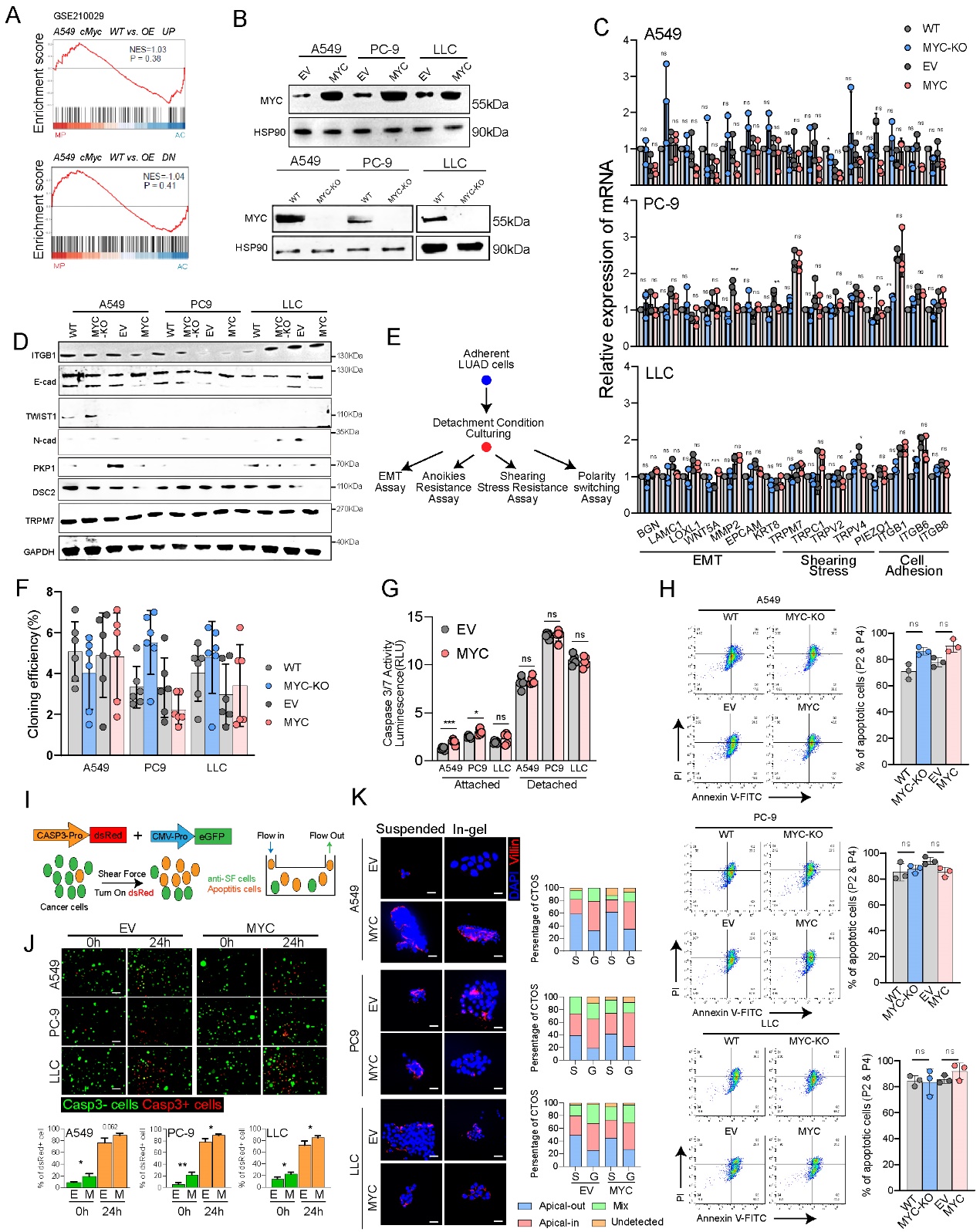


**Figure S2** **Manipulating MYC alone in LUAD cell lines is insufficient to induce MP-pattern malignancy and its associated characteristics *in vitro*.**

(A) GSEA of different expression genes between of wildtype A549 cells and MYC-overexpression A549 cells from FSE210029. Gene sets were constructed by different expression of MP-subtype and AC-subtype tissue bulk RNA-seq from RNA-seq after microdissection in Figure 1H.

(B) Western Blots reveals the efficiency of overexpression/knockout of MYC in cancer cell lines.

(C-D) The relative expression of markers of EMT, shearing stress and cell adhesion via qRT-PCR (C) and Western Blots (D) in MYC overexpressed/knockouted cancer cells.

(E) An experimental illustration showing the MP-pattern malignancy identification method *in vitro*.

(F-H) Non-anchored clonality ability assay (F) and detachment-induced cell death resistance assay via Caspase 3/7 activity assay (G) and Annexin V FACS assay (H) in MYC overexpressed/knockouted cancer cells. Indicated cells were allowed to grow in soft agar for 2 weeks and colonies were counted.

(I) An experimental illustration showing shearing force-induced apoptosis tracking system. No-apoptosis cancer cells show in single green, while apoptosis cancer cells show in orange (green overlap with red).

(J) Left: Representative images of LUAD cell lines transfected with a cell tracking system after 24 hours of sustained microvascular-like shear stress in indicated cells. Right: Representative images of in 0 hour and after 24 hours.

(K) Representative images of immunofluorescence staining of CTOS-like cultured cell lines derived from indicated cells in suspended conditions (suspended) and embedded in Matrigel ECM and cultured for 72 h (in gel). Red, villin; blue, DAPI. Scale bars: 20 μm. Right: Stacked bar graph depicting number of CTOSs with different apical status.


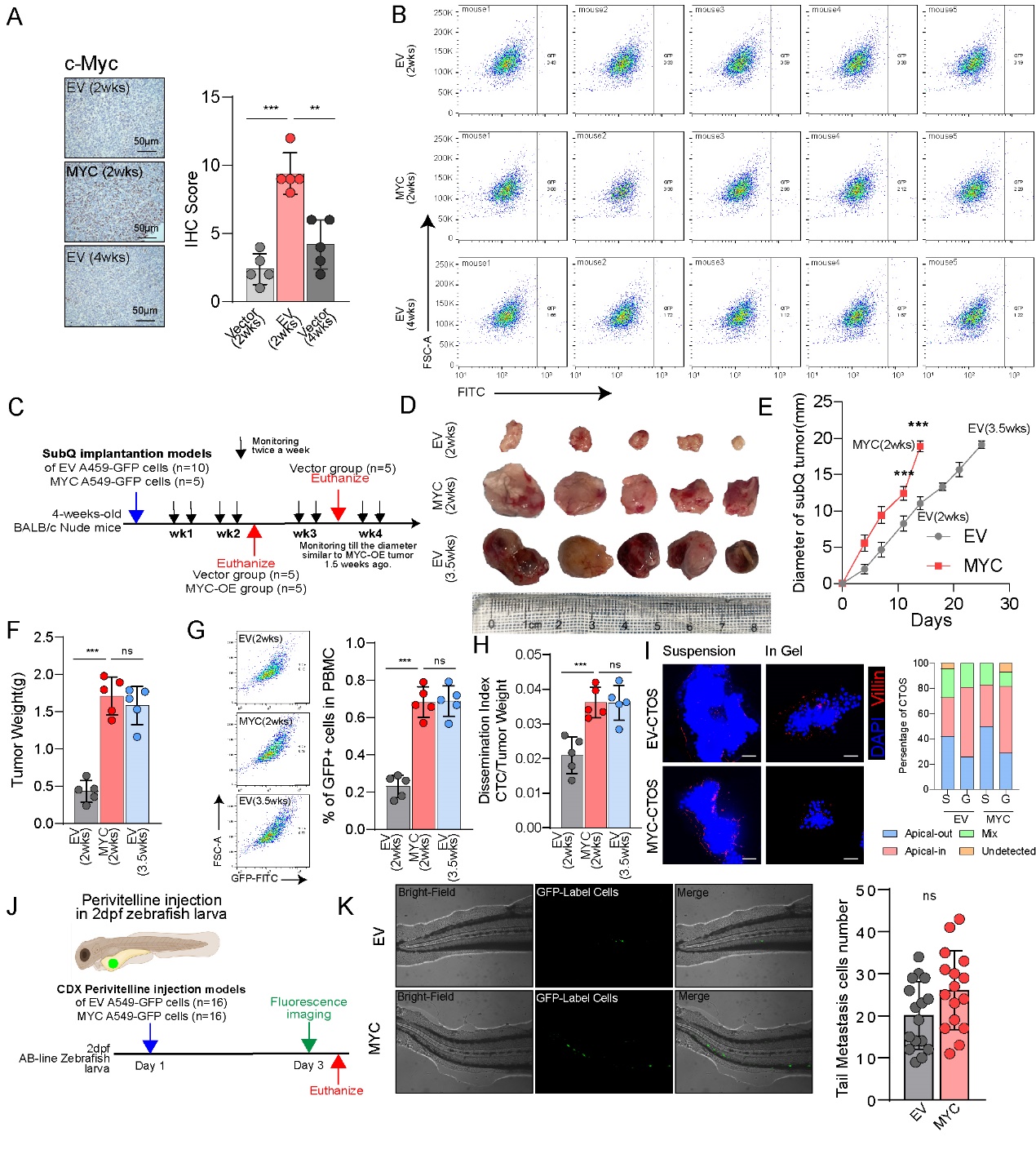


**Figure S3** **Overexpression of MYC in xenograft tumor animal models fails to induce MP-pattern malignancy and its associated characteristics.**

(A) Left: Representative images of immunohistochemistry of c-Myc from tumor indicated in Figure 2D. Right: quantitative statistics.

(B) CTCs detected from venous blood in indicated C57BL/6J mice.

(C) An experimental illustration showing experimental strategies for phenotypic characterization of MYC redundant expression tumors in athymic BALB/c Nude mice.

(D-F) Tumor images (D), growth kinetics (E), and weight of tumor (F) of subcutaneously implanted tumors from indicated BALB/c Nude mice.

(G) CTCs detected from venous blood in indicated BABL/c Nude mice. Left: Representative images, right: quantitative statistics.

(H) Dissemination index in indicated BALB/c Nude mice.

(I) Representative images of immunofluorescence staining of CTOSs derived from freshly dissociated from indicated tumors in suspended conditions (suspended) and embedded in Matrigel ECM and cultured for 72 h (in gel). Red, villin; blue, DAPI. Scale bars: 20 μm. Right: Stacked bar graph depicting number of CTOSs with different apical status.

(J) An experimental illustration showing experimental strategies for phenotypic characterization of MYC redundant expression tumors in AB-line wildtype 2 days past fertilization (dpf) zebrafish larva.

(K) Left: Representative images of larva tail tumor migration. Right: Representative images of indicated cells.


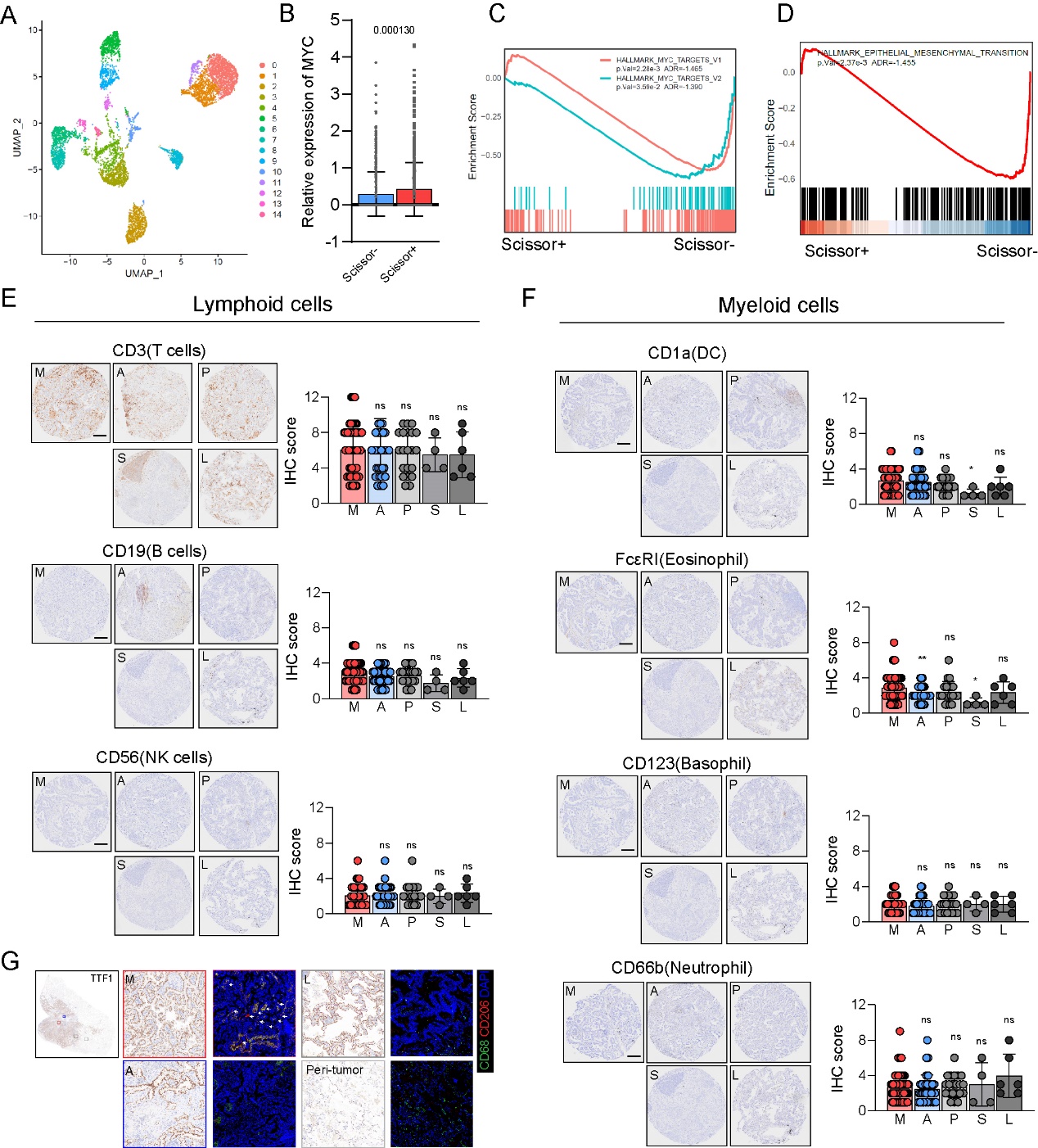


**Figure S4 The MP-subtype LUAD and MP-pattern cells are closely associated with M2-like macrophages.**

(A) Single-cell RNA sequencing (scRNA-seq) data of lung adenocarcinoma from Michael Bartoschek et al. study was utilized. UMAP cluster analysis were conducted in annotated cancer cells.

(B) Relative expression of MYC in SCISSOR+ cells (MP-subtype pronenes) and SCISSOR- cells (AC-subtype pronenes) from single-cell RNA-seq data in Figure 3A.

(C-D) GSEA of different expression genes between SCISSOR+ cells and SCISSOR- cells, which indicate enrichment MYC-target pathway (C) and EMT pathway (D) in SCISSOR+ cells.

(E-F) Representative images of immunohistochemistry of markers of serval lymphoid cells (E) and myeloid cells (F) in tissues micro-array from MAPes cohort.


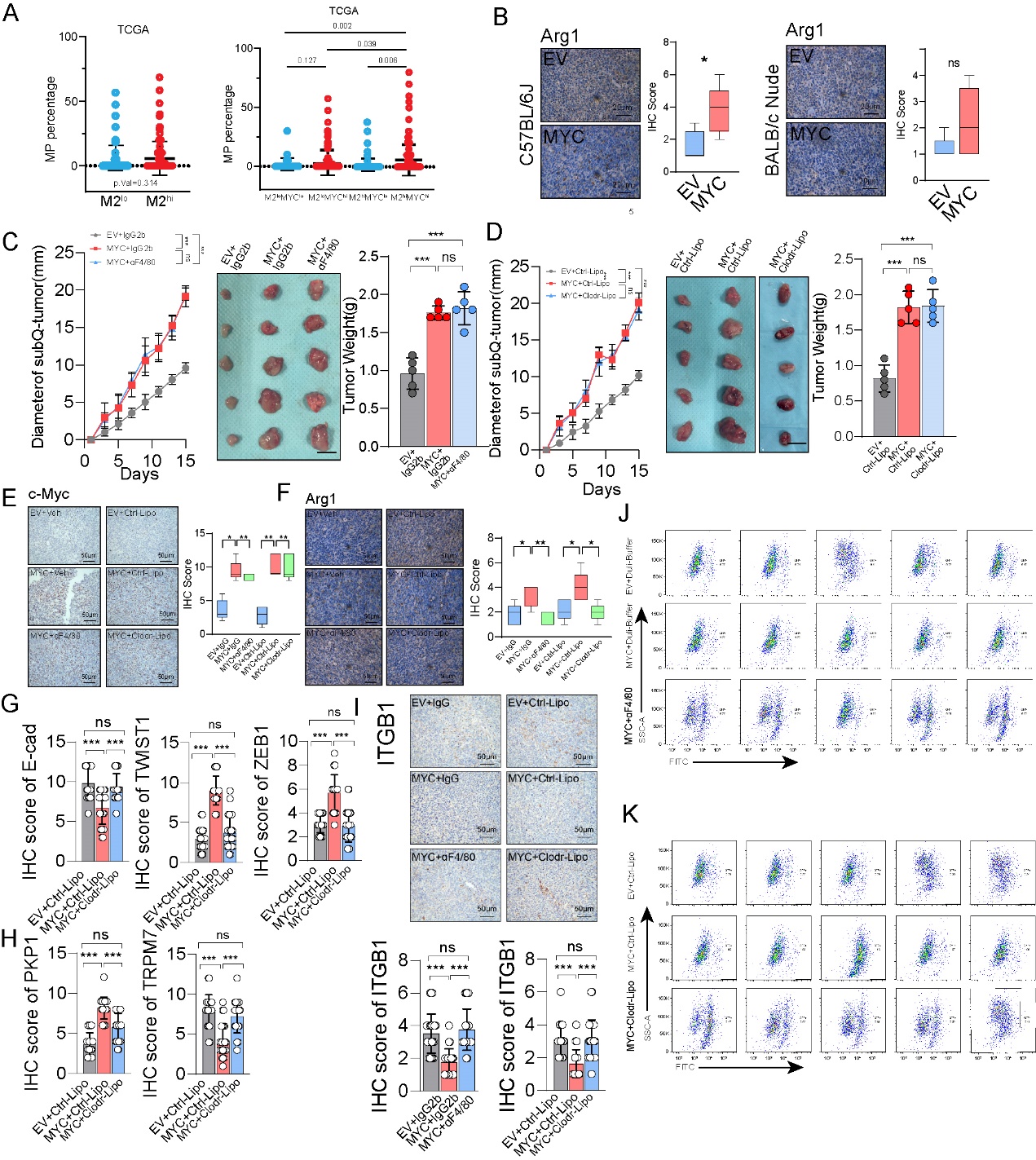


**Figure S5 The induction of MP-pattern malignancy relies on macrophages in syngeneic transplant mice with bearing redundant MYC expression tumor.**

(A) The difference of percentage of MP-subtype invasion area between M2-like macrophages-high infiltration samples (top 15%) and M2-like macrophages-low infiltration samples (bottom) (Left), moreover, between M2-like macrophages-low infiltration and MYC low expression samples, M2-like macrophages-high infiltration and MYC low expression samples, M2-like macrophages-low infiltration and MYC high expression samples and M2-like macrophages-high infiltration and MYC high expression samples (Right) from CIBERSORT deconvolution results via TCGA-LUAD mRNA dataset.

(B) Representative images of IHC of M2-macrophage marker Arg1 in subcutaneous tumor from Figure 2D (from C57BL/6J mice) and Supplementary Figure 3D (from BALB/c Nude mice). Right: quantitative statistics.

(C-D) Growth kinetics, tumor images and weight of tumor of subcutaneously implanted tumors in two distinct strategies depleting macrophages in mice with MYC redundant expression tumors. EV+IgG2b, subcutaneously tumor cell transfected with empty vector plasmid and mice were tail intravenous injected with IgG2b antibody solution. MYC+IgG2b, subcutaneously tumor cell transfected with MYC-overexpression plasmid and mice were tail intravenous injected with IgG2b antibody solution. MYC+αF4/80, subcutaneously tumor cell transfected with MYC-overexpression plasmid and mice were tail intravenous injected with αF4/80 neutralizing antibody solution (C). EV+Ctrl-Lipo, subcutaneously tumor cell transfected with empty vector plasmid and mice were tail intravenous injected with empty control liposome. MYC+Ctrl-Lipo, subcutaneously tumor cell transfected with MYC-overexpression plasmid and mice were tail intravenous injected with empty control liposome. MYC+Clodr-Lipo, subcutaneously tumor cell transfected with empty vector plasmid and mice were tail intravenous injected with clodronate liposome (D).

(F) Left: Representative images of immunohistochemistry of c-Myc (E) and Arg1 (F) from indicated tumors. Right: quantitative statistics.

(G-I) Quantitative statistics of immunohistochemistry results for E-cad, TWIST1, ZEB1 (G), TRPM7, PKP1 (H) in indicated tumors. Representative images and quantitative statistics of immunohistochemistry results for ITGB1 in indicated tumors (I)

(J-K) CTCs detected from venous blood in indicated mice.


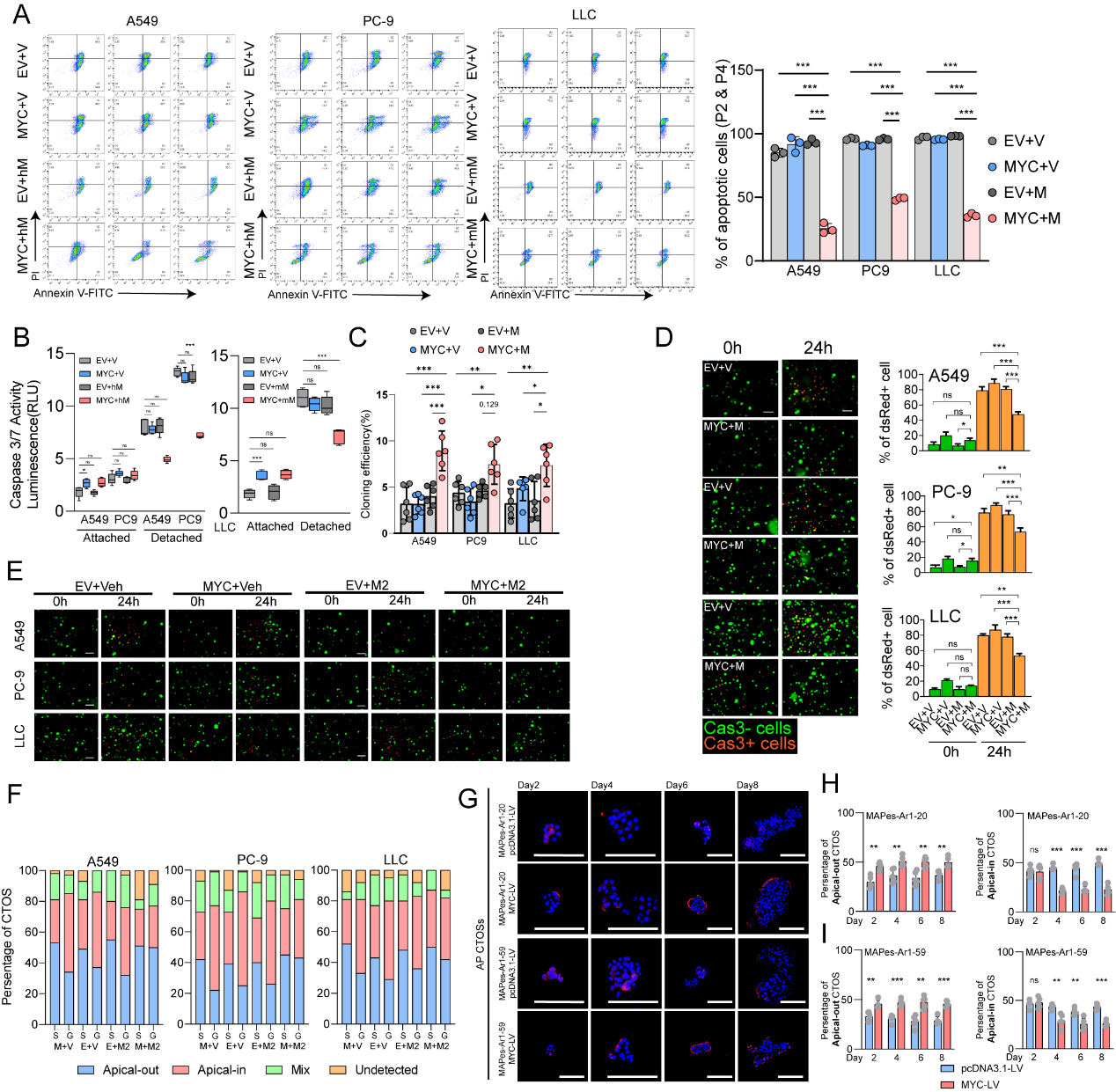


**Figure S6 Redundant MYC expression, along with M2-like macrophages, synergistically induces MP-pattern malignancy.**

(A) Left: Detachment-induced cell death resistance assay via Annexin V FACS assay in indicated cells. Right: quantitative statistics.

(B) Effect of MYC overexpression and M2-like macrophage co-culture on detachment-induced apoptosis detection. Lung cancer cell lines were cultured attached or detached on TC-treated plates or covalently bound hydrogel layer-treaded plates. Left: human cell lines; right: mice cell lines.

(C) Effect of MYC overexpression and M2-like macrophage co-culture on non-anchored clonality ability. Indicated cells were allowed to grow in soft agar for 2 weeks and colonies were counted.

(D-E) Representative images of LUAD cell lines transfected with a cell tracking system after 24 hours of sustained microvascular-like shear stress (D Left &E). D Right: Representative images of in 0 hour and after 24 hours.

(F) Stacked bar graph depicting number of indicated CTOSs with different apical status.

(G) Representative immunofluorescence staining images of AC-subtype tissue from LUAD patients infected with lentivirus (LV) packaged plasmids systems (plasmid backbone and MYC plasmid). Red, villin; blue, DAPI. Scale bars: 20 μm.

(H-I) Bar graph depicting number of apical-out CTOSs (Left) and apical-in CTOSs (Right) from Sample MAPes-Ar1-20 (F) and MAPes-Ar1-59 (G).


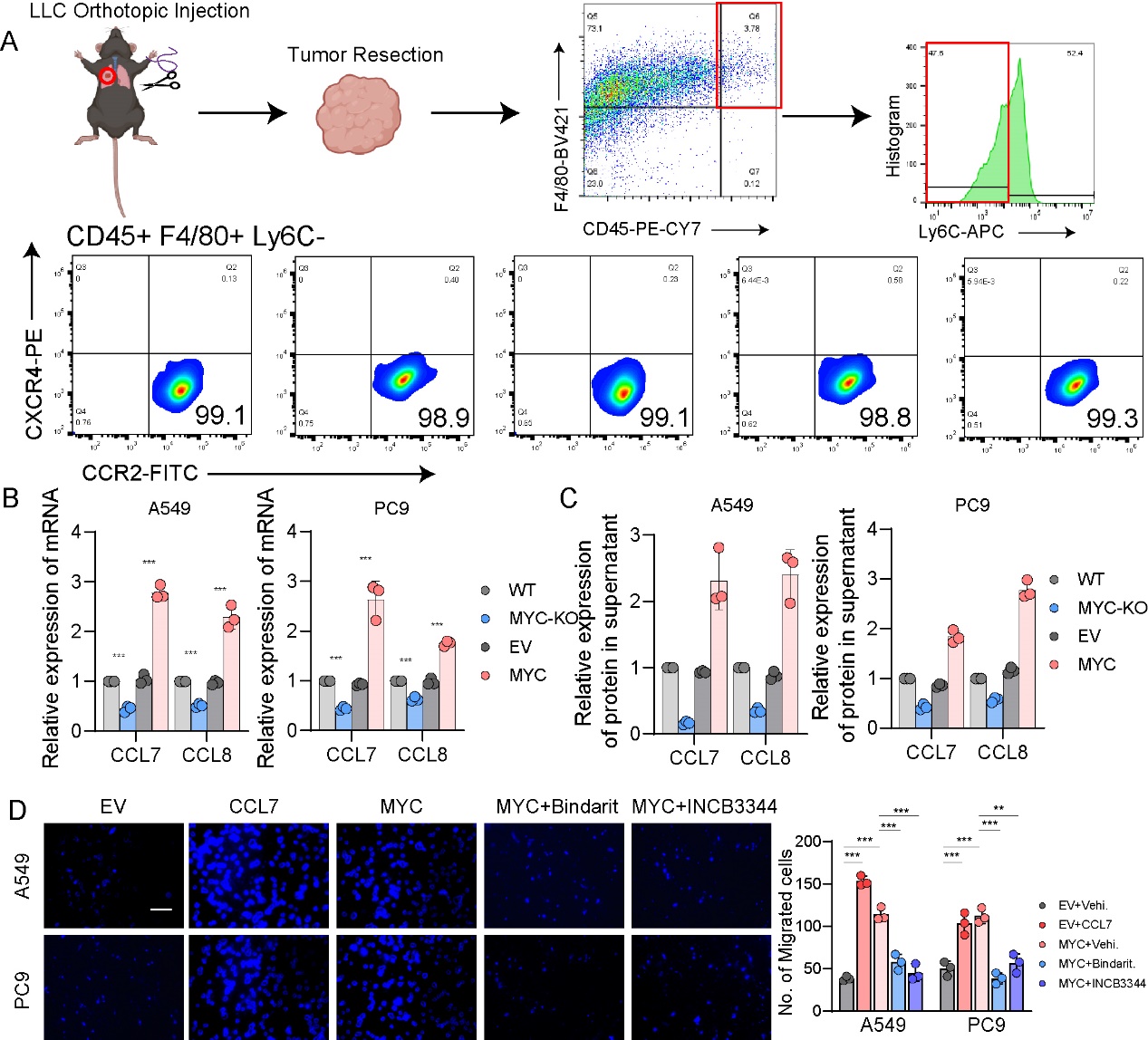


**Figure S7 Tumor cells with redundant MYC expression recruit M2-like macrophages from peripheral blood to the local microenvironment through CCL7/8-CCR2 chemotaxis.**

(A) An experimental illustration showing experimental strategies for sorting macrophages from lung tumors of LLC cell lung orthotopic injection C57BL/6J mice (Upper). CXCR4 and CCR2 clustering by flow cytometry in CD45+ F4/80+ and Ly6C- cells (Bottom).

(B) Relative expression of CCL7 and CCL8 mRNAs in indicated cells via qRT-PCR.

(C) Relative expression of CCL7 and CCL8 protein from indicated cells’ supernatant via ELISA.

(D) Representative images of macrophages chemotactic capacity of indicated cancer cells. Cancer cells and macrophages were co-cultured non-contractually in 8 μm transwell chambers above plates. Macrophages that migrated across the chamber were observed after 72 hours. DAPI were used for cell staining. Right: quantitative statistics.


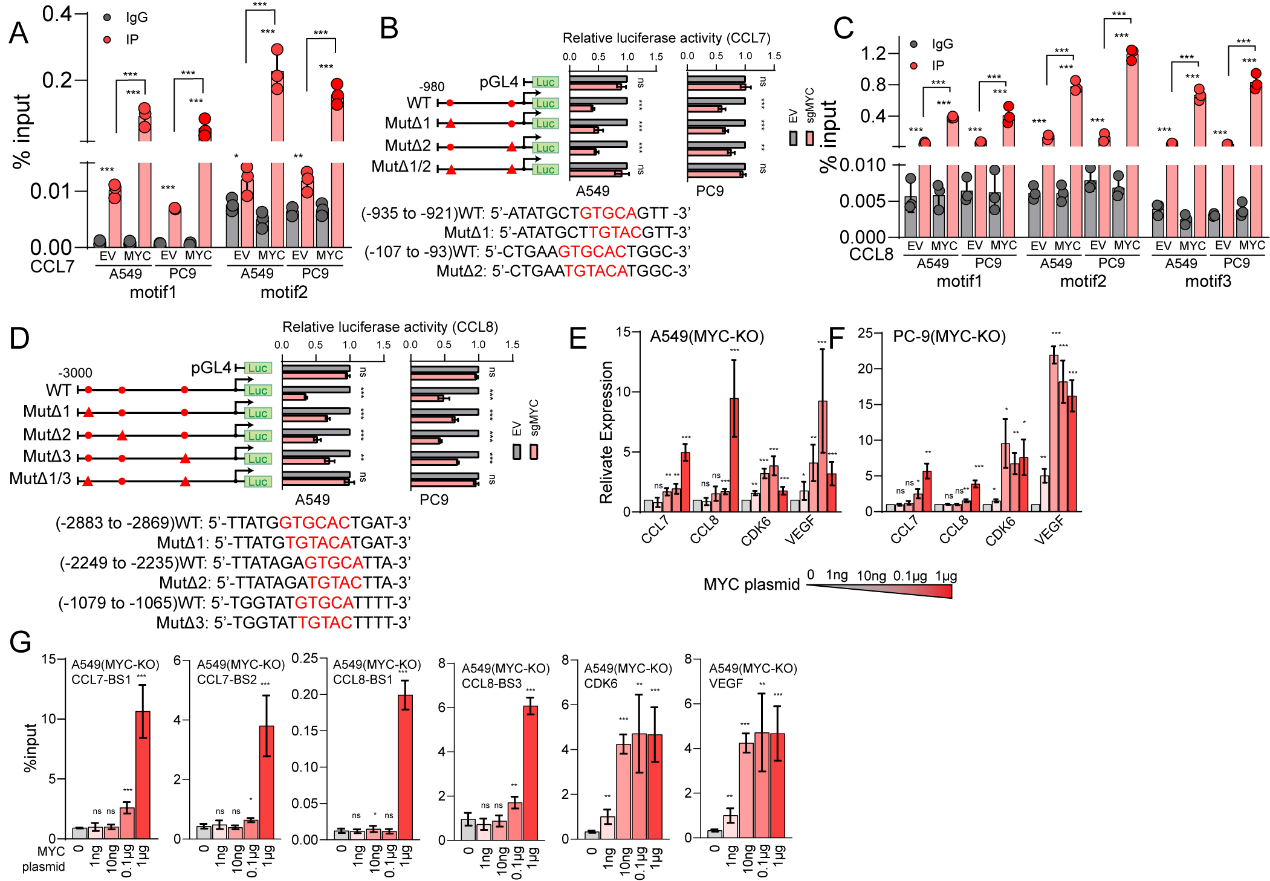


**Figure S8 Redundant MYC expression enhances the transcriptional activity of CCL7 and CCL8.**

(A) Anti-c-Myc ChIP-PCR of 2 predicted c-Myc binding motifs located in promoter of CCL7.

(B) In vitro dual-luciferase reporter genes systems, which consist of wildtype CCL7 promoter sequence or c-Myc binding motifs mutation CCL7 promoter sequences, and luciferase sequence. Renilla luciferase plasmid was co-transfected in indicated cells as internal reference.

(C) Anti-c-Myc ChIP-PCR of 3 predicted c-Myc binding motifs located in promoter of CCL8.

(D) In vitro dual-luciferase reporter genes systems, which consist of wildtype CCL8 promoter sequence or c-Myc binding motifs mutation CCL8 promoter sequences, and luciferase sequence. Renilla luciferase plasmid was co-transfected in indicated cells as internal reference.

(E-F) Relative expression of CCL7, CCL8, CDK6 and VEGF mRNA in indicated cells via qRT-PCR.

(G) Anti-c-Myc ChIP-PCR of validated binding sites of CCL7 and CCL8, moreover, predicted binding site of CDK6 and VEGF promoters.


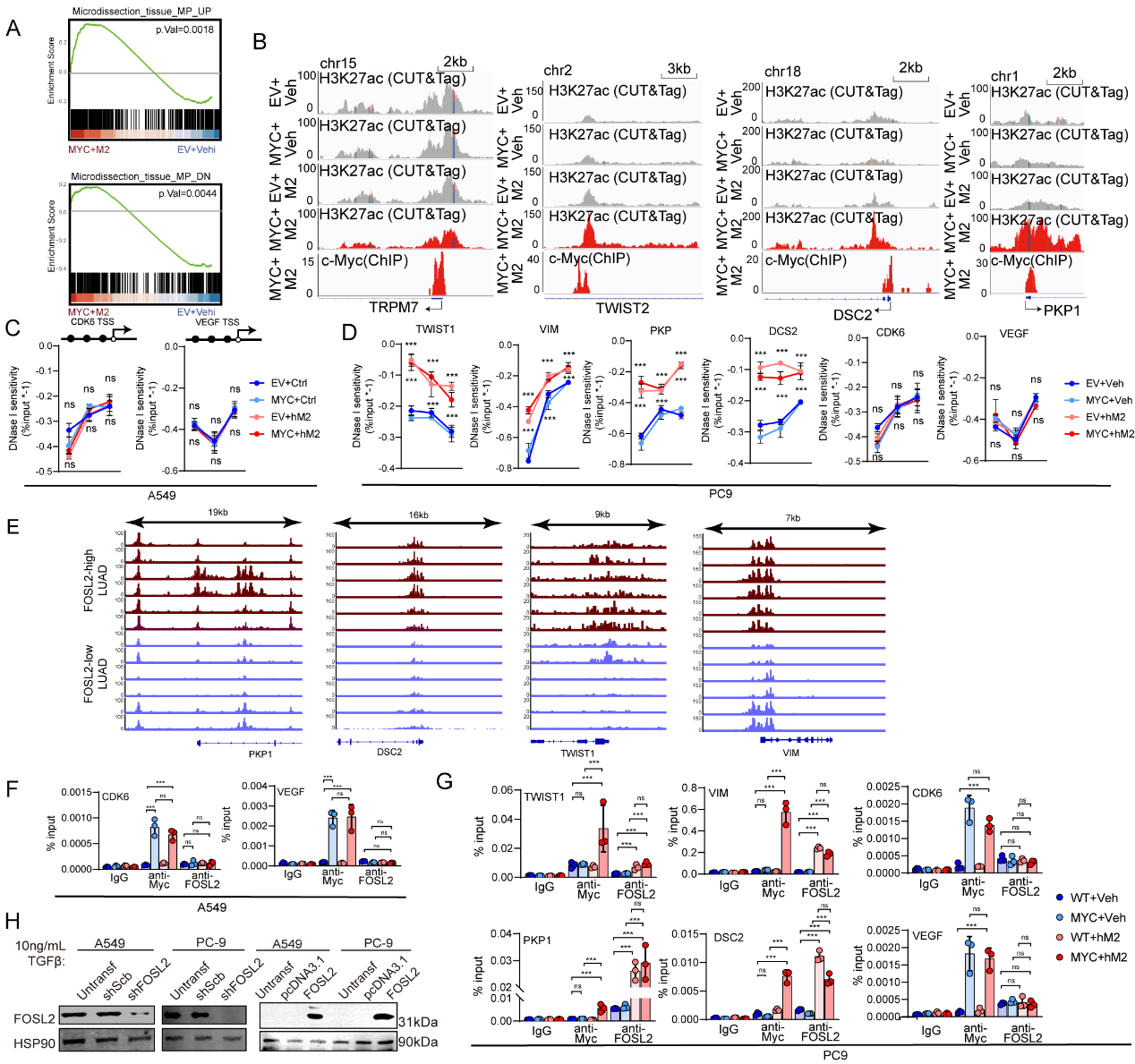


**Figure S9 M2-like macrophages may promote MYC binding by increasing local chromatin accessibility through FOSL2.**

(A) GSEA of different expression genes between MYC-overexpression plus M2-like macrophages cocultured cells and MYC-basal expression without M2-like macrophages cocultured cells. Gene sets were constructed by different expression of MP-subtype and Acinar-subtype tissue bulk RNA-seq from RNA-seq after microdissection in Figure 1H.

(B) Representative H3K27ac CUT&Tag-seq and MYC ChIP-seq tracks, showing significantly altered transcriptional regulatory element activity and MYC-binding located periphery of MP-pattern genes and MYC canonical target genes in E-V cells, M-V cells, E-M2 cells and M-M2 cells.

(C-D) The DNase I hypersensitivity site identified via DNase I treated-PCR in promoters of MP-pattern genes and MYC canonical target genes in indicated cells with different treatment.

(E) CoverageTracks visualization of ATAC-seq data from LUAD patients from TCGA dataset with high versus low FOSL2 expression, showing chromatin accessibility around gene loci of MP-pattern genes.

(F-G) ChIP-PCR of IgG, MYC and FOSL2 for binding promoters of MP-pattern genes and MYC canonical genes in indicated cells with different treatment.

(H) Western Blots reveals the efficiency of knockdown and overexpression of FOSL2 in cancer cell lines.


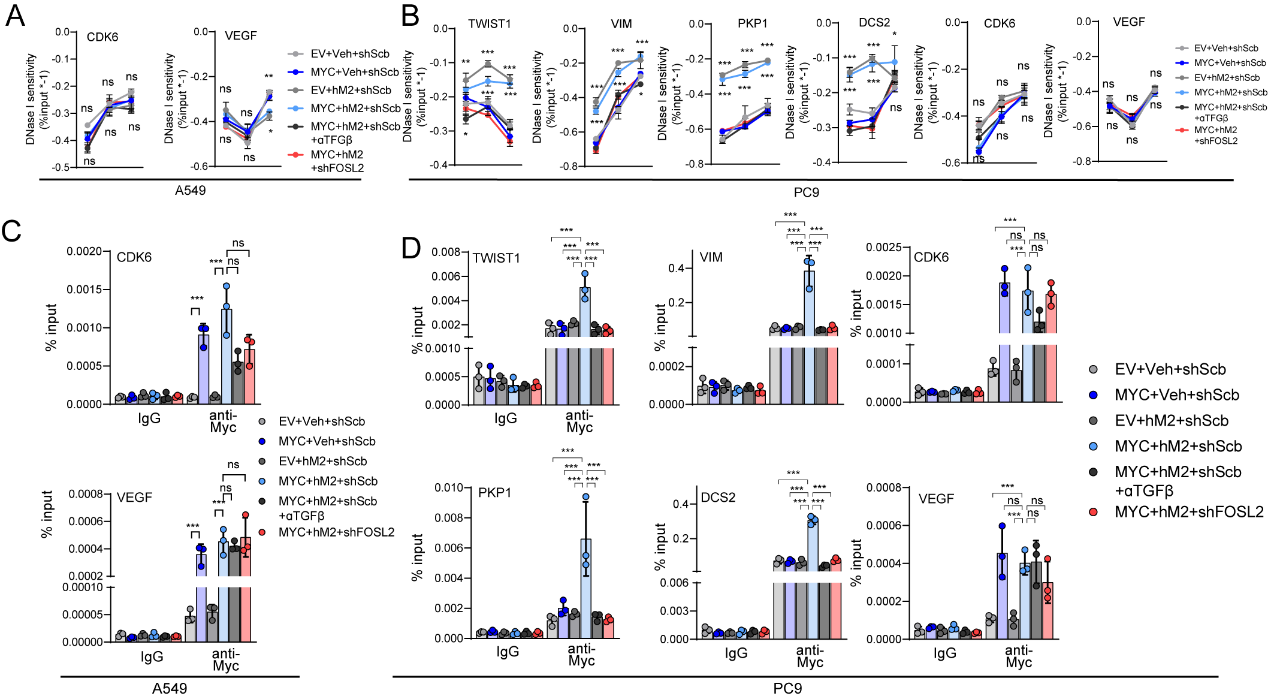


**Figure S10 The transcriptional regulation of MP-pattern genes by MYC is dependent on the M2-like macrophage-TGFβ-FOSL2 axis.**

(A-B) The DNase I hypersensitivity site identified via DNase I treated-PCR in promoters of MP-pattern genes and MYC canonical target genes in indicated cells with different treatment. The dependence experiment revealed that the high DNase I sensitivity of MP-pattern gene promoters induced by M2-like macrophages depends on TGFβ-FOSL2 axis.

(C-D) ChIP-PCR of IgG, MYC and FOSL2 for binding promoters of MP-pattern genes and MYC canonical target genes in indicated cells with different treatment. The dependence experiment revealed that MYC binding MP-pattern gene promoters in M-M2 A549 cells depends on TGFβ-FOSL2 axis.


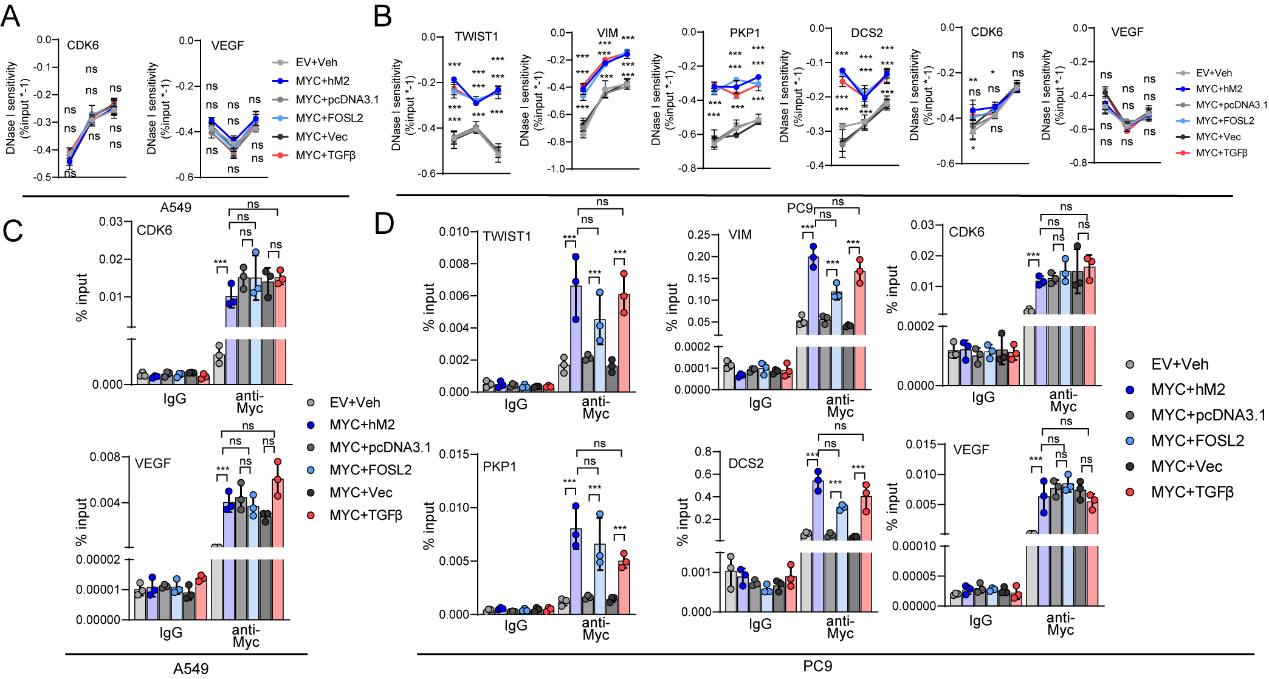


**Figure S11 Activation of the M2-like macrophage-TGFβ-FOSL2 axis can rescue the transcriptional regulation of MP-pattern genes by MYC.**

(A-B) The DNase I hypersensitivity site identified via DNase I treated-PCR in promoters of MP-pattern genes and MYC canonical target genes in indicated cells with different treatment. The rescue experiment revealed that the high DNase I sensitivity of MP-pattern gene promoters in solely MYC-overexpression A549 cells were rescued by FOSL2 overexpression or exogenous addition of TGFβ.

(C-D) ChIP-PCR of IgG, MYC and FOSL2 for binding promoters of MP-pattern genes and MYC canonical target genes in indicated cells with different treatment. The rescue experiment revealed that MYC binding MP-pattern gene promoters in M-V A549 cells were rescued by FOSL2 overexpression or exogenous addition of TGFβ.


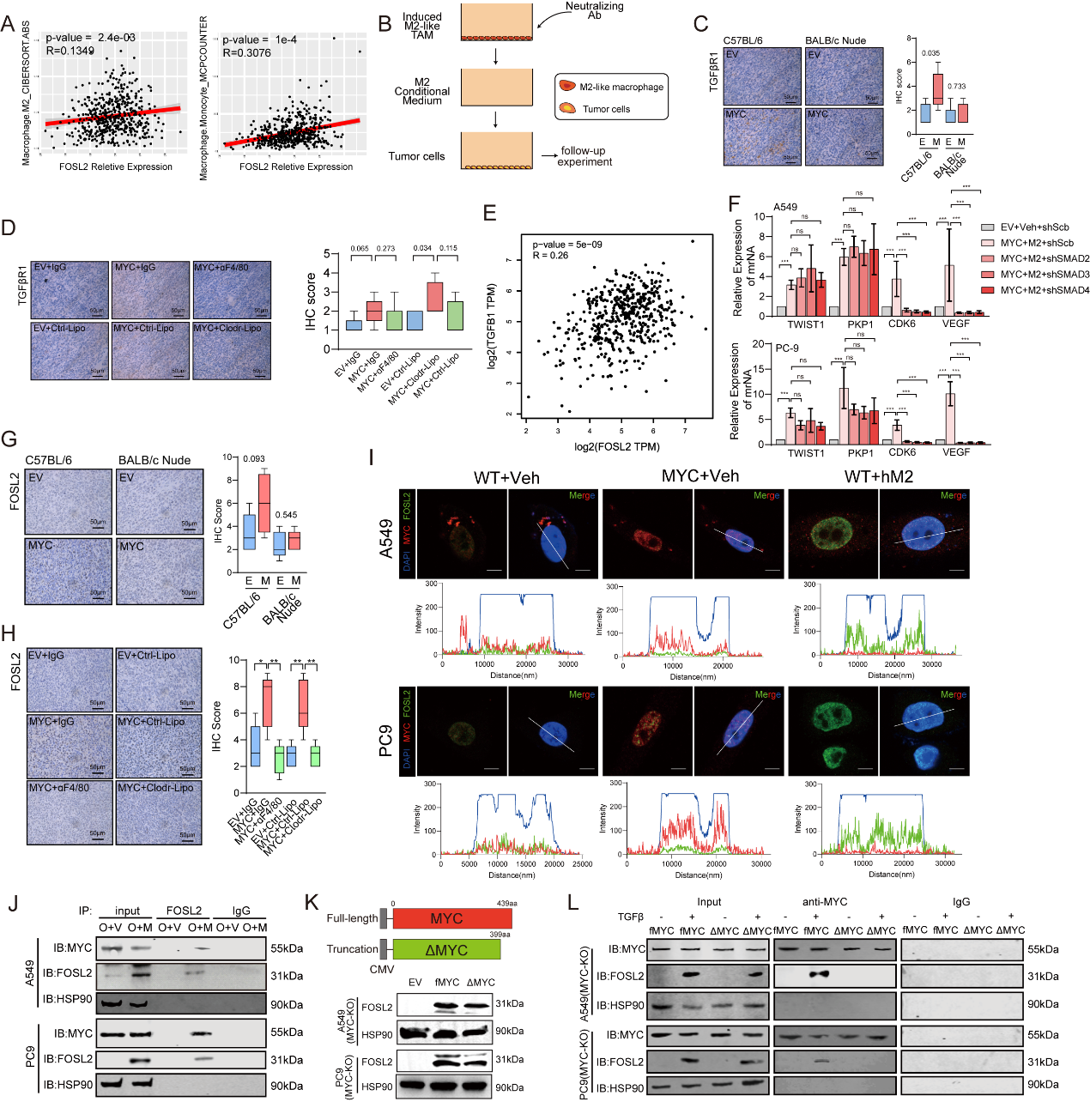


**Figure S12 FOSL2 is induced by TGFβ secreted by M2-like macrophages and serves as a protein-protein interaction partner of MYC.**

(A) Correlation analysis between FOSL2 expression and M2 macrophages (estimated by CIBERSORT ABS algorithm) and macrophages/monocytes (estimated by MCPcounter algorithm) in the TCGA-LUAD mRNA database using Spearman's rank correlation.

(B) An experimental illustration showing experimental strategies for tumor cell culture in M2-like macrophage conditional medium with indicated cytokines deletion.

(C-D) Representative images of immunohistochemistry of TGFβR1 in aforementioned tumor from Figure 2A, Supplementary Figure 2C and Figure 3F. Right: quantitative statistics.

(E) Correlation analysis between FOSL2 and TGFB1 expression using Spearman's rank correlation from TCGA-LUAD mRNA dataset.

(F) Relative expression of MP-pattern genes and MYC canonical target genes mRNAs via qRT-PCR in indicated cells.

(G-H) Representative images of immunohistochemistry of FOSL2 in subcutaneous tumor from Figure 2D, Supplementary Figure 3D (G) and Supplementary Figure 4C-D (H).

(I) Representative images of immunofluorescence of FOSL2 (green), MYC (red) and DAPI (blue) in E-V cells, M-V cells and E-M2 cells. Scale bars: 1μm (Upper). The detected fluorescence intensity at the white line (Bottom).

(J) Co-immunoprecipitation of FOSL2 antibody and IgG antibody indicated that FOSL2 binding MYC after MYC redundant expression and cancer cells were co-cultured with M2-like macrophages.

(K) The strategies for constructing full-length MYC plasmid and truncated MYC plasmid (Upper). Western Blots reveals the transfection efficiency of full-length MYC plasmid and truncated MYC plasmid in cancer cell lines (Bottom).

(L) Co-immunoprecipitation of c-Myc antibody and IgG antibody indicated that full-length MYC, but not truncated MYC, binding FOSL2 after redundant expression when cancer cells were co-cultured with M2-like macrophages.


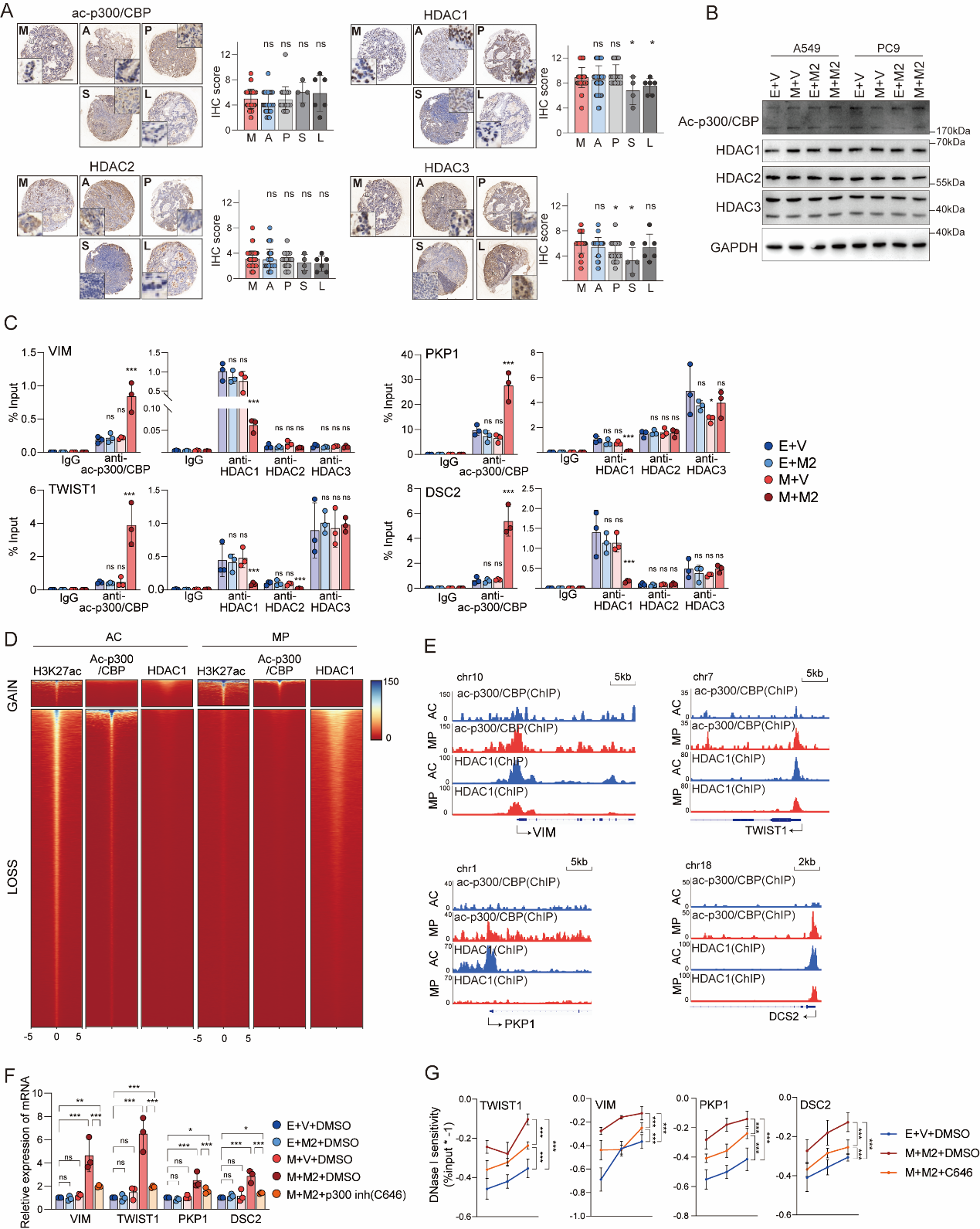


**Figure S13 Histone acetylation involves transcriptional regulation and chromatin accessibility of MP-patrern genes co-mediated by MYC and M2-like macrophages**

(A) Representative images of immunohistochemistry of ac-p300/CBP, HDAC1, HDAC2 and HDAC3 in tissues micro-array from MAPes cohort.

(B) Western Blots of ac-p300/CBP, HDAC1, HDAC2 and HDAC3 in LUAD cell lines.

(C) ChIP-PCR of IgG, ac-p300/CBP, HDAC1, HDAC2 and HDAC3 for binding promoters of MP-pattern genes in A549 cells with different treatment.

(D) Heatmaps of H3K27ac CUT&Tag-seq signals (same data as in Figure5A), ac-p300/CBP and HDAC1 ChIP-seq signals in MP-subtype tissue and AC-subtype tissue.

(E) Representative ac-p300/CBP and HDAC1 ChIP-seq tracks in MP-pattern genes loci.

(F) Relative expression of MP-pattern gene mRNAs in A549 cell with different treatments via qRT-PCR.

(G) The DNase I hypersensitivity site identified via DNase I treated-PCR in promoters of MP-pattern genes in A549 cells with different treatments.


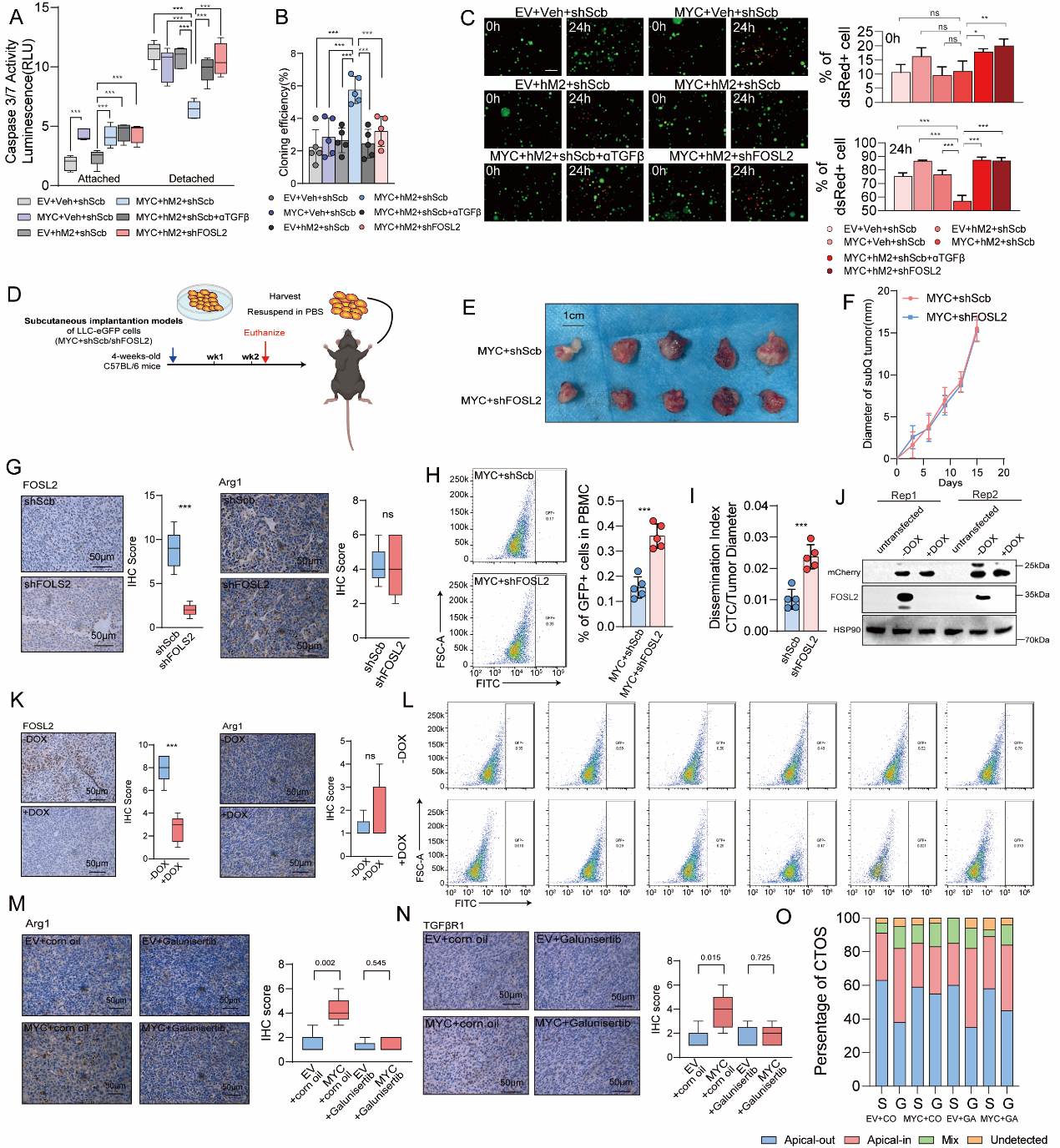


**Figure S14 Interfering with the M2-like macrophage-TGFβ-FOSL2 axis effectively suppresses MP-pattern malignancy.**

(A-C) The dependence experiment revealed that detachment-induced cell death resistance (A), non-anchored clonality ability (B) and anti-shearing force (C) induced by M2-like macrophages depends on TGFβ-FOSL2 axis in cancer cell lines in PC-9 cells. For detachment-induced cell death assay, Lung cancer cell lines were cultured attached or detached on TC-treated plates or covalently bound hydrogel layer-treaded plates. For non-anchored clonality ability, indicated cells were allowed to grow in soft agar for 2 weeks and colonies were counted.

(D-E) An experimental illustration showing the dependence experiment C57BL/6J mice with LLC cell subcutaneous homograft, which transfected with shFOSL2 plasmid or empty plasmid (D). Tumor images of subcutaneously implanted tumors from indicated mice (E).

(F) Growth kinetics of subcutaneously implanted tumors from indicated mice.

(G) Representative images of immunohistochemistry of FOSL2 and Arg1 in indicated tumor from supplementary Figure 13E. Right: quantitative statistics.

(H) CTCs detected from venous blood in indicated mice. Left: Representative images, right: quantitative statistics.

(I) Dissemination index in indicated mice.

(J) Western Blots reveals the transfection efficiency of FOSL2 tet-off system in A549 cells.

(K) Representative images of immunohistochemistry of FOSL2 and Arg1 in indicated tumor from Figure 7E. Right: quantitative statistics.

(L) CTCs detected from venous blood in indicated mice from Figure 7E.

(M-N) Representative images of immunohistochemistry of Arg1 and TGFβR1 in indicated tumor from Figure 7J. Right: quantitative statistics.

(O) Stacked bar graph depicting number of indicated CTOSs with different apical status.

**Table S1** Clinical information of 66 patients from MAPes corhort.


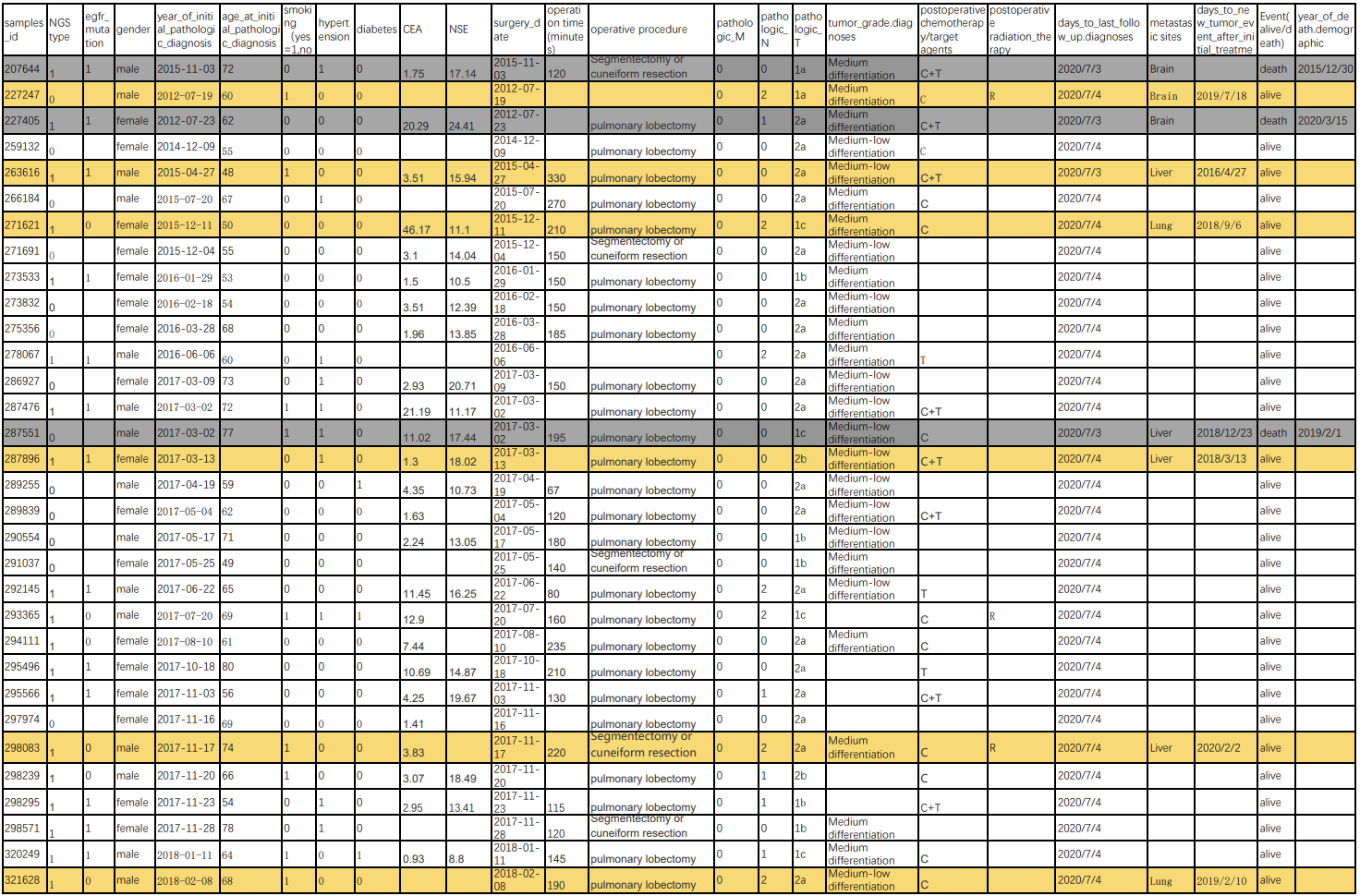


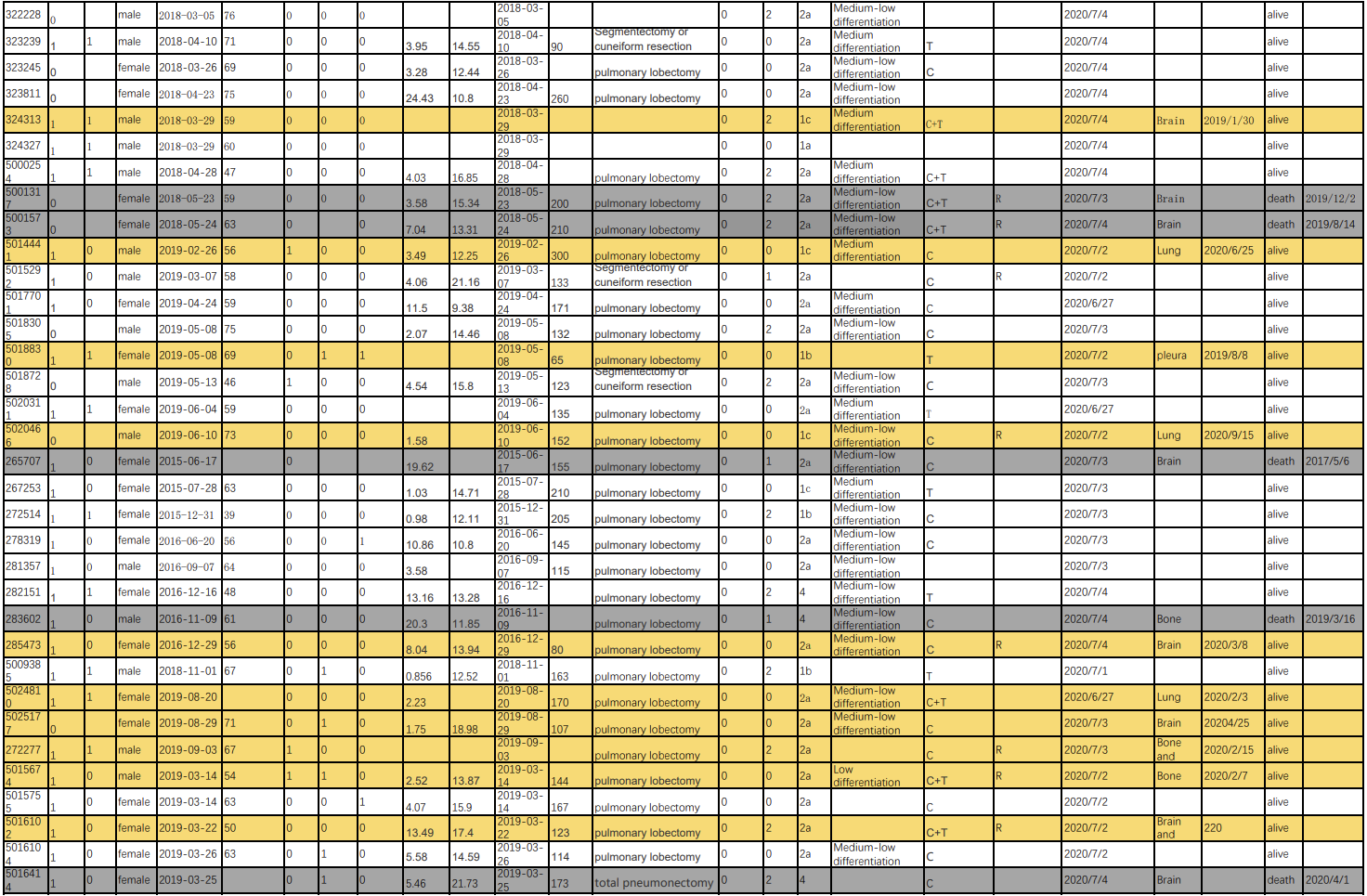


**Table S2** MP-pattern and AC-pattern genes from postmicrodissection bulk RNA-seq.

**Microdissection_MP_DN**

LEO1 KIF3B SYTL4 RP11-473I1.10 UPRT GOT2 TSR2 CPXM2 SNX15 PRKAR2A CTC-260E6.2 MIS12 GAR1 GLOD4 NDUFB6 RP11690I21.1 NTPCR LINC00984 PHYH FDX1 SMIM11 LEPRE1 ZNF639 BCL10 PLRG1 FAM199X DCTN5 LSS SNX7 ZCCHC14 WRNIP1 BCKDHB WDR78 RWDD2B EMC2 CPSF3 TMEM220 PIP4K2C ZBTB33 RPF2 KLF15 DYRK1B SRPX2 RP11-49O14.3 KPNA6 TBCAP3 RPS4XP3 PCTP EIF4HP1 AC009469.1 DLAT UBR7 ZFYVE1 ITGB8 MXI1 AL034548.1 RRN3 GTF2A1 TMEM14A TTC1 FBXO8 WRB LPCAT2 TEX2 CREB3 NDRG3 COMMD7 RP11-154F14.2 TMEM128 PHLDA3 TRPM7 ENDOD1 RAB3D SNRPB ME1 KCTD3 CRB3 MINA PPP1R8 NOL7 NDUFS1 MED21 TRIM44 ATL1 TSPO RNF141 NDUFA10 PPP1R3B SMAD5 NCEH1 RPF1 ALDH18A1 GSKIP MGAT2 ALG9 MAGEH1 MTCH2 FH FEZ1 RPS7P11 C6orf89 RNPEPL1 ZBTB4 RPS2P55 STOML2 MTIF3 TAF1B EIF1AXP1 HIST1H2AM ENC1 PDIA5 SIN3A ZNF652 SLC17A5 OSBPL9 SSBP1 SLBP CASK ADNP SEPHS1 MUT UFL1 NT5E USP16 HK2 RALA MAGOH ADRM1 ZNF395 PAG1 RDH11 TOMM22 FAM46A SSRP1 ZDHHC5 MRPL23 FRK POMK SEC22A UBTD2 HDGF SH3PXD2A PRPS1 ACBD3 EXT2 ALG2 PDPK1 STS MRPS24 ECHS1 TMEM57 MSRB2 PPIC CTDSPL FAM114A1 MRPL15 LLPH RANBP9 C10orf32 PSMB5 HS6ST1 IGHV1-46 OBFC1 FAM105A CCDC82 CPQ NREP PAGR1 CHRAC1 DYNLT3 UBE4A PSMD10 MKRN1 CDC27 PPP1R11 OPA1 NET1 HIST1H4L ENPP4 AC004797.1 AC069257.8 STMN1 FAM210B NEO1 PDGFC RTF1 SETD3 RP11- 1033A18.1 DCAF7 GALC PSMB2 GPI GOLGA5 PRR13 SFRP4 NDUFB7 ATAD1 CKAP5 FLT4 CD2AP PDCD10 DGCR2 ITGB1 POLR2G RGN MRFAP1L1 SNRPG TSNAX SLC30A5 RPP14 TSN P4HA1 RAC1P2 TMX1 ADI1 FAM3C VPS36 DCAF13 HINT3 ARL2BP GID8 ALKBH5 ESYT2 LNX2 FAM96B RABAC1 MGST2 LY96 EDEM3 KCTD10 TMEM179B SMDT1 VTI1B HPRT1 PCNA RHOG RDH14 SUCLG2 FTO OVCA2 MIEN1 NDUFA1 HTATIP2 ZMPSTE24 VKORC1 TRMT1L AMFR SUMO2P1 RNF6 SMIM20 SNRPC MRPS33 TMEM256 STAG2 RNH1 NPTN ZFX PGM3 ROBO2 EXT1 GOLGA7 C19orf33 RSU1 PSMC5 DUSP23 FTH1P8 C6orf106 LAMTOR3 NDFIP1 AAMDC CCT8 DESI2 VPS35 SMS APIP CTSF MPDU1 SMIM19 STARD10 API5 NDUFA6 ITFG1 MRPL13 H2AFZ HNMT RNF187 IARS2 VAPB RPS6KA3 SPPL3 POMP STT3A PIGT TRAF3IP2 UQCRC1 ABHD17C LRP10 UXT SUCLG1 MRPL14 DNAJC3 PTPLAD1 SEPHS2 PARK7 TIMMDC1 APEX1 ERGIC2 SETD7 IFRG15 RRM1 KIF1C PDCD6 RALY RNU2-6P MRPS6 FAM129A SH3BGRL2 C12orf49 IDH2 TNFRSF12A C2 SF3B4 SSU72 LGALS3 PSMB6 CLPTM1L NDUFA12 ESD PPP1R14B DNAJB6 PTPN11 ATP5G3 BCAP31 JTB RBBP4 EIF6 EI24 DRAP1 TMEM14B PAIP1 MRPL18 RAB5C RP11-386I14.4 CHMP4B USP9X FAM84B NDUFA5 C1QBP S100A16 KPNB1 PSMC1 TCEAL8 SSFA2 ARIH1 ESRP2 COPB2 PSMB3 MPC2 MS4A2 UBE2K SOAT1 HNRNPF EIF2S1 ANXA2P2 PAPSS1 FKBP11 COPS2 SUMO3 ATL3 EIF3I SMIM15 NDUFB10 COL10A1 GOLPH3 DDOST RP4-781K5.2 COX5A PSMA6 ALDH9A1 DYNLRB1 SUPT4H1 CHD4 PSMD2 S100A13 EIF5 YME1L1 DYNLT1 NDUFC1 PGRMC1 ZNF664 RPS27L RNF130 CNOT1 NUCB2 CDC42P6 SIL1 PSMB4 CDH1 SESN3 HSBP1 ZDHHC20 PSMA4 TAF10 HIST1H2AC TROVE2 RCN1 NDUFB2 TMOD3 PPCS CPNE3 BANF1 OSTC RTN3 SNURF MORF4L1P1 ABHD2 CCT5 EMB DDX6 TMCO1 RAB2A FUCA1 UQCR10 RNF11 LAMTOR5 VWA5A GNS SSR1 CUTA TMED5 TPD52 SDHD SMIM14 TMEM230 EIF5A RAB25 SOD1 PPIAP22 ERH DHRS7 SEC61G BNIP3L PCYOX1 PSMB1 PIEZO1 ARCN1 ALDH1A1 CHCHD2 TRAPPC1 TMEM87A ARF4 SCAMP2 MRFAP1 ST13 HMGN2 MPZL1 NDUFB1 RAB27A LAMP1 TM9SF2 QSOX1 HIST1H1C HIST1H1E LAMC1 ATP5F1 S100A11 CMPK1 FAM107B ARPC3 SEL1L3 GDI2 UBE2D3 RAN ALDH2 SPINT2 MORF4L1 TMEM66 SEC11C RAB11A CD46 PLS3 TUBB APP PGK1 SSR4 LPCAT1 SRP9 CAPRIN1 TMEM59 SCP2 SEP15 SRP14 ASAH1 IGKV2D-28 ITGB8 LAPTM4A KRT8 GPX3 LDHA YWHAE TMED10 HSP90AB1 GABARAP MAL2 PPIB HSP90B1 APLP2 IGKV1-9 TAGLN2 TSC22D3 TMED2 DSTN UBC PPIA HSP90AA1 HSPA8 GLUL RPS17L

**Microdissection_MP_UP**

RN7SK VIM CTD-2547E10.2 DENND3 MKLN1-AS1 NKTR HSPG2 RBM6 GLS CLK1 PHYKPL NFKBIZ NPC1 TNFAIP2 RP11-274B21.4 AKNA BAZ2A ZNF638 ARHGEF1 ADAM15 ANKRD36B RAPGEF5 TRIM22 YBX3 SDHAP1 MYC GRAMD1A ERV3-1 TRAPPC12 SLC20A1 ILF3 LETMD1 EXOC4 RP11-645C24.2 ARIH2 RUFY3 AKAP8L LRCH4 PCSK7 TCIRG1 UBA7 ZEB1 C17orf62 TWIST1 IST1 DOCK6 GRB10 TTLL3 DLG1 PNISR REC8 ATP9B SETD5-AS1 NEMF MTSS1L MTSS1 PMS2P3 RP11-1415C14.3 AMY2B ANK3 ZNF75A HTT INTS10 MAPK10 FAM118A PRR4 DCTN1 DOT1L FBRS MAU2 CAPS PABPN1 STAG3L3 ZACN AC000041.8 CDC25B BTAF1 SRRT GTF2IRD2B ARHGEF7 RN7SL648P GTPBP1 RN7SKP255 ODF3B TRAF5 CORO7 NUP188 MSL1 ABHD11 LENG8-AS1 C11orf80 LTBP3 PON3 NSUN5P1 RP11-440L14.1 TMEM110 EIF2B5 PIBF1 HSF1 WNT5A RP11-631M6.2 TNRC6A CDC42EP3 CDK5RAP2 CELF1 PHF12 C2orf68 IQCG IRF3 KRIT1 ZNF580 AHI1 NFAT5 RAP1AP EHBP1L1 ZNF117 ERBB2IP ATXN7 PILRB GTF2H2 RNF44 EIF4E2P1 AC010761.10 HSBP1L1 CARS2 TFB1M MLLT10 RP11-274B21.2 RP3340B19.5 CERS5 SFSWAP RP11-252A24.2 PPP6R2 BTF3P12 ZNF493 has-mir-6080 ACVRL1 RP11-274B21.3 NASP MIB2 DMPK DGKD RNF138 DMWD RP11-1277A3.2 PI4KA KMT2D GIGYF1 EPHA4 CYP3A5 TNIK WDR33 CDKL1 RN7SKP69 ADAMTS10 TPT1-AS1 NUP107 RP11-17G11.1 STX18 VRK3 DDX39A ZNF397 NR3C2 TMEM163 STX4 STX10 PNKP GALNT10 SLC38A6 SLC25A13 ASXL1 CROCCP3 UPF3A AC096921.2 HLA- DQB1-AS1 KIAA1683 ITSN2 GATS DNAJC21 ADCY4 GTF3C3 ZSCAN30 NECAP1 AC006042.8 IFT80 C2CD5 HMGB1P3 RAD52 DHX58 PRKD2 AGO3 NOP58 SPATS2 GTPBP4 DIP2A RP11-274B21.1 RALGAPB NUMB FASTKD1 WDR60 RP11-484D2.3 PANK4 RP11-333J10.2 HDAC4 KNTC1 RP11- 667K14.8 PEX1 MUM1 AC068580.5 GUSBP3 RPL32P3 HSD17B7 HDAC5 PCNXL2 CTD-2235C13.1 INPP4A VN1R83P DVL3 RFWD3 RP4-800G7.2 RP11-307C18.1 THEM4 BANP RP11-379K17.9 WASH3P RNF185-AS1 RP11-473M20.7 ACYP1 CDK9 TECPR1 RP11-345K20.2 CREBBP ENO3 RP11-697N18.4 CTB-108O6.2 LONP1 AC079807.3 ZMYM4-AS1 FLVCR1 NR1H3 NAIP HECTD3 DDX51 CC2D1A KIAA0355 RNF216 RN7SKP150 RP11- 170L3.4 RP11-157L3.5 COL9A2 TMEM41B GDPD5 FAM98A ADAMTS9-AS2 NFE2L3 RP11-775J23.2 STK11 ASH1L-IT1 NEK10 FMR1 RP11-137L10.6 GRB14 NR2F2-AS1 DMXL2 AC096582.7 AGAP8 FMN1 RP11-73M18.7 DNAH17 RNF138P1 RP11- 384C4.3 GPR125 MAATS1 CCDC37 PACS1 JARID2- AS1 LRTOMT RP11-15E18.5 RECQL4 RP11-13N13.6 AC005517.3 CWC25 AGAP5 NSFP1 RP11- 589F5.3 MET RGS17P1 GOLGA8O DTX3 DPY19L3 AL117190.2 ARAP3 QTRT1 SETD8 ZBTB48 NUSAP1 EXOSC8 ZNF138 ZNF354B CYB5B ZNF815P ADAMTS4 SIPA1L2 PCBD2 GPR89B ZNF512 MED14 CSRNP2 AC034193.5 TESK1 CEP44 FGD3 PFN1P4 ZNF37BP RP11-15H20.5 RP4-669P10.19 SEMA4C CLMN ZNF782 AC012513.4 JAG2 MTMR10 NSUN5P2 RP5-1042K10.13 CX3CL1 ZNF700 TRMT2B RP11-96D1.9 MAP3K14-AS1 RN7SL151P RPL32P1 CTA-357J21.1 ASH1L- AS1 TSPAN4 NRF1 KLHL3 ZBTB43 DSC2 RP4-740C4.5 TM4SF19 NUTM2D ATP6V1C2 NDUFAF4P4 RP11-554J4.1 RP11-477G18.2 PQLC2 RN7SKP198 BRIX1 SCLY RP11-15H20.6 C2orf74 STAG3L4 RP11-214K3.22 AGAP4 RP11-490K7.4 CTC-524C5.2 CKMT2-AS1 E2F3-IT1 RP11-272L13.4 RHOF GYLTL1B RPL9P32 AC018720.10 RP11-4F22.2 HDGFRP2 ZDHHC18 RAB2B HNRNPA1P49 DTHD1 C8orf44 STARD9 ZBTB11 WDR4 TAS2R18 C7orf26 AL450992.2 RABL6 ZNF610 TPM3P9 AC007952.5 GOLGA8Q ANKFY1 TNFRSF25 RP11-378J18.6 ISG20L2 DENND5A ANAPC4 RP11-307P22.1 PLGLB1 PPP1R2P3 RP11-329J18.4 DTX2P1- UPK3BP1-PMS2P11 CBWD3 RP11-840I19.5 RSRC1 SLC25A53 RP13-638C3.6 CA5B AGK GFOD1 TSGA10 RP11-1149O23.1 ZNF551 ZNF273 MTHFD2L PLEKHG4 RP11-296I10.6 ZNF274 LINC00852 AL118506.1 CTB-39G8.3 UBA52P6 BCAS4 AC004166.6 HMGB1P21 NT5C VN1R42P C16orf70 APEH TCOF1 MUS81 ERCC8 AC003989.4 CTB-175P5.2 RP5- 966M1.6 AGBL3 SCIN RP11-451H23.1 FAM134B AC023271.1 PKD1P1 EXD3 WDR83 RP11-214K3.21 VMAC RP11-136K14.1 GOLGA1 YJEFN3 AL391994.1 RP3-340B19.2 RP11-95I19.2 CTD-2260A17.1 KIAA1328 TAS2R15 ICA1L RP11-802E16.3 VNN3 RP5-1115A15.2 ZNF516 TNRC6C-AS1 RPL31P52 ZNF653 RP11-662B19.2 VPS9D1-AS1 NSUN6 DNAJC9 AP001625.6 TFAP4 ZNF717 TMEM183A ETV4 RP11-752G15.9 TP73-AS1 PRKD1 CTC- 487M23.8 NEU3 AC007875.2 KANSL2 ZNF714 RP11-175I17.2 SLMAP RP11- 602M11.4 SFMBT1 RP11-522L3.4 RP11-10N23.2 GLS2 MIR600HG NSUN5 MTND2P29 ZNF500 CTA-298G8.1 TRIM11 RP11-108K14.4 RP11-460E7.8 FTH1P3 PLEKHH1 GPRASP1 LAS1L INF2 CCT6P1 RP11-325E14.5 CXorf23 KANSL3 CTD-2666L21.2 PPIAP6 GPR132 ZBTB8OSP1 NGLY1 ZC3HC1 RPS26P52 RP11-104O19.2 RP11-203L2.3 HIST2H3D PKP1-B444P24.13 RP11-267J23.4 SURF6 ABCC6P2 AFAP1L2 AC068491.3 PBLD AC002306.1 AGBL4 RP11-439E19.6 NANOGP4 ZNF286B RP11-728K20.1 ESR2 C1orf173 ATG4D INPP5E RP11-384C4.6 ZNF230 AC010468.2 ZNF154 RP13-582O9.7 ZNF746 XXbac-B562F10.11 AC004893.11 GALNS XBP1P1 CRTC1 UBE3D RP1-199J3.7 AF178030.2 RNF215 SAMD1 HNRNPA3P11 AC116366.6 TRAF2 RP4-800M22.1 CACNB4 PDPK2 LETM1 C4orf48 SPTBN5 CACTIN RP11-146F11.1 PRELID1P3 MBNL1-AS1 MED26 DDX18P5 AC004461.4 GK3P RN7SL146P NAPEPLD CTA-797E19.2 DHX35 SNCG CTB-131B5.2 KB-431C1.4 TDRD10 HNRNPRP1 RP11-384C4.7 RN7SKP269 EIF4BP5 DUSP18 USP2 RP11-430C7.2 RP11-69E11.4 RP11-624L4.1 RP11-381O7.4 GS1-124K5.12 LARS2 RP11-53I6.4 CCT6P3

**Table S3** Morphological subtypes distribution in H&E staining slides from TCGA-LUAD dataset.

| ID | A(%) | P(%) | M(%) | L(%) | S(%) | gender |
| --- | --- | --- | --- | --- | --- | --- |
| TCGA-78-7155 | 5 | 15 | 0 | 0 | 80 | MALE |
| TCGA-50-6597 | 8 | 80 | 0 | 2 | 10 | FEMALE |
| TCGA-73-A9RS | 0 | 0 | 0 | 0 | 100 | MALE |
| TCGA-44-7667 | 0 | 0 | 0 | 0 | 100 | FEMALE |
| TCGA-78-7163 | 30 | 70 | 0 | 0 | 0 | MALE |
| TCGA-MP-A4T6 | 100 | 0 | 0 | 0 | 0 | FEMALE |
| TCGA-55-8087 | 90 | 10 | 0 | 0 | 0 | FEMALE |
| TCGA-55-6642 | 55 | 40 | 5 | 0 | 0 | MALE |
| TCGA-49-AARQ | 20 | 20 | 0 | 0 | 60 | FEMALE |
| TCGA-05-4397 | 0 | 0 | 0 | 0 | 100 | MALE |
| TCGA-05-4420 | 0 | 100 | 0 | 0 | 0 | MALE |
| TCGA-55-8508 | 0 | 0 | 0 | 0 | 0 | FEMALE |
| TCGA-69-7764 | 70 | 0 | 0 | 30 | 0 | MALE |
| TCGA-86-8359 | 0 | 70 | 30 | 0 | 0 | MALE |
| TCGA-97-A4M7 | 30 | 0 | 0 | 70 | 0 | MALE |
| TCGA-97-A4M7 | 70 | 25 | 5 | 0 | 0 | MALE |
| TCGA-86-8668 | 20 | 0 | 0 | 40 | 40 | FEMALE |
| TCGA-97-A4M1 | 40 | 0 | 0 | 60 | 0 | FEMALE |
| TCGA-49-AAQV | 45 | 50 | 5 | 0 | 0 | FEMALE |
| TCGA-78-7540 | 20 | 80 | 0 | 0 | 0 | FEMALE |
| TCGA-86-8054 | 50 | 0 | 0 | 0 | 50 | MALE |
| TCGA-49-4512 | 0 | 0 | 0 | 0 | 0 | FEMALE |
| TCGA-49-4512 | 0 | 0 | 0 | 0 | 0 | FEMALE |
| TCGA-49-4512 | 0 | 0 | 0 | 0 | 0 | FEMALE |
| TCGA-49-4512 | 0 | 0 | 0 | 0 | 0 | FEMALE |
| TCGA-49-4512 | 0 | 0 | 0 | 0 | 0 | FEMALE |
| TCGA-49-4512 | 0 | 0 | 0 | 0 | 0 | FEMALE |
| TCGA-49-4512 | 0 | 0 | 0 | 0 | 0 | FEMALE |
| TCGA-49-4512 | 20 | 70 | 10 | 0 | 0 | FEMALE |
| TCGA-J2-8192 | 100 | 0 | 0 | 0 | 0 | FEMALE |
| TCGA-49-4490 | 0 | 0 | 0 | 0 | 0 | FEMALE |
| TCGA-49-4490 | 0 | 0 | 0 | 0 | 0 | FEMALE |
| TCGA-49-4490 | 0 | 0 | 0 | 0 | 0 | FEMALE |
| TCGA-49-4490 | 0 | 0 | 0 | 0 | 0 | FEMALE |
| TCGA-49-4490 | 20 | 60 | 20 | 0 | 0 | FEMALE |
| TCGA-49-4490 | 20 | 50 | 30 | 0 | 0 | FEMALE |
| TCGA-50-6594 | 0 | 0 | 0 | 0 | 100 | FEMALE |
| TCGA-44-6776 | 10 | 40 | 20 | 30 | 0 | FEMALE |
| TCGA-55-8203 | 70 | 0 | 10 | 0 | 10 | FEMALE |
| TCGA-86-8281 | 10 | 60 | 20 | 0 | 0 | MALE |
| TCGA-78-8662 | 15 | 10 | 5 | 30 | 40 | FEMALE |
| TCGA-78-7535 | 20 | 30 | 20 | 0 | 30 | MALE |
| TCGA-44-A479 | 10 | 0 | 0 | 0 | 90 | FEMALE |
| TCGA-55-7910 | 100 | 0 | 0 | 0 | 0 | FEMALE |
| TCGA-75-6212 | 0 | 0 | 0 | 0 | 0 | FEMALE |
| TCGA-78-7159 | 40 | 60 | 0 | 0 | 0 | FEMALE |
| TCGA-44-5645 | 100 | 0 | 0 | 0 | 0 | FEMALE |
| TCGA-05-4396 | 0 | 0 | 0 | 0 | 0 | MALE |
| TCGA-91-A4BC | 0 | 0 | 0 | 0 | 100 | MALE |
| TCGA-71-8520 | 25 | 50 | 5 | 0 | 20 | FEMALE |
| TCGA-97-8552 | 50 | 0 | 20 | 30 | 0 | FEMALE |
| TCGA-05-5715 | 0 | 0 | 0 | 0 | 0 | FEMALE |
| TCGA-L9-A7SV | 40 | 60 | 0 | 0 | 0 | MALE |
| TCGA-69-7980 | 60 | 0 | 15 | 20 | 5 | FEMALE |
| TCGA-64-1681 | 80 | 0 | 20 | 0 | 0 | FEMALE |
| TCGA-55-8615 | 0 | 0 | 0 | 0 | 0 | MALE |
| TCGA-64-5774 | 80 | 20 | 0 | 0 | 0 | MALE |
| TCGA-50-7109 | 95 | 0 | 5 | 0 | 0 | MALE |
| TCGA-73-7498 | 40 | 55 | 5 | 0 | 0 | FEMALE |
| TCGA-O1-A52J | 15 | 25 | 0 | 60 | 0 | FEMALE |
| TCGA-86-8585 | 0 | 0 | 10 | 0 | 90 | MALE |
| TCGA-55-1592 | 20 | 60 | 20 | 0 | 0 | MALE |
| TCGA-75-6203 | 0 | 0 | 0 | 0 | 0 | FEMALE |
| TCGA-95-A4VK | 0 | 0 | 0 | 0 | 0 | FEMALE |
| TCGA-49-4514 | 0 | 0 | 0 | 0 | 0 | FEMALE |
| TCGA-49-4514 | 50 | 15 | 5 | 0 | 30 | FEMALE |
| TCGA-49-4514 | 0 | 80 | 20 | 0 | 0 | FEMALE |
| TCGA-49-4514 | 0 | 50 | 50 | 0 | 0 | FEMALE |
| TCGA-55-8094 | 0 | 0 | 0 | 0 | 0 | MALE |
| TCGA-49-4494 | 80 | 0 | 0 | 0 | 10 | MALE |
| TCGA-49-4494 | 0 | 0 | 0 | 0 | 0 | MALE |
| TCGA-49-4494 | 0 | 0 | 0 | 0 | 0 | MALE |
| TCGA-49-4494 | 0 | 0 | 0 | 0 | 0 | MALE |
| TCGA-49-4494 | 0 | 0 | 0 | 0 | 0 | MALE |
| TCGA-49-4494 | 0 | 0 | 0 | 0 | 0 | MALE |
| TCGA-49-4494 | 0 | 0 | 0 | 0 | 0 | MALE |
| TCGA-78-7537 | 10 | 40 | 50 | 0 | 0 | MALE |
| TCGA-69-8253 | 50 | 20 | 30 | 0 | 0 | FEMALE |
| TCGA-38-4628 | 20 | 0 | 80 | 0 | 0 | FEMALE |
| TCGA-44-A47B | 0 | 15 | 0 | 0 | 85 | MALE |
| TCGA-97-A4M6 | 30 | 0 | 0 | 70 | 0 | FEMALE |
| TCGA-86-6851 | 100 | 0 | 0 | 0 | 0 | FEMALE |
| TCGA-05-4402 | 80 | 10 | 10 | 0 | 0 | FEMALE |
| TCGA-55-7728 | 95 | 0 | 0 | 0 | 10 | FEMALE |
| TCGA-53-7813 | 100 | 0 | 0 | 0 | 0 | FEMALE |
| TCGA-78-7161 | 70 | 0 | 0 | 0 | 30 | FEMALE |
| TCGA-86-7954 | 30 | 15 | 10 | 0 | 45 | FEMALE |
| TCGA-J2-A4AD | 100 | 0 | 0 | 0 | 0 | FEMALE |
| TCGA-55-8097 | 50 | 0 | 0 | 50 | 0 | FEMALE |
| TCGA-55-8206 | 100 | 0 | 0 | 0 | 0 | MALE |
| TCGA-55-7727 | 0 | 0 | 60 | 0 | 40 | MALE |
| TCGA-73-7499 | 70 | 0 | 0 | 0 | 30 | FEMALE |
| TCGA-93-A4JN | 20 | 70 | 10 | 0 | 0 | MALE |
| TCGA-86-8280 | 20 | 70 | 10 | 0 | 0 | FEMALE |
| TCGA-49-6743 | 0 | 0 | 0 | 0 | 0 | FEMALE |
| TCGA-49-6743 | 0 | 0 | 0 | 0 | 100 | FEMALE |
| TCGA-49-6743 | 0 | 0 | 0 | 0 | 0 | FEMALE |
| TCGA-49-6743 | 0 | 0 | 0 | 0 | 0 | FEMALE |
| TCGA-05-4382 | 0 | 0 | 0 | 0 | 0 | MALE |
| TCGA-86-8055 | 0 | 0 | 90 | 0 | 0 | MALE |
| TCGA-73-4675 | 0 | 0 | 50 | 0 | 0 | MALE |
| TCGA-71-6725 | 20 | 30 | 30 | 0 | 0 | FEMALE |
| TCGA-49-AARE | 20 | 0 | 10 | 0 | 70 | FEMALE |
| TCGA-64-5779 | 0 | 0 | 0 | 0 | 100 | MALE |
| TCGA-78-7166 | 0 | 0 | 0 | 0 | 0 | MALE |
| TCGA-44-2661 | 40 | 50 | 10 | 0 | 0 | FEMALE |
| TCGA-86-8279 | 100 | 0 | 0 | 0 | 0 | MALE |
| TCGA-MP-A4TF | 20 | 0 | 0 | 0 | 60 | FEMALE |
| TCGA-NJ-A55O | 30 | 50 | 20 | 0 | 0 | FEMALE |
| TCGA-78-7536 | 100 | 0 | 0 | 0 | 0 | MALE |
| TCGA-78-7147 | 100 | 0 | 0 | 0 | 0 | FEMALE |
| TCGA-99-7458 | 100 | 0 | 0 | 0 | 0 | FEMALE |
| TCGA-55-7573 | 50 | 40 | 0 | 0 | 10 | FEMALE |
| TCGA-93-7348 | 0 | 0 | 0 | 0 | 0 | FEMALE |
| TCGA-L9-A50W | 50 | 50 | 0 | 0 | 0 | MALE |
| TCGA-44-2657 | 40 | 40 | 0 | 0 | 20 | FEMALE |
| TCGA-55-A48Z | 0 | 0 | 0 | 0 | 0 | FEMALE |
| TCGA-50-8459 | 100 | 0 | 0 | 0 | 0 | MALE |
| TCGA-NJ-A7XG | 100 | 0 | 0 | 0 | 0 | MALE |
| TCGA-49-4501 | 30 | 70 | 0 | 0 | 0 | FEMALE |
| TCGA-49-4501 | 20 | 0 | 0 | 0 | 80 | FEMALE |
| TCGA-49-4501 | 20 | 30 | 10 | 40 | 0 | FEMALE |
| TCGA-86-8075 | 65 | 0 | 0 | 20 | 15 | FEMALE |
| TCGA-67-6217 | 50 | 50 | 0 | 0 | 0 | FEMALE |
| TCGA-MN-A4N4 | 70 | 0 | 5 | 15 | 10 | MALE |
| TCGA-MN-A4N4 | 80 | 10 | 10 | 0 | 0 | MALE |
| TCGA-55-6968 | 70 | 0 | 0 | 0 | 30 | MALE |
| TCGA-55-A48X | 0 | 0 | 0 | 0 | 0 | FEMALE |
| TCGA-55-1596 | 0 | 0 | 0 | 0 | 40 | MALE |
| TCGA-95-8039 | 0 | 90 | 10 | 0 | 0 | MALE |
| TCGA-86-8671 | 100 | 0 | 0 | 0 | 0 | FEMALE |
| TCGA-L9-A443 | 50 | 10 | 10 | 0 | 30 | FEMALE |
| TCGA-64-5778 | 30 | 65 | 5 | 0 | 0 | MALE |
| TCGA-55-8092 | 100 | 0 | 0 | 0 | 0 | MALE |
| TCGA-55-7995 | 20 | 0 | 0 | 0 | 80 | FEMALE |
| TCGA-55-6983 | 100 | 0 | 0 | 0 | 0 | MALE |
| TCGA-44-A4SU | 40 | 60 | 0 | 0 | 0 | FEMALE |
| TCGA-50-5946 | 60 | 0 | 10 | 0 | 10 | MALE |
| TCGA-NJ-A4YQ | 0 | 0 | 30 | 0 | 70 | FEMALE |
| TCGA-91-A4BD | 0 | 95 | 5 | 0 | 0 | MALE |
| TCGA-38-6178 | 70 | 0 | 10 | 0 | 0 | FEMALE |
| TCGA-86-7953 | 0 | 0 | 0 | 0 | 100 | FEMALE |
| TCGA-50-8457 | 90 | 0 | 0 | 0 | 10 | FEMALE |
| TCGA-49-AAR9 | 100 | 0 | 0 | 0 | 0 | MALE |
| TCGA-55-8506 | 0 | 0 | 0 | 0 | 0 | FEMALE |
| TCGA-05-4432 | 0 | 0 | 0 | 0 | 100 | MALE |
| TCGA-50-5068 | 100 | 0 | 0 | 0 | 0 | FEMALE |
| TCGA-50-5068 | 0 | 0 | 0 | 0 | 0 | FEMALE |
| TCGA-44-3398 | 0 | 0 | 0 | 0 | 100 | FEMALE |
| TCGA-44-2659 | 100 | 0 | 0 | 0 | 0 | FEMALE |
| TCGA-44-2655 | 100 | 0 | 0 | 0 | 0 | FEMALE |
| TCGA-99-8028 | 0 | 0 | 0 | 0 | 100 | FEMALE |
| TCGA-69-7761 | 90 | 0 | 5 | 0 | 0 | MALE |
| TCGA-55-8096 | 70 | 0 | 10 | 0 | 20 | FEMALE |
| TCGA-73-4662 | 20 | 50 | 5 | 0 | 25 | FEMALE |
| TCGA-05-4430 | 20 | 40 | 0 | 0 | 40 | FEMALE |
| TCGA-55-8208 | 60 | 0 | 10 | 0 | 30 | FEMALE |
| TCGA-44-6775 | 60 | 0 | 0 | 30 | 10 | FEMALE |
| TCGA-55-7907 | 100 | 0 | 0 | 0 | 0 | MALE |
| TCGA-05-5425 | 0 | 0 | 0 | 0 | 0 | MALE |
| TCGA-MN-A4N1 | 50 | 0 | 50 | 0 | 0 | MALE |
| TCGA-MN-A4N1 | 30 | 0 | 70 | 0 | 0 | MALE |
| TCGA-55-6971 | 70 | 0 | 0 | 0 | 30 | FEMALE |
| TCGA-L9-A444 | 20 | 30 | 10 | 0 | 40 | FEMALE |
| TCGA-44-3918 | 0 | 0 | 0 | 0 | 100 | FEMALE |
| TCGA-55-8505 | 0 | 0 | 0 | 0 | 0 | MALE |
| TCGA-93-A4JP | 0 | 70 | 30 | 0 | 0 | MALE |
| TCGA-44-3396 | 0 | 0 | 0 | 0 | 100 | FEMALE |
| TCGA-49-AAR4 | 0 | 0 | 0 | 0 | 100 | MALE |
| TCGA-78-8648 | 100 | 0 | 0 | 0 | 0 | FEMALE |
| TCGA-49-AAR2 | 10 | 90 | 0 | 0 | 0 | MALE |
| TCGA-05-5423 | 0 | 0 | 0 | 0 | 0 | MALE |
| TCGA-55-8207 | 80 | 0 | 0 | 20 | 0 | MALE |
| TCGA-97-A4M5 | 80 | 15 | 5 | 0 | 0 | MALE |
| TCGA-97-7553 | 30 | 50 | 10 | 10 | 0 | FEMALE |
| TCGA-55-7815 | 100 | 0 | 0 | 0 | 0 | MALE |
| TCGA-L4-A4E5 | 40 | 0 | 10 | 0 | 50 | FEMALE |
| TCGA-50-5045 | 10 | 10 | 0 | 0 | 80 | FEMALE |
| TCGA-67-6215 | 5 | 70 | 25 | 0 | 0 | FEMALE |
| TCGA-73-4677 | 25 | 50 | 5 | 0 | 20 | MALE |
| TCGA-44-6145 | 15 | 0 | 0 | 0 | 85 | FEMALE |
| TCGA-NJ-A55A | 80 | 0 | 0 | 10 | 10 | FEMALE |
| TCGA-49-6744 | 0 | 0 | 0 | 0 | 0 | FEMALE |
| TCGA-49-6744 | 0 | 0 | 0 | 0 | 0 | FEMALE |
| TCGA-49-6744 | 0 | 0 | 0 | 0 | 0 | FEMALE |
| TCGA-49-6744 | 15 | 80 | 5 | 0 | 0 | FEMALE |
| TCGA-NJ-A4YI | 50 | 40 | 10 | 0 | 0 | FEMALE |
| TCGA-86-6562 | 20 | 0 | 80 | 0 | 0 | MALE |
| TCGA-86-8056 | 0 | 100 | 0 | 0 | 0 | FEMALE |
| TCGA-86-A4JF | 50 | 0 | 30 | 0 | 20 | MALE |
| TCGA-S2-AA1A | 100 | 0 | 0 | 0 | 0 | FEMALE |
| TCGA-86-7714 | 80 | 20 | 0 | 0 | 0 | FEMALE |
| TCGA-55-7726 | 30 | 10 | 10 | 0 | 50 | FEMALE |
| TCGA-05-4384 | 0 | 0 | 0 | 0 | 0 | MALE |
| TCGA-L4-A4E6 | 15 | 0 | 10 | 0 | 70 | MALE |
| TCGA-99-8032 | 100 | 0 | 0 | 0 | 0 | MALE |
| TCGA-55-8091 | 5 | 0 | 0 | 0 | 95 | MALE |
| TCGA-78-8655 | 40 | 30 | 10 | 20 | 0 | FEMALE |
| TCGA-MP-A4TH | 60 | 10 | 0 | 20 | 0 | FEMALE |
| TCGA-44-7672 | 15 | 0 | 0 | 5 | 80 | FEMALE |
| TCGA-78-7148 | 70 | 30 | 0 | 0 | 0 | MALE |
| TCGA-99-8025 | 30 | 0 | 0 | 0 | 70 | FEMALE |
| TCGA-86-A4P8 | 40 | 50 | 10 | 0 | 0 | FEMALE |
| TCGA-49-AARO | 35 | 25 | 20 | 10 | 10 | FEMALE |
| TCGA-69-7979 | 50 | 0 | 0 | 0 | 50 | FEMALE |
| TCGA-MP-A4SW | 100 | 0 | 0 | 0 | 0 | MALE |
| TCGA-97-A4LX | 40 | 0 | 10 | 50 | 0 | MALE |
| TCGA-55-6981 | 10 | 0 | 0 | 0 | 90 | FEMALE |
| TCGA-35-5375 | 0 | 0 | 0 | 0 | 100 | MALE |
| TCGA-50-5044 | 0 | 0 | 0 | 0 | 0 | FEMALE |
| TCGA-44-7659 | 85 | 0 | 0 | 0 | 15 | MALE |
| TCGA-86-8278 | 100 | 0 | 0 | 0 | 0 | FEMALE |
| TCGA-44-3919 | 70 | 0 | 0 | 0 | 30 | FEMALE |
| TCGA-78-7156 | 0 | 100 | 0 | 0 | 0 | MALE |
| TCGA-05-4424 | 0 | 0 | 0 | 0 | 0 | MALE |
| TCGA-55-6980 | 60 | 30 | 10 | 0 | 0 | MALE |
| TCGA-NJ-A55R | 30 | 0 | 0 | 0 | 70 | MALE |
| TCGA-86-A4P7 | 0 | 80 | 10 | 10 | 0 | FEMALE |
| TCGA-NJ-A4YF | 70 | 0 | 30 | 0 | 0 | FEMALE |
| TCGA-78-7220 | 0 | 0 | 0 | 0 | 100 | FEMALE |
| TCGA-55-6982 | 60 | 0 | 0 | 0 | 40 | FEMALE |
| TCGA-78-7150 | 0 | 0 | 10 | 0 | 90 | MALE |
| TCGA-93-A4JQ | 20 | 0 | 0 | 30 | 50 | MALE |
| TCGA-69-7763 | 0 | 78 | 20 | 2 | 0 | MALE |
| TCGA-38-4627 | 100 | 0 | 0 | 0 | 0 | FEMALE |
| TCGA-05-4249 | 0 | 100 | 0 | 0 | 0 | MALE |
| TCGA-55-7281 | 40 | 50 | 10 | 0 | 0 | FEMALE |
| TCGA-86-8673 | 20 | 80 | 0 | 0 | 0 | MALE |
| TCGA-55-6979 | 0 | 0 | 0 | 0 | 100 | FEMALE |
| TCGA-44-6147 | 70 | 20 | 0 | 0 | 10 | FEMALE |
| TCGA-44-6777 | 30 | 0 | 10 | 0 | 60 | FEMALE |
| TCGA-55-8090 | 10 | 10 | 0 | 0 | 80 | MALE |
| TCGA-50-6593 | 30 | 0 | 0 | 0 | 70 | FEMALE |
| TCGA-MP-A4T7 | 50 | 0 | 0 | 0 | 50 | FEMALE |
| TCGA-05-4410 | 0 | 0 | 0 | 0 | 0 | MALE |
| TCGA-05-4418 | 20 | 0 | 0 | 0 | 80 | MALE |
| TCGA-64-1679 | 100 | 0 | 0 | 0 | 0 | FEMALE |
| TCGA-44-6146 | 0 | 0 | 0 | 0 | 0 | MALE |
| TCGA-69-8254 | 40 | 60 | 0 | 0 | 0 | MALE |
| TCGA-44-A47G | 50 | 0 | 0 | 20 | 0 | FEMALE |
| TCGA-MP-A4T9 | 0 | 0 | 0 | 0 | 100 | FEMALE |
| TCGA-97-7547 | 100 | 0 | 0 | 0 | 0 | FEMALE |
| TCGA-64-1680 | 80 | 15 | 5 | 0 | 0 | MALE |
| TCGA-80-5607 | 0 | 0 | 0 | 0 | 0 | FEMALE |
| TCGA-05-5429 | 0 | 0 | 0 | 0 | 0 | MALE |
| TCGA-05-4405 | 0 | 0 | 0 | 0 | 0 | FEMALE |
| TCGA-J2-8194 | 20 | 80 | 0 | 0 | 0 | FEMALE |
| TCGA-75-5125 | 0 | 0 | 0 | 0 | 0 | MALE |
| TCGA-44-8119 | 0 | 0 | 0 | 0 | 100 | MALE |
| TCGA-50-5055 | 0 | 0 | 0 | 0 | 0 | FEMALE |
| TCGA-50-5055 | 100 | 0 | 0 | 0 | 0 | FEMALE |
| TCGA-05-4390 | 0 | 0 | 0 | 0 | 0 | FEMALE |
| TCGA-86-8073 | 70 | 0 | 5 | 25 | 0 | MALE |
| TCGA-78-7633 | 0 | 25 | 25 | 50 | 0 | MALE |
| TCGA-44-7662 | 45 | 0 | 15 | 0 | 40 | MALE |
| TCGA-05-4426 | 0 | 30 | 0 | 0 | 70 | MALE |
| TCGA-49-6742 | 0 | 0 | 0 | 0 | 0 | MALE |
| TCGA-49-6742 | 95 | 0 | 5 | 0 | 0 | MALE |
| TCGA-49-6742 | 70 | 25 | 5 | 0 | 0 | MALE |
| TCGA-49-6742 | 80 | 10 | 10 | 0 | 0 | MALE |
| TCGA-49-6742 | 40 | 50 | 10 | 0 | 0 | MALE |
| TCGA-49-AAR3 | 0 | 0 | 0 | 0 | 100 | MALE |
| TCGA-35-3615 | 30 | 55 | 0 | 15 | 0 | MALE |
| TCGA-99-AA5R | 10 | 0 | 10 | 0 | 70 | FEMALE |
| TCGA-93-7347 | 60 | 10 | 0 | 30 | 0 | FEMALE |
| TCGA-97-7552 | 50 | 50 | 0 | 0 | 0 | MALE |
| TCGA-44-6148 | 100 | 0 | 0 | 0 | 0 | MALE |
| TCGA-53-7624 | 10 | 0 | 0 | 0 | 90 | FEMALE |
| TCGA-55-7724 | 50 | 0 | 10 | 0 | 40 | FEMALE |
| TCGA-73-4659 | 20 | 0 | 0 | 0 | 0 | MALE |
| TCGA-J2-A4AG | 60 | 40 | 0 | 0 | 0 | FEMALE |
| TCGA-05-4417 | 0 | 0 | 0 | 0 | 0 | FEMALE |
| TCGA-55-8513 | 0 | 0 | 0 | 0 | 0 | FEMALE |
| TCGA-55-A48Y | 0 | 0 | 0 | 0 | 0 | MALE |
| TCGA-38-4631 | 5 | 0 | 0 | 0 | 95 | FEMALE |
| TCGA-55-7816 | 50 | 0 | 50 | 0 | 0 | FEMALE |
| TCGA-44-A4SS | 5 | 0 | 0 | 0 | 95 | MALE |
| TCGA-97-A4M0 | 90 | 0 | 0 | 10 | 0 | FEMALE |
| TCGA-97-7546 | 5 | 0 | 0 | 95 | 0 | FEMALE |
| TCGA-55-6543 | 100 | 0 | 0 | 0 | 0 | FEMALE |
| TCGA-49-AARN | 60 | 0 | 0 | 0 | 40 | FEMALE |
| TCGA-55-6712 | 0 | 0 | 0 | 0 | 0 | MALE |
| TCGA-86-7701 | 60 | 0 | 0 | 0 | 40 | MALE |
| TCGA-86-8074 | 0 | 0 | 80 | 0 | 0 | FEMALE |
| TCGA-69-7765 | 20 | 0 | 5 | 25 | 50 | MALE |
| TCGA-55-8619 | 0 | 0 | 0 | 0 | 0 | FEMALE |
| TCGA-78-7539 | 50 | 0 | 50 | 0 | 0 | FEMALE |
| TCGA-86-8672 | 100 | 0 | 0 | 0 | 0 | MALE |
| TCGA-99-8033 | 100 | 0 | 0 | 0 | 0 | FEMALE |
| TCGA-MP-A4TA | 90 | 0 | 10 | 0 | 0 | FEMALE |
| TCGA-95-A4VP | 0 | 0 | 0 | 0 | 0 | FEMALE |
| TCGA-86-8674 | 70 | 30 | 0 | 0 | 0 | MALE |
| TCGA-78-7167 | 0 | 95 | 5 | 0 | 0 | MALE |
| TCGA-4B-A93V | 5 | 0 | 0 | 0 | 95 | FEMALE |
| TCGA-93-A4JO | 5 | 5 | 0 | 0 | 90 | MALE |
| TCGA-44-2665 | 20 | 0 | 0 | 0 | 0 | FEMALE |
| TCGA-MN-A4N5 | 50 | 0 | 0 | 0 | 50 | MALE |
| TCGA-78-8640 | 40 | 60 | 0 | 0 | 0 | MALE |
| TCGA-69-7973 | 0 | 0 | 0 | 0 | 100 | FEMALE |
| TCGA-69-A59K | 0 | 0 | 0 | 0 | 0 | FEMALE |
| TCGA-67-6216 | 20 | 10 | 30 | 0 | 40 | FEMALE |
| TCGA-MP-A4TK | 0 | 0 | 0 | 0 | 0 | FEMALE |
| TCGA-86-7713 | 0 | 60 | 40 | 0 | 0 | MALE |
| TCGA-97-A4M2 | 20 | 80 | 0 | 0 | 0 | MALE |
| TCGA-55-6970 | 30 | 60 | 10 | 0 | 0 | FEMALE |
| TCGA-86-8669 | 100 | 0 | 0 | 0 | 0 | MALE |
| TCGA-95-7039 | 0 | 0 | 0 | 0 | 0 | FEMALE |
| TCGA-97-7554 | 0 | 0 | 0 | 0 | 0 | FEMALE |
| TCGA-75-7030 | 0 | 0 | 0 | 0 | 0 | MALE |
| TCGA-55-7227 | 0 | 20 | 60 | 0 | 20 | MALE |
| TCGA-05-5420 | 0 | 0 | 0 | 0 | 0 | MALE |
| TCGA-55-6985 | 85 | 0 | 0 | 0 | 15 | FEMALE |
| TCGA-50-6673 | 80 | 0 | 20 | 0 | 0 | FEMALE |
| TCGA-78-7542 | 20 | 0 | 0 | 20 | 60 | MALE |
| TCGA-55-8089 | 5 | 5 | 0 | 0 | 95 | MALE |
| TCGA-49-4510 | 0 | 0 | 0 | 0 | 0 | FEMALE |
| TCGA-49-4510 | 0 | 95 | 5 | 0 | 0 | FEMALE |
| TCGA-35-4122 | 0 | 0 | 0 | 0 | 100 | MALE |
| TCGA-95-7562 | 0 | 0 | 0 | 0 | 0 | MALE |
| TCGA-44-2668 | 0 | 0 | 0 | 0 | 100 | MALE |
| TCGA-69-7974 | 20 | 0 | 0 | 0 | 80 | FEMALE |
| TCGA-78-7160 | 80 | 0 | 20 | 0 | 0 | MALE |
| TCGA-MP-A5C7 | 50 | 50 | 0 | 0 | 0 | FEMALE |
| TCGA-62-A471 | 0 | 0 | 0 | 0 | 0 | MALE |
| TCGA-50-6591 | 0 | 0 | 0 | 0 | 100 | FEMALE |
| TCGA-44-A47A | 20 | 0 | 10 | 0 | 70 | FEMALE |
| TCGA-55-6975 | 60 | 0 | 0 | 0 | 40 | MALE |
| TCGA-05-4244 | 20 | 0 | 20 | 0 | 60 | MALE |
| TCGA-64-5781 | 15 | 0 | 0 | 0 | 85 | FEMALE |
| TCGA-05-4398 | 0 | 0 | 0 | 30 | 70 | FEMALE |
| TCGA-49-AAR0 | 40 | 0 | 0 | 30 | 30 | MALE |
| TCGA-55-7574 | 80 | 0 | 0 | 0 | 20 | FEMALE |
| TCGA-44-7671 | 80 | 0 | 0 | 0 | 20 | MALE |
| TCGA-55-1594 | 0 | 0 | 0 | 0 | 100 | MALE |
| TCGA-05-4425 | 0 | 0 | 0 | 0 | 0 | FEMALE |
| TCGA-44-2662 | 0 | 0 | 0 | 0 | 100 | MALE |
| TCGA-05-4433 | 0 | 0 | 0 | 0 | 0 | MALE |
| TCGA-44-5644 | 0 | 0 | 0 | 0 | 100 | FEMALE |
| TCGA-49-4507 | 0 | 0 | 0 | 0 | 100 | FEMALE |
| TCGA-49-4507 | 0 | 0 | 0 | 0 | 0 | FEMALE |
| TCGA-L9-A743 | 50 | 10 | 0 | 0 | 40 | MALE |
| TCGA-05-4427 | 0 | 0 | 0 | 0 | 0 | FEMALE |
| TCGA-49-6767 | 0 | 0 | 0 | 0 | 0 | FEMALE |
| TCGA-55-6972 | 0 | 40 | 10 | 50 | 0 | MALE |
| TCGA-86-7711 | 0 | 0 | 0 | 0 | 100 | MALE |
| TCGA-MP-A4TI | 0 | 0 | 0 | 0 | 100 | MALE |
| TCGA-50-5066 | 0 | 0 | 5 | 0 | 95 | MALE |
| TCGA-55-8512 | 0 | 0 | 0 | 0 | 0 | MALE |
| TCGA-55-7903 | 0 | 5 | 0 | 0 | 95 | MALE |
| TCGA-78-7149 | 10 | 70 | 20 | 0 | 0 | MALE |
| TCGA-55-8620 | 0 | 0 | 0 | 0 | 0 | MALE |
| TCGA-55-8085 | 20 | 20 | 10 | 0 | 45 | MALE |
| TCGA-53-A4EZ | 0 | 0 | 0 | 0 | 100 | MALE |
| TCGA-50-5942 | 50 | 30 | 10 | 10 | 0 | FEMALE |
| TCGA-55-6978 | 0 | 0 | 0 | 0 | 100 | MALE |
| TCGA-05-4403 | 0 | 95 | 5 | 0 | 0 | MALE |
| TCGA-64-1677 | 100 | 0 | 0 | 0 | 0 | FEMALE |
| TCGA-62-A472 | 0 | 0 | 0 | 0 | 0 | MALE |
| TCGA-44-7670 | 40 | 60 | 0 | 0 | 0 | FEMALE |
| TCGA-95-7567 | 0 | 0 | 0 | 0 | 0 | MALE |
| TCGA-44-6774 | 100 | 0 | 0 | 0 | 0 | FEMALE |
| TCGA-49-4505 | 20 | 80 | 0 | 0 | 0 | FEMALE |
| TCGA-49-4505 | 0 | 0 | 0 | 0 | 0 | FEMALE |
| TCGA-49-4505 | 0 | 0 | 0 | 0 | 0 | FEMALE |
| TCGA-49-4505 | 10 | 70 | 20 | 0 | 0 | FEMALE |
| TCGA-64-5815 | 0 | 0 | 0 | 0 | 100 | MALE |
| TCGA-44-3917 | 0 | 0 | 0 | 0 | 100 | FEMALE |
| TCGA-49-4488 | 30 | 45 | 0 | 0 | 15 | FEMALE |
| TCGA-49-4488 | 5 | 45 | 5 | 35 | 10 | FEMALE |
| TCGA-49-4488 | 30 | 25 | 5 | 40 | 0 | FEMALE |
| TCGA-49-4488 | 25 | 50 | 5 | 10 | 5 | FEMALE |
| TCGA-49-4488 | 30 | 30 | 10 | 0 | 20 | FEMALE |
| TCGA-49-4488 | 10 | 50 | 10 | 10 | 20 | FEMALE |
| TCGA-49-4488 | 10 | 55 | 15 | 10 | 10 | FEMALE |
| TCGA-49-4488 | 5 | 50 | 15 | 30 | 0 | FEMALE |
| TCGA-49-4488 | 20 | 50 | 20 | 10 | 0 | FEMALE |
| TCGA-49-4488 | 30 | 40 | 20 | 0 | 0 | FEMALE |
| TCGA-38-4626 | 0 | 0 | 10 | 0 | 60 | FEMALE |
| TCGA-55-7576 | 70 | 0 | 0 | 30 | 0 | MALE |
| TCGA-55-8205 | 0 | 0 | 0 | 0 | 100 | FEMALE |
| TCGA-NJ-A4YG | 60 | 0 | 0 | 10 | 30 | MALE |
| TCGA-86-A456 | 60 | 30 | 10 | 0 | 0 | FEMALE |
| TCGA-MP-A4TD | 100 | 0 | 0 | 0 | 0 | MALE |
| TCGA-55-7994 | 0 | 0 | 0 | 0 | 100 | MALE |
| TCGA-44-4112 | 50 | 0 | 20 | 0 | 20 | FEMALE |
| TCGA-73-4658 | 75 | 5 | 10 | 0 | 10 | FEMALE |
| TCGA-55-6984 | 20 | 80 | 0 | 0 | 0 | FEMALE |
| TCGA-MP-A4T4 | 0 | 0 | 0 | 0 | 100 | FEMALE |
| TCGA-69-7760 | 0 | 100 | 0 | 0 | 0 | MALE |
| TCGA-73-4676 | 0 | 0 | 5 | 0 | 95 | MALE |
| TCGA-53-7626 | 80 | 0 | 0 | 0 | 20 | FEMALE |
| TCGA-44-7660 | 0 | 0 | 0 | 0 | 100 | MALE |
| TCGA-38-4629 | 0 | 0 | 0 | 0 | 100 | MALE |
| TCGA-44-2656 | 0 | 8 | 2 | 0 | 90 | MALE |
| TCGA-69-7978 | 0 | 0 | 0 | 0 | 100 | MALE |
| TCGA-55-7725 | 30 | 60 | 10 | 0 | 0 | FEMALE |
| TCGA-83-5908 | 40 | 0 | 0 | 0 | 60 | FEMALE |
| TCGA-44-7669 | 0 | 0 | 0 | 0 | 100 | MALE |
| TCGA-MP-A4TE | 0 | 30 | 0 | 0 | 0 | MALE |
| TCGA-97-A4M3 | 85 | 0 | 0 | 15 | 0 | FEMALE |
| TCGA-44-6778 | 20 | 0 | 0 | 0 | 80 | MALE |
| TCGA-73-4670 | 0 | 0 | 0 | 0 | 100 | FEMALE |
| TCGA-93-8067 | 50 | 20 | 5 | 0 | 25 | MALE |
| TCGA-78-7154 | 10 | 90 | 0 | 0 | 0 | MALE |
| TCGA-05-5428 | 0 | 0 | 0 | 0 | 0 | MALE |
| TCGA-05-4434 | 0 | 0 | 0 | 0 | 100 | FEMALE |
| TCGA-55-7911 | 20 | 20 | 10 | 0 | 50 | FEMALE |
| TCGA-86-8358 | 0 | 0 | 20 | 0 | 80 | MALE |
| TCGA-L9-A8F4 | 10 | 0 | 10 | 0 | 80 | FEMALE |
| TCGA-73-4666 | 0 | 0 | 0 | 0 | 100 | FEMALE |
| TCGA-64-1676 | 0 | 0 | 0 | 0 | 100 | MALE |
| TCGA-MP-A4SY | 60 | 40 | 0 | 0 | 10 | MALE |
| TCGA-38-4632 | 0 | 0 | 0 | 0 | 100 | MALE |
| TCGA-86-7955 | 15 | 0 | 5 | 0 | 80 | MALE |
| TCGA-MP-A4TC | 100 | 0 | 0 | 0 | 0 | MALE |
| TCGA-78-7145 | 0 | 60 | 10 | 0 | 30 | FEMALE |
| TCGA-NJ-A4YP | 100 | 0 | 0 | 0 | 0 | MALE |
| TCGA-55-7570 | 10 | 0 | 0 | 0 | 90 | MALE |
| TCGA-38-7271 | 0 | 0 | 0 | 0 | 0 | FEMALE |
| TCGA-55-A490 | 0 | 0 | 0 | 0 | 0 | MALE |
| TCGA-78-8660 | 0 | 0 | 60 | 0 | 40 | MALE |
| TCGA-75-6207 | 0 | 0 | 0 | 0 | 0 | MALE |
| TCGA-49-6761 | 20 | 0 | 0 | 0 | 80 | FEMALE |
| TCGA-73-4668 | 30 | 50 | 10 | 0 | 10 | FEMALE |
| TCGA-95-8494 | 0 | 0 | 0 | 0 | 0 | MALE |
| TCGA-50-5931 | 0 | 0 | 0 | 0 | 100 | FEMALE |
| TCGA-44-6779 | 100 | 0 | 0 | 0 | 0 | FEMALE |
| TCGA-49-6745 | 0 | 0 | 0 | 0 | 100 | MALE |
| TCGA-49-6745 | 0 | 0 | 0 | 0 | 0 | MALE |
| TCGA-49-6745 | 0 | 0 | 0 | 0 | 0 | MALE |
| TCGA-49-6745 | 0 | 0 | 0 | 0 | 0 | MALE |
| TCGA-49-6745 | 0 | 0 | 0 | 0 | 0 | MALE |
| TCGA-49-6745 | 0 | 0 | 0 | 0 | 0 | MALE |
| TCGA-49-6745 | 10 | 90 | 0 | 0 | 0 | MALE |
| TCGA-55-7283 | 0 | 50 | 10 | 0 | 40 | FEMALE |
| TCGA-75-5126 | 0 | 0 | 0 | 0 | 0 | FEMALE |
| TCGA-44-2666 | 30 | 40 | 5 | 25 | 0 | MALE |
| TCGA-05-4415 | 0 | 0 | 0 | 0 | 0 | MALE |
| TCGA-75-6214 | 0 | 0 | 0 | 0 | 0 | FEMALE |
| TCGA-86-8076 | 0 | 90 | 0 | 0 | 10 | MALE |
| TCGA-95-A4VN | 0 | 0 | 0 | 0 | 0 | FEMALE |
| TCGA-55-5899 | 0 | 0 | 0 | 0 | 100 | MALE |
| TCGA-49-4506 | 70 | 0 | 0 | 0 | 30 | FEMALE |
| TCGA-49-4506 | 0 | 0 | 0 | 0 | 0 | FEMALE |
| TCGA-49-4506 | 0 | 0 | 0 | 0 | 0 | FEMALE |
| TCGA-49-4506 | 0 | 0 | 0 | 0 | 0 | FEMALE |
| TCGA-55-6987 | 0 | 5 | 0 | 0 | 95 | MALE |
| TCGA-69-8255 | 30 | 0 | 0 | 0 | 70 | MALE |
| TCGA-35-4123 | 0 | 0 | 0 | 0 | 100 | MALE |
| TCGA-78-7152 | 30 | 40 | 20 | 0 | 0 | MALE |
| TCGA-50-6590 | 5 | 0 | 0 | 0 | 95 | FEMALE |
| TCGA-97-8171 | 0 | 0 | 0 | 0 | 0 | MALE |
| TCGA-64-5775 | 0 | 0 | 0 | 0 | 0 | MALE |
| TCGA-38-4625 | 5 | 0 | 0 | 5 | 90 | FEMALE |
| TCGA-05-4422 | 0 | 100 | 0 | 0 | 0 | MALE |
| TCGA-MP-A4T8 | 60 | 0 | 0 | 20 | 20 | MALE |

**Table S4** Oligonucleotide sequence in this study.

| **Primers for qRT-PCR** |  |
| --- | --- |
| BGN-F (Human) | CAGTGGCTTTGAACCTGGAG |
| BGN-R (Human) | GGGAGGTCTTTGGGGATGC |
| Bgn-F (Mouse) | GTGCCATGTGTCCTTTCGGT |
| Bgn-R (Mouse) | GCTTCCGCAGAGGGCTAAA |
| LAMC1-F (Human) | GGACTCCGCCCGAGGAATA |
| LAMC1-R (Human) | ACTTGAGACGCACATAGGTGA |
| Lamc1-F (Mouse) | TGCCGGAGTTTGTTAATGCC |
| Lamc1-R (Mouse) | TGGTTGTTGTAGTCGGTCAGG |
| LOXL1-F (Human) | CTGTGCTGCGGAGGAGAAG |
| LOXL1-R (Human) | GTAGTGGCTGAACTCGTCCA |
| Loxl1-F (Mouse) | GAGTGCTATTGCGCTTCCC |
| Loxl1-R (Mouse) | GGTTGCCGAAGTCACAGGT |
| WNT5A-F (Human) | ATTCTTGGTGGTCGCTAGGTA |
| WNT5A-R (Human) | CGCCTTCTCCGATGTACTGC |
| Wnt5a-F (Mouse) | CAACTGGCAGGACTTTCTCAA |
| Wnt5a-R (Mouse) | CCTTCTCCAATGTACTGCATGTG |
| MMP2-F (Human) | TACAGGATCATTGGCTACACACC |
| MMP2-R (Human) | GGTCACATCGCTCCAGACT |
| Mmp2-F (Mouse) | ACCTGAACACTTTCTATGGCTG |
| Mmp2-R (Mouse) | CTTCCGCATGGTCTCGATG |
| EPCAM-F (Human) | AATCGTCAATGCCAGTGTACTT |
| EPCAM -R (Human) | TCTCATCGCAGTCAGGATCATAA |
| Epcam-F (Mouse) | CTGGCGTCTAAATGCTTGGC |
| Epcam-R (Mouse) | CCTTGTCGGTTCTTCGGACTC |
| KRT8-F (Human) | CAGAAGTCCTACAAGGTGTCCA |
| KRT8-R (Human) | CTCTGGTTGACCGTAACTGCG |
| Krt8-F (Mouse) | TCCATCAGGGTGACTCAGAAA |
| Krt8-R (Mouse) | AAGGGGCTCAACAGGCTCT |
| TRPM7-F (Human) | ACTGGAGGAGTAAACACAGGT |
| TRPM7-R (Human) | TGGAGCTATTCCGATAGTGCAA |
| Trpm7-F (Mouse) | AGGATGTCAGATTTGTCAGCAAC |
| Trpm7-R (Mouse) | CCTGGTTAAAGTGTTCACCCAA |
| TRPC1-F (Human) | AGGATAGCCTCCGGCATTC |
| TRPC1-R (Human) | TTCCACCTCCACAAGACTTAGT |
| Trpc1-F (Mouse) | GATGTGCTTGGGAGAAATGCT |
| Trpc1-R (Mouse) | ACTGACAACCGTAGTCCAAAAG |
| TRPV2-F (Human) | TCAGGTTGGAGACATTAGATGGA |
| TRPV2-R (Human) | TCGGTAGTTGAGGTTGACTCTT |
| Trpv2-F (Mouse) | GACCGGAACTTCTCCCCTCA |
| Trpv2-R (Mouse) | TCAAACCGATTTGGGTCCTGT |
| TRPV4-F (Human) | GATGGGCGACCAAATCTGC |
| TRPV4-R (Human) | GAGGACTCATATAGGGTGGACTC |
| Trpv4-F (Mouse) | ATGGCAGATCCTGGTGATGG |
| Trpv4-R (Mouse) | GGAACTTCATACGCAGGTTTGG |
| PIEZO1-F (Human) | GGACTCTCGCTGGTCTACCT |
| PIEZO1-R (Human) | GGGCACAATATGCAGGCAGA |
| Piezo1-F (Mouse) | CACCTCCCTTTAGCCACGG |
| Piezo1-R (Mouse) | GGCAGCAACAGCCAGTAGA |
| ITGB1-F (Human) | CCTACTTCTGCACGATGTGATG |
| ITGB1-R (Human) | CCTTTGCTACGGTTGGTTACATT |
| Itgb1-F (Mouse) | ATGCCAAATCTTGCGGAGAAT |
| Itgb1-R (Mouse) | TTTGCTGCGATTGGTGACATT |
| ITGB6-F (Human) | TCCATCTGGAGTTGGCGAAAG |
| ITGB6-R (Human) | TCTGTCTGCCTACACTGAGAG |
| Itgb6-F (Mouse) | ATGGGGATTGAGCTGGTCTG |
| Itgb6-R (Mouse) | GACAGGTGGGTGAAATTCTCC |
| ITGB8-F (Human) | ACCAGGAGAAGTGTCTATCCAG |
| ITGB8-R (Human) | CCAAGACGAAAGTCACGGGA |
| Itgb8-F (Mouse) | TGCATGTTGTAACGTCAAGTGA |
| Itgb8-R (Mouse) | GATGCTGACACATCAACCAGATA |
| CCL7-F (Human) | AGGAGATCTGTGCTGACCCC |
| CCL7-R (Human) | GGGAGGAGCATCCCACAGTT |
| CCL8-F (Human) | TGGAGAGCTACACAAGAATCACC |
| CCL8-R (Human) | TGGTCCAGATGCTTCATGGAA |
| CDK6-F (Human) | GCTGACCAGCAGTACGAATG |
| CDK6-R (Human) | GCACACATCAAACAACCTGACC |
| VEGFA-F (Human) | AGGGCAGAATCATCACGAAGT |
| VEGFA-R (Human) | AGGGTCTCGATTGGATGGCA |
|  |  |
| **Primer for ChIP-PCR** |  |
| TWIST1-promoter-F | TCTAGGCCAGCAGAGCATA |
| TWIST1-promoter-R | CAAGGAAATTCGGGGAAGAGA |
| VIM-promoter-F | TGCTACTAAATCACGAGAAGACT |
| VIM-promoter-R | CTCTGGTCTCCTCTCGCTC |
| PKP1-promoter-F | GTGTTGTTTGCTTTAGGAGGC |
| PKP1-promoter-R | GTCTTTCTTGACCAGGCTGC |
| DSC2-promoter-F | GCCCACCTGACAGAAAGTTG |
| DSC2-promoter-R | TTCTGGCTCGGCATACAATG |
| VEGFA -promoter-F | CAACAGGTCCTCTTCCCTCC |
| VEGFA -promoter-R | CGCAATGAAGGGGAAGCTC |
| CDK6 -promoter-F | CTCTGTGCCTCTGTTTCTTCA |
| CDK6-promoter-R | AGTCCGGATGAAGGTATGTGA |
| CCL7 -motif1-F | AACTGGTCTCTTGATAATAGCCA |
| CCL7 -motif1-R | TCTGGAAGCTCTGTCTCTGC |
| CCL7 -motif2-F | TGTCGATAAATTTGCCTTCCCT |
| CCL7 -motif2-R | GTGTGTGTGTGTGTGTGTGT |
| CCL8 -motif1-F | AGTAAGTAGAAATGGTGGTTGCC |
| CCL8 -motif1-R | CCTCTCCACAAATCTCTGGC |
| CCL8 -motif2-F | TCCCAAAACCTGTCAAAACCC |
| CCL8 -motif2-R | TTAGGGGATGGTTCTCTGCC |
| CCL8 -motif3-F | ACAGGAGCATATTAACCACAAGA |
| CCL8 -motif3-R | TGCTGGAAAATTTGCCCCTT |
|  |  |
| **Primer&probe for DNase I** |  |
| TWIST1-promoter R1-F | TGATGTCTCATCTCGCCCAA |
| TWIST1-promoter R1-R | GGTTTGGCCTTTGGAACTCC |
| TWIST1-promoter R2-F | GTGGGAATTTGATAGCGCCT |
| TWIST1-promoter R2-R | TCACCAACTTTGCTGCTTCC |
| TWIST1-promoter R3-F | ATGGGCTTCTCAGTACGGAC |
| TWIST1-promoter R3-R | CAAGACCAAAGCTGCGAGTC |
| TWIST1-promoter R1 probe | AAGAAAAGTGTCAACCGCGG |
| TWIST1-promoter R2 probe | TCACCAACTTTGCTGCTTCC |
| TWIST1-promoter R3 probe | CTCAAGCTGAAGGCAAGAGC |
| VIM-promoter R1-F | GCCGCCAAAGATTCTGTCAT |
| VIM-promoter R1-R | CCCATGAAACCACACCCAAC |
| VIM-promoter R2-F | TTCCAGGCACACTCACAATG |
| VIM-promoter R2-R | CAGGACTCTCTACTGCCCAG |
| VIM -promoter R3-F | TTCACTTCTCTTGCAGCCCA |
| VIM -promoter R3-R | CTGGGAAATTGAGGCTGCAG |
| VIM -promoter R1 probe | CCCAATTTGATTAATGAGTTCA |
| VIM -promoter R2 probe | AGCCTATGCTCTGAGTTACATC |
| VIM -promoter R3 probe | AATGTATAAAAGTGTAGGTGTT |
| PKP1-promoter R1-F | GTACAGAGGGAGATGGACGC |
| PKP1-promoter R1-R | ATGAGCAAGAAACGTCGCAA |
| PKP1-promoter R2-F | AGTGACGGCTGTGATAGTGT |
| PKP1-promoter R2-R | GCCTCCTAAAGCAAACAACAC |
| PKP1-promoter R3-F | GGAAGCTGGATGATGTGGTG |
| PKP1 -promoter R3-R | GACTTCATCCAGCCAAGCAC |
| PKP1 -promoter R1 probe | TAGGAAAGAAAGGAGTCTTTCT |
| PKP1-promoter R2 probe | CTGAACTGTGTAGTCGTGCTGT |
| PKP1 -promoter R3 probe | GTGGCAAAGCCTGCATCCTAAC |
| DSC2-promoter R1-F | CGTGGGCTACATGTGGTTAC |
| DSC2-promoter R1-R | CTTCCAGATTGTGTGCTGCA |
| DSC2-promoter R2-F | TGAAAAGCCAACCCTGACAC |
| DSC2-promoter R2-R | CAGCTCATTAGTGGCATTCGT |
| DSC2-promoter R3-F | CTCAACACTCCCATTTGTCCA |
| DSC2 -promoter R3-R | TGTGGGCTTTGGATTGCAAA |
| DSC2 -promoter R1 probe | CGGACTCCTGAACATCCAGT |
| DSC2-promoter R2 probe | GTCTGTGCTATGCTCATGCC |
| DSC2 -promoter R3 probe | TGTGGGCTTTGGATTGCAAA |
| CDK6 -promoter R1-F | ATTTCCCTGAGGTCCTGTGC |
| CDK6 -promoter R1-R | TGAGGATGGGTGATGGGATG |
| CDK6 -promoter R2-F | GAGACAAGATGACGCTGTGC |
| CDK6 -promoter R2-R | TTTCTGCTGCTTTGCTCCTG |
| CDK6 -promoter R3-F | ACAGCACAAAGGAACAGCAC |
| CDK6 -promoter R3-R | CCCCTTGAAGCCTGGAAAGA |
| CDK6 -promoter R1 probe | TTTAGGGCTCTGACTTCCCC |
| CDK6 -promoter R2 probe | ATGTGGCCATCTTCTCCCTC |
| CDK6 -promoter R3 probe | CCTGCCAATAACCTCTGGGA |
| VEGFA -promoter R1-F | TCCAAAGCCCATTCCCTCTT |
| VEGFA -promoter R1-R | GGGAGAGGGACACACAGATC |
| VEGFA -promoter R2-F | CCGTTCTCAGCTCCACAAAC |
| VEGFA -promoter R2-R | TGGGACTGGAGTTGCTTCAT |
| VEGFA -promoter R3-F | TGGCTTCCCTTCCATATCCC |
| VEGFA -promoter R3-R | AGTCCTGTCTCCACCACTTG |
| VEGFA -promoter R1 probe | CTTCGAGAGTGAGGACGTGT |
| VEGFA -promoter R2 probe | ATTCTTCTCCCCTGGGAAGC |
| VEGFA -promoter R3 probe | ACAAAGACCTTGTCCCTGCT |
